# Can Growth Monitoring and Promotion Accurately Diagnose or Screen for Inadequate Growth of Individual Children? A Critical Review of the Epidemiologic Foundations

**DOI:** 10.1016/j.advnut.2025.100367

**Published:** 2025-01-11

**Authors:** Jef L Leroy, Rebecca L Brander, Edward A Frongillo, Leila M Larson, Marie T Ruel, Rasmi Avula

**Affiliations:** 1 International Food Policy Research Institute, Washington, DC, United States; 2 University of South Carolina, Columbia, South Carolina, United States; 3 International Food Policy Research Institute, New Delhi, Delhi, India

**Keywords:** growth monitoring and promotion, wasting, stunting, epidemiology, undernutrition, child growth, screening, diagnosis, prevention

## Abstract

Growth monitoring and promotion (GMP), the process of periodic anthropometric measurements to assess the adequacy of individual child growth, is implemented across low-income and middle-income countries. The epidemiologic foundations of GMP (i.e., that GMP can accurately diagnose or screen for inadequate growth) have never been critically reviewed. We first assessed growth patterns of individual healthy children. Using longitudinal data from low-income, middle-income, and high-income countries, we evaluated whether commonly used GMP criteria can be used for diagnosis and screening; i.e., whether they accurately identify current, or predict subsequent, inadequate growth in individual children. The growth of individual healthy children does not track along a specific growth curve, which challenges the notion that growth measurements alone can be used to distinguish between healthy and inadequate growth. We demonstrated that GMP criteria do not provide meaningful diagnostic information and that GMP is not a meaningful screening activity: commonly used GMP criteria are inaccurate predictors of (inadequate) growth later in childhood, and collecting individual children’s weight and height does not help to identify who needs support or who will benefit. Our results do not undermine the importance of dedicated programs to diagnose wasting in individual children nor do they challenge the need for well-child care to support parents and to ensure children’s optimal nutrition, health, and development. Our findings, however, highlight the need to carefully reconsider the current design of GMP in low-income and middle-income countries.

**Statement of Significance**

Our work demonstrates that common growth monitoring and promotion criteria in low-income and middle-income countries cannot accurately diagnose or screen for inadequate growth. Our findings do not challenge the need for timely diagnosis of children with wasting or the importance of well-child care.

## Introduction

Growth monitoring and promotion (GMP) follows the growth of children by periodic, frequent anthropometric measurements that are compared with an appropriate standard to assess the adequacy of the child’s growth. The measurements are visualized by plotting them on a growth chart and used for promotion activities in the form of tailored, individual counseling for parents [1]. Although there is no generally accepted set of GMP purposes, the literature identifies 3 of them, broadly, stated as [[1], [2], [3]]: diagnosis of or screening for inadequate individual child growth and growth promotion activities, population-level monitoring and surveillance, and promotion of the use of other health and nutrition services such as immunization and family planning.

Although growth monitoring and GMP programs have been widely implemented for many decades, their appropriateness and effectiveness have been questioned and challenged repeatedly since the 1980s [1,4,5]. Key controversial points relate to the lack of clarity on what growth monitoring and particularly promotion in GMP should entail [1], divergence of opinion about the specific purposes and hypothesized paths of impact of GMP [2,5], poor quality measurements potentially leading to incorrect diagnostics and other implementation constraints including in high-income countries [1,6,7], and low coverage [1,7].

A recent systematic review of the effect of GMP on anthropometric outcomes, infant and child feeding practices, and health service use found limited uncertain evidence on the effectiveness of GMP [8]. Only 6 studies were included, several of which suffered from methodologic limitations such as a small number of randomized clusters [9], lack of balance between arms at baseline [9], and lack of statistical power [10]. A study in Lesotho not included in the systematic review randomly assigned individual mothers to 1 of the 2 growth chart groups or to the no-chart group. All groups received nutrition counseling. Differences in outcomes between groups were small, but before and after comparisons showed large increases in maternal knowledge related to infant and young child feeding practices and diarrhea treatment in all groups, suggesting learning from nutrition counseling delivered [11]. Overall, however, the impact of GMP on caregiver nutrition knowledge, care practices, child growth, or the use of health and nutrition services is largely unknown. Likewise, there is no evidence on the use or usefulness of aggregated GMP data for decision making [2,[4], [5], [6], [7]].

The controversies around the use of GMP and the dearth of evidence on its effectiveness are in sharp contrast with the nearly universal implementation of growth monitoring across the globe [12,13]. The current widespread implementation of GMP appears to be mostly motivated by the belief that GMP is beneficial for children and their parents. The financial and economic costs are likely substantial, but reliable information on how much countries spend on GMP and on how these costs compare across countries is not available. Neither the World Bank’s reviews of public nutrition–related expenditures nor the Scaling up Nutrition initiative’s nutrition investment snapshots provide information on how much is spent on GMP [[14], [15], [16], [17]].

Notwithstanding the decades-old debate around GMP, the epidemiologic foundations of GMP have never been critically reviewed. Our study seeks to fill this evidence gap.

## Using Individual Children’s Weight and Height for Diagnosis and Screening

Inadequate growth, i.e., not gaining sufficient height or weight, occurs when children live in a deficient environment. These environments do not provide enough food for children or lack nutrient-rich foods, are often unsanitary, causing children to get sick repeatedly, and lack access to high-quality health services. These deficient environments are shaped by underlying problems of food insecurity, limited caregiving resources, and poor environmental conditions, which are affected by economic and social conditions and national and global contexts [18].

Poor growth is a marker of the deficient environment to which children have been or are currently exposed. Deficient environments can limit child growth and cause other short-term and long-term problems such as poor health, delayed child development, reduced earnings, and chronic diseases. Poor growth is therefore also a marker of future outcomes [19].

Inadequate growth could thus potentially be used to diagnose individual children whose environment is deficient and to provide them with interventions to normalize their growth. A second potential use is screening, i.e., to detect inadequate growth in individual children at an early stage and to intervene to prevent a further decline.

Regular anthropometric measurements to assess the adequacy of individual young children’s growth are common across the world. The assessments are conducted in different settings (e.g., community, primary health clinic, or individual physician’s or pediatrician’s office) and by different actors (e.g., community workers or trained health professionals). In high-income settings, children identified as not growing well are typically referred for a diagnostic workup to identify whether they experience an underlying health problem that needs to be addressed such as coeliac disease, Turner syndrome, or endocrinologic problems [6]. In many low-income country settings, referral is uncommon, and growth monitoring is often the only source of information for parents on the growth of their child. Our study focuses on GMP in these low-income settings where inadequate growth is often highly prevalent and caused by poor nutrition and health. Our objective was to assess the epidemiologic foundations of GMP. We do not study GMP implementation or coverage. Programs specifically designed to detect wasting and that assess weight-for-length or mid-upper arm circumference (MUAC) for diagnosis are also not the focus of our work.

We first assessed growth patterns of individual healthy children. Using data from low-income and middle-income countries (LMICs), we then evaluated whether commonly used GMP criteria are useful for diagnosis and screening, i.e., whether they accurately identify current or predict subsequent inadequate growth in individual children. Based on consultations with stakeholders, we also investigated whether GMP criteria are useful to predict subsequent wasting in individual children. The use of growth monitoring for population-level monitoring and surveillance is discussed briefly.

## Patterns of Growth in Healthy Children

**FIGURE 1:**
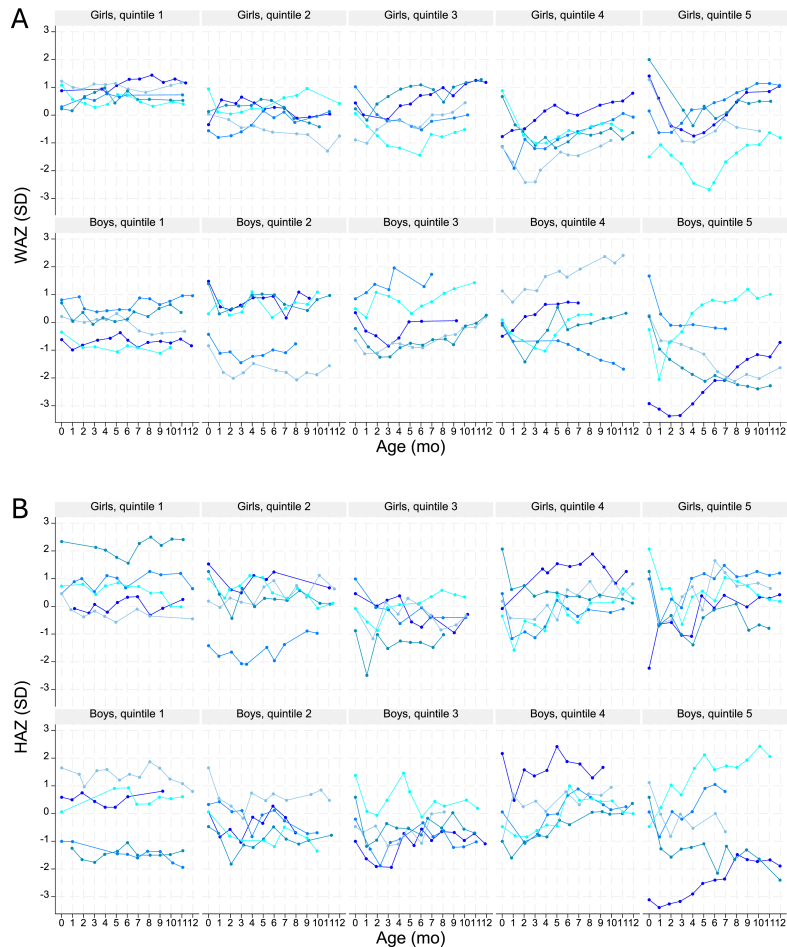
Growth patterns in weight and height in healthy growing children from the Flanders growth reference study [20]. We calculated the SDs of each child’s weight-for-age *z*-score (WAZ) and height-for-age *z*-score (HAZ) values and created SD-based quintiles. WAZ (A) and HAZ (B) are shown for 5 randomly selected infants in each quintile (different children are shown in the WAZ and HAZ graphs). Quintile 1 shows children who deviate the least from their own mean *z*-score; quintile 5 shows children with the largest variability in growth. If children tracked along a specific *z*-score line, the child-specific lines would be horizontal, i.e., have a zero slope.

For repeated weight and height measurements to be useful in detecting current or subsequent inadequate growth of individual children, growth patterns need to be sufficiently different between those children who grow well and those with inadequate growth. It is therefore illuminating to study the changes in weight and height of individual healthy children. We used data on 862 well-nourished and healthy children from the carefully conducted Flanders growth reference study [20,21] and calculated the weight-for-age *z*-score (WAZ) and height-for-age *z*-score (HAZ) for the measurements in the first 12 mo [22]. For each infant with ≥7 nonmissing observations, we calculated the SDs for WAZ and HAZ, which provided a measure of the dispersion around the infant-specific mean WAZ and HAZ, respectively (Supplemental text). We created SD-based quintiles and plotted WAZ and HAZ for 5 randomly selected infants in each quintile (Figure 1). None of the children, not even those in the quintiles with the least variable growth, tracked along a specific *z*-score line for weight or height. Patterns for WAZ and HAZ were highly variable with children repeatedly crossing *z*-score lines upward and downward in each of the quintiles. These results are in line with the WHO growth velocity standards, which show that growth velocities of individual healthy children are characterized by high variability [23] (Supplemental Table 1 for weight velocity and Supplemental Table 2 for height velocity). The lack of consistency in growth patterns across children and the common and repeated occurrence of intervals with decreasing *z*-scores in healthy growing children demonstrate that the use of weight and height measurements to identify children not growing well is challenging.

## GMP as a Diagnostic Activity

The first step to assess the utility of GMP as a diagnostic activity was to assess the proportion of children who meet commonly used GMP criteria for inadequate growth (Box 1). We used cohort data [20,24,25] from populations with a prevalence of underweight at 24 mo ranging from 0.7% (Belgium) to 37.7% (Bangladesh) (Table 1) (Supplemental Methods). We focused on children aged <24 mo because that is the period in which most of the linear growth faltering and wasting occurs [26,27]. Consequently, it is the age range with the highest recommended frequency of GMP visits (Supplemental Table 3). Nearly all children across the study sites experienced loss in WAZ or length-for-age *z*-score (LAZ) over a 1-mo period during infancy (Table 1). Even in Belgium, around 97% of children would be identified as having inadequate growth using these criteria. The probability of loss in WAZ or LAZ was lower for 2-mo and 3-mo periods but remained high even in the sites without stunting, wasting, or underweight (Belgium and Brazil). The proportion of infants meeting GMP criteria was not associated with the prevalence of being underweight or stunted at 24 mo. The challenge of discriminating between children growing well and children with inadequate growth is also reflected in the considerable overlap in the distribution of monthly weight gain across study sites and between these sites and the WHO growth standard (Supplemental Figure 1). There was no clear association between the distribution or median value of weight gain and the prevalence of underweight at 24 mo for either sex or at any of the time points. A first key problem with the use of GMP for diagnostic purposes is thus the difficulty of accurately distinguishing between children with healthy and inadequate growth.

BOX 1 **A comparison of GMP guidance and criteria from WHO, UNICEF, and 7 countries.**

We reviewed current growth monitoring and promotion (GMP) guidance from UNICEF, WHO, and 7 countries across South America, sub-Saharan Africa, and South Asia with well-established and notably strong GMP programs. We gathered information on country programs through internet searches and discussions with stakeholders involved with the program. Our review focused on the recommended frequency of growth assessment and the cutoffs used to assess a single weight or height measurement and those to assess changes in weight or length over a prespecified period.

Training materials from WHO and UNICEF provide different recommendations for the frequency of measurement (Supplemental Table 3). Only qualitative guidelines, also different for each organization, are provided to interpret changes over time. WHO states that crossing *z*-scores lines, having a flat line, and having a sharp incline or decline are indicative of possible problems [28]. UNICEF’s Facts for Life document states a line that stays flat or goes down is a cause for concern [29]. Note that a child’s height or weight could increase between measurements (ie, child would be identified as doing well based on UNICEF guidance) but that increase might be smaller than the expected growth during that interval, which would result in the child crossing *z*-score lines (ie, being classified as inadequately growing according to WHO guidance).

Country programs typically recommended more frequent assessment for younger compared with that for older ages, used weight-for-age *z*-score, and provided criteria for both single and repeated measurements. Countries vary substantially in the recommended frequency of measurement and in the specific GMP criteria used. In line with WHO guidance, most programs recommend monthly monitoring for children aged 0–24 mo. Ghana recommends monthly follow-up only for children aged 0–12 mo, and in India, weekly visits are recommended ≤1 mo and monthly visits ≤36 mo of age. Most programs flag measurements of weight-for-age *z*-score below −2 as a cause for concern. Guatemala, Ghana, and India also include criteria based on weight-for-length or height. Only the Guatemala program included instructions for measuring and interpreting length and height measurements. Although all program documents use language relating to the interpretation of the growth line or curve, specific instructions vary by program and tend to be ambiguously worded. Important details are left up to the interpretation of the program staff such as the number of measurements needed to constitute a trend, the number of consecutive measurements that are a cause for concern, and the magnitude of weight loss that would be a cause for concern.

Alt-text: BOX 1

**TABLE 1:** Prevalence of inadequate growth at 24 mo and of meeting GMP criteria during infancy in 11 cohort data sets [20,24,25].

	Belgium	Brazil	Peru	South Africa	Mali	Nepal	Tanzania	Bangladesh: Mirpur	India	Bangladesh: Matlab	Burkina Faso
	*n* = 854^1^	*n* = 233^1^	*n* = 303^1^	*n* = 314^1^	*n* = 1,132^1^	*n* = 240^1^	*n* = 262^1^	*n* = 265^1^	*n* = 251^1^	*n* = 3625^1^	*n* = 2113^1^
Prevalence at 24 mo^2^											
Underweight (%)	0.7	1.8	7.8	8.4	11.7	12.8	23.0	33.3	35.7	37.7	16.4
Wasting (%)	0.7	1.8	1.0	0.8	1.4	2.6	1.9	9.5	11.0	11.6	4.7
Stunting (%)	0.9	3.6	39.9	35.4	32.4	22.9	70.6	48.1	44.5	50.7	24.3
Common GMP criteria during infancy											
WAZ < −2 at any point (%)	0.7	6.4	19.1	24.8	30.0	20.8	25.6	40.0	49.4	42.0	34.7
Loss in WAZ over a 1-mo period (%)	96.7	95.3	94.1	89.2	99.5	98.8	98.1	96.6	96.4	95.2	100.0
Loss in WAZ over a 2-mo period (%)	68.3	76.8	82.8	76.7	68.3	89.6	89.7	82.3	89.2	66.4	94.7
Loss in WAZ over a 3-mo period (%)	31.1	42.1	52.5	44.9	27.3	58.8	53.8	52.8	59.0	31.9	67.5
Lack of weight gain over a 1-mo period (%)	28.2	58.4	59.1	66.6	85.2	66.3	88.9	64.9	64.9	77.1	94.7
Lack of weight gain over a 2-mo period (%)	0.8	6.0	6.6	15.3	16.6	7.5	30.9	7.2	8.4	12.9	31.5
Lack of weight gain over a 3-mo period (%)	0.0	0.4	0.3	1.3	1.3	0.0	3.4	0.0	0.4	1.5	5.7
Loss in LAZ over a 1-mo period (%)	97.8	95.3	97.4	90.1	100.0	99.2	97.7	96.2	96.8	96.2	99.8
Loss in LAZ over a 2-mo period (%)	68.8	82.8	89.8	78.7	85.8	90.8	91.6	88.3	88.8	70.0	93.4
Loss in LAZ over a 3-mo period (%)	27.5	49.8	59.7	52.2	50.3	63.3	53.4	56.6	55.0	31.7	54.6
Lack of length gain over a 1-mo period (%)	26.1	33.5	16.8	55.4	30.4	16.3	90.5	12.8	33.9	41.7	69.7
Lack of length gain over a 2-mo period (%)	0.9	1.8	0.7	5.7	0.9	0.8	18.7	0.4	1.2	2.0	3.9
Lack of length gain over a 3-mo period (%)	0.1	0.4	0.0	0.6	0.0	0.0	1.2	0.0	0.0	0.1	0.0

Anthropometric data were collected each month from 0 to 24 mo, except in Burkina Faso (0–18 mo) and Mali (6–24 mo). The shorter follow-up period in infancy in Mali affects the probability of meeting the GMP criteria. Countries are ordered by the prevalence of being underweight at 24 mo of age.

Abbreviations: GMP, growth monitoring and promotion; LAZ, length-for-age *z*-score; WAZ, weight-for-age *z*-score.

1 Actual numbers may vary due to missing data.

2 Values for Burkina Faso at 18 mo.

**FIGURE 2:**
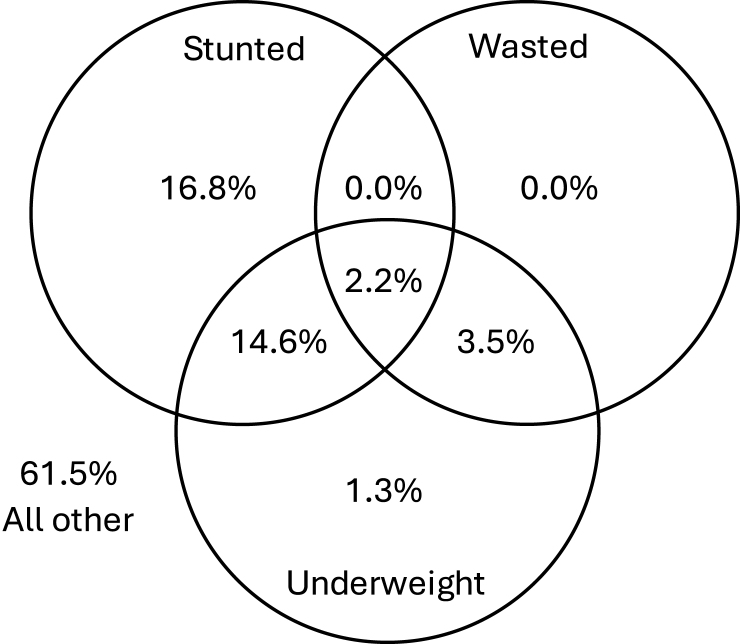
Overlap between child stunting, wasting, and underweight in children 12 mo of age in Bangladesh—Mirpur [24]. Stunting was defined as height-for-age *z*-score < −2, wasting as weight-for-height *z*-score < −2, and underweight as weight-for-age *z*-score < −2. All other refers to children who are not stunted, wasted, or underweight. Complete results are shown in Supplemental Figure 2.

A second problem is that it is not clear what GMP is seeking to diagnose. GMP programs typically collect child weight data to identify children with low weight-for-age or inadequate weight gain. These conditions are difficult to interpret because they can reflect that the child has low weight-for-height *z*-score (WHZ; and thus is thin) or that the child has low height-for-age (and thus is short), or a combination of both. In Bangladesh—Mirpur, for instance, 21.6% of the children were underweight: 5.7% were underweight and wasted, 16.8% were underweight and stunted, and 2.2% were underweight, wasted, and stunted (Figure 2 for Bangladesh—Mirpur, Supplemental Figure 2 for full results).

Wasting is a well-defined clinical condition for which internationally accepted treatment guidelines have been developed. Properly treated children usually return to normal weight within a few months. A key challenge is that GMP programs typically assess weight-for-age, which is sensitive but not specific to diagnose children with wasting: most wasted children are underweight, but most underweight children are not wasted (Figure 2, Supplemental Figure 2). To be effective at diagnosing children with wasting, GMP programs would need to include additional measurements such as height (to calculate weight-for-height) and MUAC [30].

**FIGURE 3:**
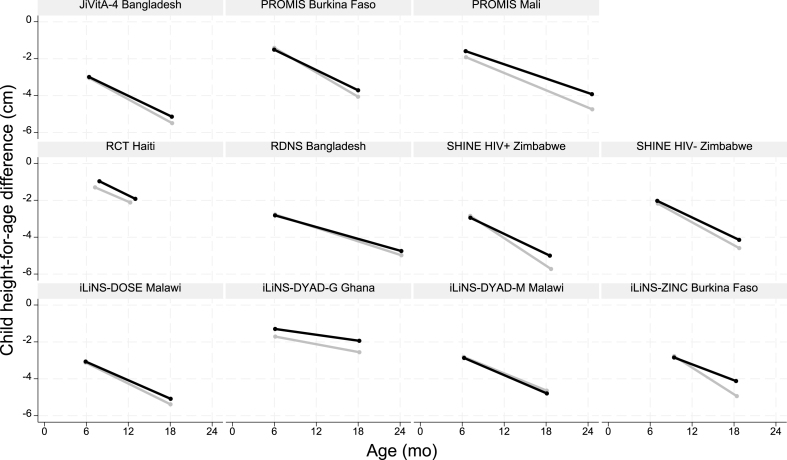
The effect of small-quantity lipid-based nutrient supplementation (SQ-LNS) on child linear growth in 11 RCTs [37]. Supplementation started after baseline in the treatment arm. Compliance—for definitions, see [37]—was as follows: PROMIS (Innovative Approaches for the Prevention of Childhood Malnutrition) Burkina Faso, 37%; PROMIS Mali, 47%; iLiNS-DOSE Malawi, 71.6%; iLiNS DYAD-G Ghana, 73.5%; SHINE Zimbabwe, 73.5%; iLiNS DYAD-M Malawi, 77.1%; JiVitA-4 Bangladesh, 93.0%; iLiNS-ZINC Burkina Faso, 96.8%; RCT HAITI, 97.0%; and RDNS (Rang-Din Nutrition Study) Bangladesh, 97.4%. Unadjusted means averaged for boys and girls are shown for the treatment (black) and control (gray) arms. Child height-for-age difference is the difference between the mean child length in the sample and the median length from the WHO growth standard [34]. The length deficit increased with age in both treatment and control arms. SQ-LNS had a positive effect on linear growth, but the effect was too small to reduce the size of the accumulated length deficit.

The diagnosis of linear growth faltering also comes with several challenges. First, most children with severe linear growth faltering (ie, with an HAZ below −2 SD) are not underweight (Figure 2, Supplemental Figure 2). If the GMP assessment is limited to measuring weight, most of the children with linear growth faltering will not be identified as growing poorly. Second, linear growth faltering or stunting, unlike wasting, does not have a known clinical meaning at the individual level [19]. It is nevertheless a (implicit) condition for which diagnosis occurs as demonstrated by the implementation of GMP in countries where wasting is virtually absent, the growing number of countries that include length measurements in GMP [13], the existence of guidance on how to interpret changes in length and height in GMP [28], and publications linking GMP to stunting prevention [31,32]. Third, in communities affected by undernutrition, the entire HAZ distribution is typically shifted to the left, and this downward shift is not accompanied by a widening of the height distribution [19,33]. This demonstrates that children across the entire height spectrum experience growth faltering. This finding is at odds with the objective of seeking to diagnose individual children to treat them. Finally, there is no available treatment that can bring the child back to the healthy linear growth trajectory, i.e., the trajectory that would make the health worker conclude that the child was growing well. Returning to a healthy linear growth trajectory requires the child to grow faster (i.e., at a higher velocity) than expected for their age and sex [34], which is referred to as catch-up growth. Carefully implemented and meticulously controlled nutrition interventions can improve linear growth [35], but the size of this growth impact is only a fraction of what is needed for catch-up growth [36]. Said differently, successful nutrition interventions can make children grow better relative to those who do not receive the intervention, but the magnitude of the effect is too small for children to reduce the size of the accumulated height deficit. We demonstrated this by looking at the effect of small-quantity lipid-based nutrient supplementation (SQ-LNS) on linear growth (Figure 3). SQ-LNS is considered one of the most effective currently available interventions to prevent linear growth faltering [37], but the effect is too small to reduce the size of the accumulated length deficit. Even in children treated with SQ-LNS, the length deficit increased with age. Consequently, children receiving SQ-LNS or any other effective nutrition intervention would continue to be identified as suffering from inadequate growth in GMP programs. If well-funded and carefully implemented nutrition interventions cannot bring children back to their healthy linear growth trajectory, then expecting that caregivers who are resource constrained could meaningfully improve the linear growth of their children even when presented with the best possible counselling is unreasonable. Counseling parents on strategies to improve feeding, health, and hygiene practices might benefit the nutrition and health status of the child but would not lead to improvements in linear growth large enough for the child to no longer be diagnosed as growing inadequately. In addition, increasing the weight of these underweight but nonwasted children may result in unhealthy weight gain [38]. We conclude that GMP as currently designed cannot be a meaningful diagnostic activity.

## GMP as a Screening Activity

Screening seeks to increase the chance of a positive health outcome in the future through the early detection of a health problem followed by a diagnosis and effective treatment [39]. Building on an earlier review, we assessed whether the requirements for a screening program are met in the case of GMP [7]. For screening to be useful, the epidemiologic relationships observed at the population level must be sufficiently strong to provide accurate prediction of outcomes of interest at the individual level. A first requirement for GMP to be useful for screening is thus that the assessment of growth allows for the early-stage detection of growth-related problems in individual children [1]. We assessed whether GMP criteria and growth velocity can accurately predict inadequate growth and wasting of individual children.

### Predicting subsequent inadequate growth

Using longitudinal data from 9 countries, we regressed WAZ and LAZ at 18 and 24 mo of age on anthropometric indices and growth velocity during infancy and used the root mean square error (RMSE) of each model to determine the predictive accuracy (details on the analytic methods in the Supplemental Methods). The RMSE reflects the mean difference between the predicted values and the observed values of the model and uses the same units as the dependent variable. Lower values indicate better predictive accuracy. We found large RMSEs in each site (most values were >0.7), which are indicative of poor predictive accuracy (Bangladesh—Mirpur results in Table 2; full results for anthropometric indices and growth velocity in Supplemental Tables 4 and 5, respectively). The results did not meaningfully differ by country.

**TABLE 2:** RMSE^1^ of regression models of anthropometric indices at 18 and 24 mo on anthropometric indices and weight and length velocity during infancy in Bangladesh—Mirpur.

	WAZ		LAZ	
	18 mo	24 mo	18 mo	24 mo
Measures at 1 time point				
WAZ at 3 mo	0.689	0.700	0.771	0.773
WAZ at 6 mo	0.574	0.587	0.714	0.707
WAZ at 12 mo	0.400	0.415	0.642	0.633
Weight at 3 mo (kg)	0.742	0.754	0.818	0.819
Weight at 6 mo (kg)	0.650	0.666	0.777	0.770
Weight at 12 mo (kg)	0.510	0.538	0.709	0.705
LAZ at 3 mo	0.862	0.862	0.698	0.720
LAZ at 6 mo	0.806	0.795	0.583	0.618
LAZ at 12 mo	0.718	0.712	0.401	0.440
Length at 3 mo (cm)	0.858	0.866	0.741	0.759
Length at 6 mo (cm)	0.807	0.811	0.650	0.679
Length at 12 mo (cm)	0.733	0.738	0.525	0.549
Weight velocity				
1-mo increments				
Weight velocity between 5 and 6 mo (kg/mo)	0.951	0.935	0.879	0.899
Weight velocity between 8 and 9 mo (kg/mo)	0.982	0.978	0.912	0.918
Weight velocity between 11 and 12 mo (kg/mo)	0.999	0.984	0.904	0.903
2-mo increments				
Weight velocity between 4 and 6 mo (kg/mo)	0.928	0.904	0.895	0.893
Weight velocity between 7 and 9 mo (kg/mo)	0.953	0.940	0.904	0.902
Weight velocity between 10 and 12 mo (kg/mo)	0.956	0.956	0.878	0.887
3-mo increments				
Weight velocity between 3 and 6 mo (kg/mo)	0.855	0.829	0.866	0.857
Weight velocity between 6 and 9 mo (kg/mo)	0.916	0.906	0.880	0.890
Weight velocity between 9 and 12 mo (kg/mo)	0.957	0.952	0.876	0.879
Length velocity				
1-mo increments				
Length velocity between 5 and 6 mo (cm/mo)	0.951	0.935	0.879	0.899
Length velocity between 8 and 9 mo (cm/mo)	0.982	0.978	0.912	0.918
Length velocity between 11 and 12 mo (cm/mo)	0.999	0.984	0.904	0.903
2-mo increments				
Length velocity between 4 and 6 mo (cm/mo)	0.928	0.904	0.895	0.893
Length velocity between 7 and 9 mo (cm/mo)	0.953	0.940	0.904	0.902
Length velocity between 10 and 12 mo (cm/mo)	0.955	0.958	0.880	0.888
3-mo increments				
Length velocity between 3 and 6 mo (cm/mo)	0.855	0.829	0.866	0.857
Length velocity between 6 and 9 mo (cm/mo)	0.916	0.906	0.880	0.890
Length velocity between 9 and 12 mo (cm/mo)	0.957	0.952	0.876	0.879

Abbreviations: GMP, growth monitoring and promotion; LAZ, length-for-age *z*-score; RMSE, root mean square error; WAZ, weight-for-age *z*-score.

1 The RMSE reflects the mean difference between the regression model’s predicted values and the observed values. It uses the same units as the dependent variable. The RMSE in the first cell (0.689) means that when predicting WAZ at 18 mo using the equation WAZ at 18 mo = *b*0 + *b*1 × WAZ at 3 mo, the 95% CI of the prediction of WAZ at 18 mo will be ∼2 times the RMSE or 1.378.

Using the same data sets, we calculated sensitivity, specificity, Youden index (sensitivity + specificity − 1), and the negative and positive predictive values of commonly used GMP criteria (WAZ < −2; loss in WAZ or LAZ over a 1-mo, 2-mo, or 3-mo period; lack of weight or length gain over a 1-mo, 2-mo, or 3-mo period). Our analyses found that these GMP criteria had poor predictive accuracy across all inadequate growth outcomes studied and in all study sites (select Bangladesh—Mirpur results in Table 3; full results in Supplemental Table 6). The highest sensitivity and specificity (and a Youden index of >0.50) across countries was found for underweight during infancy to predict underweight at 18 or 24 mo. Specificity and the Youden index for this criterion, however, were the highest for countries with the lowest prevalence of underweight. Sensitivity ranged from 61% to 90%, indicating that a substantial proportion of children who are underweight at 24 m were missed using the underweight criterion during infancy. For all other criteria, we found that criteria with high sensitivity had low specificity and vice versa. The positive (or negative) predictive value depended more strongly on the prevalence of the inadequate growth outcome than on the sensitivity (or specificity) of the criterion used.

**TABLE 3:** Predictive accuracy of GMP criteria for predicting underweight and stunting at 24 mo in Bangladesh—Mirpur.

	Sensitivity (%)	Specificity (%)	Youden index	Positive predictive value (%)	Negative predictive value (%)	Proportion meeting criterion (%)
Underweight at 24 mo						
WAZ < −2 at any point before 12 mo of age	78.6	80.7	0.59	67.1	88.3	39.0
Loss in WAZ over a 1-mo period	100.0	0.0	0.00	33.3	0.0	100.0
Loss in WAZ over a 2-mo period	97.1	15.0	0.12	36.4	91.3	89.0
Loss in WAZ over a 3-mo period	80.0	51.4	0.31	45.2	83.7	59.0
Lack of weight gain over a 1-mo period	75.7	30.0	0.06	35.1	71.2	71.9
Lack of weight gain over a 2-mo period	11.4	93.6	0.05	47.1	67.9	8.1
Lack of weight gain over a 3-mo period	0.0	100.0	0.00	0.0	66.7	0.0
Loss in LAZ over a 1-mo period	100.0	0.0	0.00	33.3	0.0	100.0
Loss in LAZ over a 2-mo period	97.1	3.6	0.01	33.5	71.4	96.7
Loss in LAZ over a 3-mo period	61.4	35.7	0.00	32.3	64.9	63.3
Lack of length gain over a 1-mo period	18.6	87.1	0.06	41.9	68.2	14.8
Lack of length gain over a 2-mo period	0.0	99.3	0.00	0.0	66.5	0.5
Lack of length gain over a 3-mo period	0.0	100.0	0.00	0.0	66.7	0.0
Stunting at 24 mo						
WAZ < −2 at any point before 12 mo of age	55.4	76.1	0.32	68.3	64.8	39.0
Loss in WAZ over a 1-mo period	100.0	0.0	0.00	48.1	0.0	100.0
Loss in WAZ over a 2-mo period	91.1	12.8	0.04	49.2	60.9	89.0
Loss in WAZ over a 3-mo period	68.3	49.5	0.18	55.6	62.8	59.0
Lack of weight gain over a 1-mo period	77.2	33.0	0.10	51.7	61.0	71.9
Lack of weight gain over a 2-mo period	9.9	93.6	0.03	58.8	52.8	8.1
Lack of weight gain over a 3-mo period	0.0	100.0	0.00	0.0	51.9	0.0
Loss in LAZ over a 1-mo period	100.0	0.0	0.00	48.1	0.0	100.0
Loss in LAZ over a 2-mo period	99.0	5.5	0.04	49.3	85.7	96.7
Loss in LAZ over a 3-mo period	65.3	38.5	0.04	49.6	54.5	63.3
Lack of length gain over a 1-mo period	19.8	89.9	0.10	64.5	54.7	14.8
Lack of length gain over a 2-mo period	1.0	100.0	0.01	100.0	52.2	0.5
Lack of length gain over a 3-mo period	0.0	100.0	0.00	0.0	51.9	0.0

Youden index is defined as sensitivity + specificity − 1.

Abbreviations: GMP, growth monitoring and promotion; LAZ, length-for-age *z*-score; WAZ, weight-for-age *z*-score.

Some of the children with inadequate growth in our data may have received treatment for wasting, which would attenuate the estimated predictive ability of the GMP criteria. When excluding children who had a weight-for-length *z*-score of <−2 at any time during infancy and who thus may have received an intervention to address wasting, our findings did not meaningfully change (Supplemental Table 7).

### Predicting subsequent wasting

Longitudinal data from Mali and Burkina Faso (collected to study wasting) were used to identify whether weight-related GMP criteria (WAZ < −2, WHZ < −2, loss in WAZ or WHZ or lack of weight gain in the preceding 1, 2, or 3 mo) can predict subsequent wasting in individual children. The predictive accuracy of the criteria we evaluated was poor across all ages in both Mali and Burkina Faso (Supplemental Table 8). Youden index was <0.50 for the majority of predictions. Underweight was not a good predictor of wasting at any timepoint. Its sensitivity ranged from 22% to 43% across ages in Burkina Faso and 17% to 71% in Mali. The MUAC and weight-for-length *z*-score–based metrics tended to be better predictors for subsequent wasting than other metrics, but high values for sensitivity were accompanied by low values for specificity and vice versa.

## Growth Monitoring for Population-Level Monitoring and Surveillance

Monitoring and surveillance of child nutritional status is important to justify the implementation of programs and to help with targeting these programs. Estimating the population prevalence of growth faltering, one of the often-stated purposes of GMP, does not require measuring all children. It can be done by collecting anthropometric data from a representative sample of children, which is considerably less costly than collecting growth data from all children as is expected in GMP. An additional challenge with relying on GMP data is the lack of representativeness because children taken to the GMP session are not necessarily representative of the entire population. Caregivers who take their child to GMP may be more educated, wealthier, and more health conscious than other caregivers in the same population, or the opposite; both scenarios would cause selection bias.

## Discussion

We critically reviewed the epidemiologic foundations of GMP. Individual healthy children follow highly variable growth trajectories characterized by frequent upward and downward crossings of *z*-score lines. These results are in line with previous evidence that individual children’s growth in weight and height is not continuous but occurs in spurts [40,41]. The erratic patterns are at odds with the commonly held belief that the growth of an individual child should track along a specific growth curve. Growth curves in standards or references do not show the expected changes in weight or height for the individual child. Growth standards or references are the result of mathematically smoothing age-specific *z*-score distributions [42] and show, for each age, which proportion of children in a healthy growing population has weight or height values above or below each curve. The highly irregular shape of individual growth patterns and the wide distribution of individual growth velocities among healthy children challenge the notion that growth information alone can be used to distinguish between healthy and inadequate growth of individual children. We discuss our findings related to each common use of GMP further.

### Weight and height measurements in GMP are not a useful diagnostic activity

Low weight-for-age or inadequate weight gain, 2 of the most used GMP criteria, do not provide a clear diagnosis because they do not distinguish between a child being too thin (and experiencing wasting) or too short for her or his age. For the diagnosis of wasting, weight-for-age is sensitive but not specific: most children experiencing wasting are also underweight, but a large proportion of underweight children do not show wasting. A wasting diagnosis requires measuring height in addition to weight or assessing MUAC [30]. Inadequate linear growth, on the contrary, is not a well-defined clinical condition for the individual child. Parents of a nonwasted underweight child will thus be told that something is wrong with their child although this diagnosis is not based on a well-defined clinical condition. In addition, there is no known effective treatment that allows parents to reduce the size of the child’s accumulated height deficit enough for the child’s growth to be considered adequate at a subsequent GMP visit.

### Measuring weight and height in GMP is not a useful screening activity

Commonly used GMP criteria are poor predictors of (inadequate) weight and height later in childhood. This result was found across study sites. Therefore, weight and height measurements cannot be used to identify which individual children will grow inadequately in the future. None of the criteria evaluated had useful predictive accuracy.

### GMP is not a meaningful population-level monitoring and surveillance activity

Our findings do not challenge the need for continued global monitoring and surveillance of child nutritional status, which remains important to justify programs and to help with targeting. Estimating the population prevalence of growth faltering using GMP, however, is more expensive and less statistically representative than surveying a sample of children.

GMP, as currently designed, does not have the epidemiologic basis needed to justify its widescale use as a diagnostic, screening, or monitoring and surveillance activity. This conclusion does not depend on the quality of implementation or on program coverage. Even if measurements were taken accurately, counseling was delivered carefully, and the entire target population was reached, GMP as currently designed would not be effective at improving the anthropometric outcomes it is assessing. Recent efforts across GMP programs to use artificial intelligence and other digital technologies to improve the quality of assessment, recording, plotting, or interpretation of measurements will not address the fundamental problems identified in our study.

An important strength of our study is our analysis of cohort data from different settings and use of a range of analytical methods to assess the predictive accuracy of commonly used GMP criteria. The data were collected as part of carefully conducted research projects, resulting in more accurate and precise data than those collected in routine GMP. We thus expect the low predictive accuracy we documented to be even lower if actual GMP data are used.

GMP has been presented as a strategy to address child stunting: international guidance exists on how to interpret length and height measurements [28], and several publications link the implementation of GMP to stunting prevention [31,32,43]. In addition, in many settings where GMP is implemented, the prevalence of wasting is low and therefore identifying underweight children amounts to identifying children with stunting. However, we find no evidence that GMP can contribute to the prevention of stunting or to reducing its prevalence.

GMP criteria do not accurately predict subsequent wasting. Our findings do not challenge the need for dedicated wasting programs, nor do we question the need to diagnose wasting in individual children in areas with a high incidence of this problem (this activity is sometimes incorrectly referred to as screening although it is a diagnostic activity). Children with moderate acute malnutrition and severe acute malnutrition are 3.4 and 11.6 times, respectively, more likely to die [44]. An estimated 13% of deaths in children aged <5 y are due to wasting [45]. Effective treatments exist for wasting, so diagnosing wasted children in a timely fashion has the potential to save lives [30]. Simply measuring child weight as GMP programs typically do, however, does not provide the information needed to diagnose wasting. Detecting children with wasting requires assessing weight-for-length or MUAC [30]. The detection should be followed by referral for treatment, careful follow-up to ensure that children initiate and adhere to treatment, and the prevention of relapse once the child has recovered.

A key aspect to consider is the potential harm generated by GMP. As explained, low weight-for-age not caused by wasting is due to linear growth faltering, which is not reversible with currently available nutrition interventions. Many parents are thus bound to hear month after month that their child is not doing well, with GMP creating the illusion that they can do better for their children. Not being able to solve the purported problem with which they are presented, no matter how hard they try, is likely to cause emotional stress and anxiety.

Our results do not challenge the need for well-child care in LMICs. Providing guidance to parents on infant and young child feeding, responsive and age-appropriate play and communication, the prevention of illness, and timely care-seeking may be beneficial to children and their parents, and such guidance should be part of well-baby visits. However, our results demonstrate that collecting individual children’s weight and height does not help to identify who needs support and who will benefit. In addition, the anthropometric measures are unlikely to respond to these interventions, i.e., they provide no information on whether parents implemented the recommended actions and whether their children benefited. Research is needed to identify how regular meetings with parents can be optimized to improve the nutrition, health, and development of their young children in LMICs.

Recommending to reconsider GMP in LMICs while regular anthropometric assessment of individual children is common in many high-income countries (HICs) may be viewed as unethical, but the 2 contexts have important differences. First, inadequate growth in infants and young children in LMICs is mostly due to deficient environments, i.e., environments that do not provide the nutrition and health inputs children need to grow and develop [19]. Because most children in these settings are exposed to the same unfavorable conditions, assessing growth in individual children does not provide more information about which actions are needed to improve child wellbeing than simply collecting data on a sample of children. Second, children in HICs who are identified as not growing well are typically referred for a diagnostic workup to identify which underlying health issue (if any) causes the growth problem [6]. In low-income populations, referral would be impractical and highly cost-ineffective due to the large number of children experiencing inadequate growth. Furthermore, even in HICs, there is no agreement on which algorithm to use to identify children with inadequate growth. Many of the algorithms used in clinical practice have either not been carefully validated or low sensitivity and specificity [6].

Growing up in a deficient environment has profound long-term negative effects on the health, development, and opportunities of individuals and societies [19]. Investing in the nutrition, health, and development of young children is thus imperative. Our findings highlight the need to carefully reconsider how GMP can best contribute to this objective, which should be done through additional research and close engagement with key stakeholders (e.g., international agencies, government agencies, in-country stakeholders, and program implementers). Critical steps in this process will be to identify meaningful purposes of this activity, to determine which actions are needed to meet the objectives, and to ensure adequate recruitment, training, remuneration, and supervision of the cadre of staff implementing these actions. If individual assessments are needed, they should reflect what is critical for the child’s wellbeing, be limited to what parents can feasibly change, and be responsive to improvements in the child’s environment. Growth assessment could be considered, not as a basis for making decisions, but to inform interested parents and if it generates an opportunity to engage parents in discussion about how to foster the child’s nutrition, health, and development.

## Conflict of interest

The authors report no conflicts of interest.

## Data sharing

The findings of this analysis were supported by a combination of data from multiple principal investigators and institutions. The data are available, upon reasonable request, to the requestor by contacting the individual principal investigators.
