# Supplementary material for: Can Growth Monitoring and Promotion Accurately Diagnose or Screen for Inadequate Growth of Individual Children? A Critical Review of the Epidemiologic Foundations
Source: Adv Nutr. 2025 Jan 11;16(3):100367. doi: 10.1016/j.advnut.2025.100367 (PMC11880696; doi:10.1016/j.advnut.2025.100367)
Supplement: Multimedia component 1 [file mmc1.docx]

**Perspective: Can growth monitoring and promotion work for diagnosis and screening? A critical review of the epidemiological foundations**

Jef L Leroy et al.

[**Supplementary text - Methods** 2](#_Toc175816037)

[**Supplementary Table 1**: WHO growth velocity standard for weight in girls and boys from 0 to 12 mo of age 5](#_Toc175816038)

[**Supplementary Table 2**: WHO growth velocity standard for length in girls and boys from 0 to 12 mo of age 6](#_Toc175816039)

[**Supplementary Table 3**: GMP guidance and criteria^a^ 7](#_Toc175816040)

[**Supplementary Table 4**: Root mean square error (RMSE) of regression models of anthropometric indices at 18 and 24 months on anthropometric indices during infancy in Brazil, Peru, South Africa, Mali, Nepal, Tanzania, Bangladesh-Mirpur, India, Bangladesh - Matlab, and Burkina Faso. 9](#_Toc175816041)

[**Supplementary Table 5**: Root mean square error (RMSE) of regression models of anthropometric indices at 18 and 24 months on growth velocity during infancy in Brazil, Peru, South Africa, Mali, Nepal, Tanzania, Bangladesh-Mirpur, India, Bangladesh - Matlab, and Burkina Faso. 10](#_Toc175816042)

[**Supplementary Table 6:** Predictive accuracy of GMP criteria for predicting subsequent inadequate growth in Brazil, Peru, South Africa, Mali, Nepal, Tanzania, Bangladesh-Mirpur, India, Bangladesh - Matlab, and Burkina Faso. 12](#_Toc175816043)

[**Supplementary Table 7:** Predictive accuracy of GMP criteria for predicting subsequent inadequate growth after excluding children with low weight-for-length z-score in Brazil, Peru, South Africa, Mali, Nepal, Tanzania, Bangladesh-Mirpur, India, Bangladesh - Matlab, and Burkina Faso. 19](#_Toc175816044)

[**Supplementary Table 8**: Predictive accuracy of weight-related GMP, MUAC, and WLZ-based criteria for predicting acute malnutrition in Mali and Burkina Faso among children without acute malnutrition at the time of measurement. 26](#_Toc175816045)

[**Supplementary Figure 1**: Distribution of one-month weight gain in boys (panels A to D) and girls (E to H) relative to the WHO growth standard between 2 and 3 months (A and E), 5 to 6 months (B and F), 8 to 9 months (C to G), and 11 to 12 months (D to H). 35](#_Toc175816046)

[**Supplementary Figure 2**: Overlap between child stunting, wasting, and underweight in children 12 months of age in Belgium (A), Brazil (B), Peru (C), South Africa (D), Mali (E), Nepal (F), Tanzania (G), Bangladesh - Mirpur (H), India (I), Bangladesh - Matlab (J), and Burkina Faso (K). 38](#_Toc175816047)

[**References** 40](#_Toc175816048)

# **Supplementary text - Methods**

**Data**

Cohort data from well-nourished and undernourished populations were used. The Flanders growth reference study enrolled 862 children at birth and followed them monthly to 12 months of age and then quarterly to 36 months of age (1). All children were singletons, born between 37 and 42 weeks of gestation, with parents and grandparents of Belgian origin, free from known growth disorders or severe chronic disease, and not using any medications known to affect growth.

The Malnutrition and Enteric Disease Study (MAL-ED) observational data were collected in Brazil, Peru, South Africa, Tanzania, India, Pakistan, Bangladesh (Mirpur), and Nepal (2). No information is available on which children participated in GMP. Data from the Pakistan site were excluded due to concerns about the quality of the length/height data (3,4). Children were enrolled within 17 days of birth and anthropometric measurements were taken twice monthly by trained study staff through 24 months of age. Excluding data from the Pakistan site, the sample size of MAL-ED at enrollment was 1,868 newborns, approximately evenly distributed across the 7 sites. The areas where the MAL-ED study was conducted had some GMP program activities ongoing but were poorly implemented at all sites, as per discussions with MAL-ED study investigators.

The Maternal and Infant Nutrition Interventions in Matlab (MINIMat) study randomly allocated pregnant women to one of six treatment arms that varied in the micronutrient and food supplements received. MINIMat collected longitudinal data on 4436 pregnant women and their children (3625 live births) living in Matlab, Bangladesh (5,6). Children’s weight and length were measured monthly from birth until 12 months of age and then quarterly until 24 months of age.

The Integrated Prevention and Treatment of Child Malnutrition and Health (PROMIS) study tested the effectiveness of a package of interventions to prevent wasting in Mali and Burkina Faso using a cluster-randomized design (7). In PROMIS-Mali, children 6 months of age were enrolled and followed monthly for 18 months through 23 months of age. In PROMIS-Burkina Faso, children were enrolled within 6 weeks of birth and followed monthly for 18 months through 17 months of age, but our analyses is limited to 6 through 17 months of age. Anthropometric measurements were taken at each monthly follow up visit by trained study staff (8,9). Only data from the control arm were used in the analyses. Children in the control arm received the standard of care which was comprised of screening and referral for wasting and behavior change communication on essential nutrition and health actions. Children being treated for wasting were excluded from the sample.

**GMP criteria used to screen for inadequate growth**

The GMP criteria used in the analyses were based on our review of GMP guidance (Box 1, Table 1) and defined as follows:

- Weight-for-age Z-score (WAZ) < -2 at any point during infancy (0-12 months of age)
- Decrease in WAZ for 1, 2, or 3 consecutive months, i.e., ΔWAZ < 0 for 2, 3, or 4 consecutive monthly measurements at any point during infancy
- Lack of weight gain for 1, 2, or 3 consecutive months, i.e., weight gain ≤ 0 for 2, 3, or 4 consecutive monthly measurements at any point during infancy
- Decrease in length-for-age z-score (LAZ) for 1, 2, or 3 consecutive months, i.e., ΔLAZ < 0 for 2, 3, or 4 consecutive monthly measurements at any point during infancy
- Lack of length gain for 1, 2, or 3 consecutive months, i.e., length gain ≤ 0 for 2, 3, or 3 consecutive monthly measurements at any point during infancy

**Outcome definitions of inadequate growth in children 12 to 24 months of age**

Inadequate child growth was defined as low weight-for-age (the explicit focus of GMP programs) and as low length-for-age (the implicit focus of many programs):

- Underweight (WAZ < -2) at 18 or 24 months of age;
- Decrease in WAZ of any magnitude between age 12 to 18 months, 18 to 24 months, or 12 to 24 months;
- Stunting (LAZ < -2) at 18 or 24 months of age;
- Decrease in LAZ of any magnitude between age 12 to 18 months, 18 to 24 months, or 12 to 24 months;
- Decrease in length-for-age difference (LAD) of any magnitude between age 12 to 18 months, 18 to 24 months, or 12 to 24 months (10).

**Criteria used to screen for acute malnutrition**

There are no commonly used criteria to predict the occurrence of acute malnutrition in individual children. We evaluated the predictive of ability of the weight-based criteria used in GMP programs and added WLZ- and MUAC-based criteria as these are commonly used to diagnose acute malnutrition.

- WAZ < -2 in the month preceding the month of the outcome measurement;
- Decrease in WAZ for 1, 2, or 3 consecutive months, i.e., ΔWAZ < 0 for 2, 3, or 4 consecutive monthly measurements preceding the month of the outcome measurement;
- Lack of weight gain for 1, 2, or 3 consecutive months, i.e., weight gain ≤ 0 for 2, 3, or 4 consecutive monthly measurements preceding the month of the outcome measurement;
- Weight-for-length (WLZ) < - 1 or <-1.5 in the month preceding the month of the outcome measurement;
- Decrease in WLZ for 1, 2, or 3 consecutive months, i.e., ΔWLZ < 0 for 2,3, or 4 consecutive monthly measurements preceding the month of the outcome measurement;
- Mid-upper arm circumference (MUAC) < 130 mm in children older than 6 mo of age, at the month preceding the month of the outcome measurement;
- Decrease in MUAC for 1,2, or 3 consecutive months in children older than 6 mo of age, i.e., ΔMUAC < 0 for 2, 3, or 4 consecutive monthly measurements preceding the month of the outcome measurement.

**Outcome definition of acute malnutrition**

Acute malnutrition was defined as MUAC < 125 mm (as all children included in the analyses were 6 months or older), WHZ < -2, or presence of bilateral pitting edema.

**Data analysis**

Implausible values for anthropometry (LAZ/HAZ < –6 or > +6, WLZ/WHZ < –5 or > +5, and WAZ < –6 or > +5) were dropped from the analyses (11). All analyses were site-specific, i.e., data were not pooled across sites.

We compared the distribution of 1-, 2-, and 3-month weight increments (using boxplots) to the WHO weight velocity standards. We calculated the prevalence of meeting the GMP criteria in infancy and the prevalence of growth retardation in the second year of life using the definitions described above.

Linear regression models were used to examine the association between (continuous) growth status (WAZ and LAZ), before and after 12 months of age, and between weight and length velocity over 1-, 2-, and 3-month increments before 12 months of age and growth status (WAZ and LAZ) after 12 months of age. The root mean square error (RMSE) of each model was used to determine the predictive accuracy. The RMSE reflects the mean difference between the model’s predicted values and the observed values and uses the same units as the dependent variable. Lower values indicate better predictive accuracy.

The predictive accuracy of each GMP criterion was assessed by calculating the sensitivity, specificity, Youden’s index (i.e., sensitivity + specificity – 1), positive predictive value (PPV), and negative predictive value (NPV) of each criterion for predicting subsequent inadequate growth. Some of the children with inadequate growth in our data may have received a nutrition intervention. If that intervention improved their growth, the estimated association and predictive ability could be attenuated. We conducted a sensitivity analysis excluding children who had WLZ < -2 at any time during infancy as these children may have received an intervention to address acute malnutrition which is known to lead to subsequent weight gain.

The acute malnutrition analyses were conducted separately for each month of age in the 6- to 24-month age range and evaluated the predictive accuracy of the screening criteria for the presence of acute malnutrition one month later. We excluded children with acute malnutrition in the screening period, children who were under treatment for acute malnutrition per caregiver report in the screening period, and children who were under treatment for acute malnutrition per caregiver report at the time of outcome measurement. In Burkina Faso, where children were enrolled in the parent study at birth, we excluded timepoints under 6 months of age because children in this age range were not eligible for outpatient wasting treatment and thus treatment is defined differently for this age group. The sensitivity, specificity, PPV, and NPV of each criterion were calculated.

# **Supplementary Table 1**: WHO growth velocity standard for weight in girls and boys from 0 to 12 mo of age

| **Age** | **-3 SD** | **-2 SD** | **-1 SD** | **Median** | **1 SD** | **2 SD** | **3 SD** |
| --- | --- | --- | --- | --- | --- | --- | --- |
| Girls |  |  |  |  |  |  |  |
| 0 – 4 wk, g | 123 | 358 | 611 | 879 | 1161 | 1453 | 1757 |
| 4 wk – 2 mo, g | 251 | 490 | 744 | 1011 | 1290 | 1580 | 1880 |
| 2 – 3 mo, g | 105 | 297 | 502 | 718 | 944 | 1178 | 1421 |
| 3 – 4 mo, g | 14 | 192 | 383 | 585 | 796 | 1016 | 1244 |
| 4 – 5 mo, g | -62 | 108 | 293 | 489 | 695 | 911 | 1134 |
| 5 – 6 mo, g | -132 | 31 | 210 | 401 | 604 | 815 | 1036 |
| 6 – 7 mo, g | -185 | -24 | 153 | 344 | 547 | 760 | 982 |
| 7 – 8 mo, g | -224 | -64 | 116 | 311 | 519 | 738 | 967 |
| 8 – 9 mo, g | -259 | -101 | 77 | 273 | 482 | 702 | 933 |
| 9 – 10 mo, g | -286 | -131 | 48 | 245 | 456 | 679 | 913 |
| 10 – 11 mo, g | -307 | -151 | 31 | 233 | 451 | 682 | 924 |
| 11 – 12 mo, g | -324 | -166 | 22 | 232 | 458 | 699 | 953 |
| Boys |  |  |  |  |  |  |  |
| 0 – 4 wk, g | -160 | 321 | 694 | 1023 | 1325 | 1608 | 1876 |
| 4 wk – 2 mo, g | 354 | 615 | 897 | 1196 | 1512 | 1844 | 2189 |
| 2 – 3 mo, g | 178 | 372 | 585 | 815 | 1061 | 1322 | 1597 |
| 3 – 4 mo, g | 44 | 219 | 411 | 617 | 837 | 1069 | 1313 |
| 4 – 5 mo, g | -45 | 128 | 318 | 522 | 738 | 965 | 1202 |
| 5 – 6 mo, g | -128 | 40 | 224 | 422 | 632 | 853 | 1083 |
| 6 – 7 mo, g | -183 | -21 | 161 | 357 | 565 | 785 | 1014 |
| 7 – 8 mo, g | -223 | -63 | 118 | 316 | 528 | 752 | 987 |
| 8 – 9 mo, g | -256 | -98 | 84 | 285 | 500 | 729 | 969 |
| 9 – 10 mo, g | -286 | -128 | 55 | 259 | 478 | 711 | 956 |
| 10 – 11 mo, g | -312 | -153 | 34 | 243 | 469 | 710 | 963 |
| 11 – 12 mo, g | -333 | -172 | 22 | 239 | 475 | 726 | 990 |

# **Supplementary Table 2**: WHO growth velocity standard for length in girls and boys from 0 to 12 mo of age

| **Age** | **-3 SD** | **-2 SD** | **-1 SD** | **Median** | **1 SD** | **2 SD** | **3 SD** |
| --- | --- | --- | --- | --- | --- | --- | --- |
| Girls |  |  |  |  |  |  |  |
| 0-2 mo, cm | 4.6 | 5.7 | 6.8 | 7.9 | 9 | 10.1 | 11.3 |
| 1-3 mo, cm | 3.5 | 4.5 | 5.4 | 6.4 | 7.3 | 8.3 | 9.3 |
| 2-4 mo, cm | 2.4 | 3.3 | 4.2 | 5.2 | 6.1 | 7 | 7.9 |
| 3-5 mo, cm | 1.6 | 2.5 | 3.4 | 4.3 | 5.2 | 6.1 | 7 |
| 4-6 mo, cm | 1 | 1.9 | 2.7 | 3.6 | 4.5 | 5.3 | 6.2 |
| 5-7 mo, cm | 0.7 | 1.5 | 2.4 | 3.2 | 4 | 4.8 | 5.7 |
| 6-8 mo, cm | 0.5 | 1.3 | 2.2 | 3 | 3.8 | 4.7 | 5.5 |
| 7-9 mo, cm | 0.4 | 1.2 | 2.1 | 2.9 | 3.7 | 4.5 | 5.4 |
| 8-10 mo, cm | 0.4 | 1.1 | 1.9 | 2.7 | 3.5 | 4.3 | 5.2 |
| 9-11 mo, cm | 0.3 | 1.1 | 1.8 | 2.6 | 3.4 | 4.2 | 5 |
| 10-12 mo, cm | 0.2 | 1 | 1.8 | 2.5 | 3.3 | 4.1 | 4.9 |
| Boys |  |  |  |  |  |  |  |
| 0-2 mo, cm | 5.1 | 6.2 | 7.3 | 8.5 | 9.6 | 10.8 | 11.9 |
| 1-3 mo, cm | 4.1 | 5.0 | 6.0 | 7.0 | 8.0 | 9.0 | 10.0 |
| 2-4 mo, cm | 2.7 | 3.7 | 4.6 | 5.6 | 6.5 | 7.5 | 8.5 |
| 3-5 mo, cm | 1.7 | 2.6 | 3.6 | 4.5 | 5.4 | 6.4 | 7.4 |
| 4-6 mo, cm | 1.1 | 1.9 | 2.8 | 3.7 | 4.6 | 5.6 | 6.5 |
| 5-7 mo, cm | 0.7 | 1.5 | 2.4 | 3.2 | 4.1 | 5.0 | 5.9 |
| 6-8 mo, cm | 0.5 | 1.3 | 2.1 | 3.0 | 3.8 | 4.7 | 5.5 |
| 7-9 mo, cm | 0.4 | 1.2 | 2.0 | 2.8 | 3.6 | 4.5 | 5.4 |
| 8-10 mo, cm | 0.3 | 1.1 | 1.9 | 2.7 | 3.5 | 4.4 | 5.2 |
| 9-11 mo, cm | 0.2 | 1.0 | 1.8 | 2.6 | 3.4 | 4.2 | 5.1 |
| 10-12 mo, cm | 0.2 | 0.9 | 1.7 | 2.5 | 3.3 | 4.1 | 4.9 |

# **Supplementary Table 3**: GMP guidance and criteria^a^

|  | WHO (12,13) | UNICEF (14) | Guatemala (15) | Honduras (16,17) | Ghana (18,19) | Burkina Faso (20) | Senegal (21) | Ethiopia (22–24) | India (25,26) | WHO (12,13) |
| --- | --- | --- | --- | --- | --- | --- | --- | --- | --- | --- |
| Frequency | Weight | - 0-24 mo: monthly - 24-60 mo: 3-monthly | - -12 mo: monthly - 12-24 mo: 3-monthly - 24-60 mo: not defined | - 0-24 mo: monthly - 24-36 mo: 3-monthly - 36-60 mo: 6-monthly | - 0-24 mo: monthly - 24-60 mo if sick: monthly | - 0-12 mo: monthly - 24-36 mo: 3-monthly - 36-60 mo: 6-monthly | - 0-24 mo: monthly - 24-36 mo: 2-monthly - 36-60 mo: 3-monthly | - 0-24 mo: monthly - 24-60 mo: not defined | - 0-24 mo: monthly - 24-60 mo: none | - mo: weekly - 1-36 mo: monthly - 36-60 mo: 3-monthly |
|  | Length | - 0-24 mo (length): 3-monthly - 24-60 mo (height): 3-monthly | Not defined | *Idem* | Not defined | - 0-24 mo 3-monthly - 24-60 mo: 6-monthly | *Idem* | *Idem* | Not defined | - 0-36 mo: 3-monthly - 36-60 mo: not defined |
| Criteria for single measurements | Weight | - WAZ<-2 - WLZ<-2 - BMIZ<-2 | Not defined | - WAZ < -2 - WLZ < -2 | Not defined | WAZ <-2 | - WAZ <-2 - WLZ < -3 | - WAZ<-2 | - WAZ < -2 | - WAZ<-2 - WLZ < -3 |
|  | Length | - LAZ/HAZ<-2 | Not defined | - LAZ/HAZ < -2 | Not defined | Not defined | Not defined | Not defined | Not defined | Not defined |
| Criteria for multiple measurements | Weight | - “Growth line crosses z-score line”, “trending away from median” - “Sharp incline or decline in growth line” - “Growth line remains flat” | “A line stays flat or goes down” | Direction of the trend of the growth indicators curve that is horizontal or below other points | Observed weight gain between measurements is < 5th percentile of expected weight gain relative to population standards^b^ | - “Incline or decline of the curve” - No weight gain between 2 weighing points | Weight loss or no weight gain between 2 weighing points | - “Growth curve is > 2 or <-2” - Weight loss or no weight gain between 2 weighing points | - Distance between a measurement and the WAZ curve above that point is larger than this distance for a previous month’s measurement - Weight loss or no weight gain between 2 weighing points | - No gain in weight for 2-3 mo - “Growth curve is > 3 or <-3” - “Any sharp incline or decline in the curve” |
|  | Length | *Idem* | Not defined | *Idem* | Not defined | Not defined | Not defined | Not defined | Not defined | Not defined |
| Number of observations needed to establish trend |  | Not defined | Not defined | Not defined | 2 weighing points | - Number of observations needed to constitute a curve not defined - 2 weighing points needed to estimate lack of weight gain | 2 weighing points | - Number of observations needed to constitute a curve not defined - 2 weighing points needed to estimate lack of weight gain | 2 weighing points needed to estimate lack of weight gain | - Number of observations needed to constitute a curve not defined - “2-3” weighing points needed to estimate lack of weight gain |

^a^ Criteria that are not based on weight or length used for wasting detection were not included in the table. Additionally, criteria for overweight and obesity (in terms of BMIZ or WAZ) were not included here.

^b^ Calculated as: $(weight (g) at timepoint 2-weight at timepoint 1)/(Age \left( days \right)at timepoint 2-age at timepoint 1)$. The midpoint between age at timepoint 1 and 2 is then used to identify the expected weight gain on the table for comparison against the 5^th^ percentile of the authors’ population-specific standards.

# **Supplementary Table 4**: Root mean square error (RMSE) of regression models of anthropometric indices at 18 and 24 months on anthropometric indices during infancy in Brazil, Peru, South Africa, Mali, Nepal, Tanzania, Bangladesh-Mirpur, India, Bangladesh - Matlab, and Burkina Faso.

The values in the table show the minimum and maximum RMSE across the 10 countries.

Abbreviations: WAZ = weight-for-age z-score, LAZ = length-for-age z-score, mo = months, kg = kilograms, cm = centimeters

|  | **WAZ** | | **LAZ** | |
| --- | --- | --- | --- | --- |
|  | **18 mo** | **24 mo** | **18 mo** | **24 mo** |
| WAZ at 3 mo | 0.689-1.022 | 0.680-1.006 | 0.771-1.050 | 0.747-0.991 |
| WAZ at 6 mo | 0.525-0.839 | 0.554-0.874 | 0.714-1.029 | 0.698-0.968 |
| WAZ at 12 mo | 0.400-0.567 | 0.415-0.635 | 0.642-1.000 | 0.620-0.913 |
| Weight at 3 mo, kg | 0.800-1.196 | 0.804-1.144 | 0.698-1.033 | 0.720-0.988 |
| Weight at 6 mo, kg | 0.692-1.148 | 0.692-1.105 | 0.549-0.905 | 0.584-0.896 |
| Weight at 12 mo, kg | 0.563-1.086 | 0.569-1.013 | 0.379-0.766 | 0.440-0.778 |
| LAZ at 3 mo | 0.722-1.051 | 0.704-1.028 | 0.780-1.085 | 0.788-1.024 |
| LAZ at 6 mo | 0.594-0.876 | 0.605-0.906 | 0.678-1.062 | 0.715-1.000 |
| LAZ at 12 mo | 0.475-0.723 | 0.538-0.735 | 0.526-1.029 | 0.541-0.945 |
| Length at 3 mo, cm | 0.817-1.200 | 0.806-1.148 | 0.741-1.057 | 0.750-1.017 |
| Length at 6 mo, cm | 0.751-1.159 | 0.742-1.119 | 0.648-0.965 | 0.637-0.956 |
| Length at 12 mo, cm | 0.681-1.095 | 0.710-1.024 | 0.524-0.679 | 0.514-0.810 |

# **Supplementary Table 5**: Root mean square error (RMSE) of regression models of anthropometric indices at 18 and 24 months on growth velocity during infancy in Brazil, Peru, South Africa, Mali, Nepal, Tanzania, Bangladesh-Mirpur, India, Bangladesh - Matlab, and Burkina Faso.

The values in the table show the minimum and maximum RMSE across the 10 countries.

Abbreviations: WAZ = weight-for-age z-score, LAZ = length-for-age z-score, kg = kilograms, mo = months, cm = centimeter

|  | **WAZ** | | **LAZ** | |
| --- | --- | --- | --- | --- |
|  | **18 mo** | **24 mo** | **18 mo** | **24 mo** |
| **Weight velocity (kg/mo)** |  |  |  |  |
| **1-mo increments** |  |  |  |  |
| Between 0 and 1 mo | 0.856-1.281 | 0.853-1.217 | 0.883-1.183 | 0.864-1.085 |
| Between 1 and 2 mo | 0.846-1.265 | 0.812-1.186 | 0.874-1.168 | 0.855-1.079 |
| Between 2 and 3 mo | 0.815-1.213 | 0.784-1.168 | 0.886-1.166 | 0.860-1.064 |
| Between 3 and 4 mo | 0.880-1.175 | 0.859-1.116 | 0.876-1.166 | 0.852-1.067 |
| Between 4 and 5 mo | 0.857-1.261 | 0.841-1.208 | 0.870-1.164 | 0.844-1.073 |
| Between 5 and 6 mo | 0.887-1.271 | 0.858-1.206 | 0.874-1.184 | 0.854-1.087 |
| Between 6 and 7 mo | 0.883-1.202 | 0.859-1.142 | 0.889-1.160 | 0.872-1.065 |
| Between 7 and 8 mo | 0.878-1.217 | 0.860-1.151 | 0.886-1.151 | 0.874-1.062 |
| Between 8 and 9 mo | 0.893-1.282 | 0.856-1.217 | 0.869-1.184 | 0.839-1.087 |
| Between 9 and 10 mo | 0.888-1.233 | 0.849-1.135 | 0.871-1.158 | 0.858-1.044 |
| Between 10 and 11 mo | 0.886-1.211 | 0.855-1.149 | 0.886-1.175 | 0.864-1.073 |
| Between 11 and 12 mo | 0.889-1.245 | 0.858-1.167 | 0.895-1.192 | 0.869-1.088 |
| **2-mo increments** |  |  |  |  |
| Between 0 and 2 mo | 0.841-1.253 | 0.816-1.195 | 0.861-1.166 | 0.845-1.084 |
| Between 1 and 3 mo | 0.782-1.169 | 0.761-1.105 | 0.869-1.138 | 0.837-1.047 |
| Between 2 and 4 mo | 0.826-1.083 | 0.799-1.056 | 0.874-1.145 | 0.839-1.040 |
| Between 3 and 5 mo | 0.835-1.122 | 0.817-1.089 | 0.850-1.134 | 0.823-1.070 |
| Between 4 and 6 mo | 0.834-1.243 | 0.807-1.192 | 0.853-1.172 | 0.843-1.077 |
| Between 5 and 7 mo | 0.875-1.188 | 0.845-1.130 | 0.857-1.170 | 0.852-1.071 |
| Between 6 and 8 mo | 0.877-1.130 | 0.857-1.070 | 0.876-1.126 | 0.861-1.055 |
| Between 7 and 9 mo | 0.869-1.223 | 0.853-1.171 | 0.859-1.161 | 0.838-1.062 |
| Between 8 and 10 mo | 0.891-1.240 | 0.847-1.164 | 0.865-1.167 | 0.832-1.183 |
| Between 9 and 11 mo | 0.880-1.161 | 0.846-1.070 | 0.875-1.150 | 0.865-1.065 |
| Between 10 and 12 mo | 0.879-1.157 | 0.850-1.087 | 0.878-1.171 | 0.858-1.072 |
| **3-mo increments** |  |  |  |  |
| Between 0 and 3 mo | 0.779-1.158 | 0.763-1.126 | 0.855-1.137 | 0.827-1.061 |
| Between 1 and 4 mo | 0.790-1.038 | 0.680-0.989 | 0.855-1.118 | 0.822-1.026 |
| Between 2 and 5 mo | 0.736-1.029 | 0.736-1.024 | 0.836-1.116 | 0.814-1.014 |
| Between 3 and 6 mo | 0.815-1.129 | 0.786-1.089 | 0.841-1.152 | 0.828-1.065 |
| Between 4 and 7 mo | 0.809-1.151 | 0.800-1.118 | 0.845-1.240 | 0.841-1.113 |
| Between 5 and 8 mo | 0.848-1.177 | 0.817-1.085 | 0.846-1.205 | 0.844-1.156 |
| Between 6 and 9 mo | 0.867-1.131 | 0.846-1.089 | 0.850-1.132 | 0.828-1.045 |
| Between 7 and 10 mo | 0.804-1.155 | 0.721-1.074 | 0.856-1.129 | 0.829-1.017 |
| Between 8 and 11 mo | 0.881-1.174 | 0.778-1.101 | 0.866-1.159 | 0.834-1.059 |
| Between 9 and 12 mo | 0.872-1.113 | 0.839-1.029 | 0.870-1.151 | 0.851-1.045 |
| **Length velocity, cm/mo** |  |  |  |  |
| **1-mo increments** |  |  |  |  |
| Between 0 and 1 mo | 0.850-1.182 | 0.846-1.129 | 0.866-1.185 | 0.849-1.090 |
| Between 1 and 2 mo | 0.846-1.265 | 0.812-1.186 | 0.874-1.168 | 0.855-1.079 |
| Between 2 and 3 mo | 0.815-1.213 | 0.784-1.168 | 0.886-1.166 | 0.860-1.064 |
| Between 3 and 4 mo | 0.880-1.175 | 0.859-1.116 | 0.876-1.166 | 0.852-1.067 |
| Between 4 and 5 mo | 0.857-1.261 | 0.841-1.208 | 0.870-1.164 | 0.844-1.073 |
| Between 5 and 6 mo | 0.891-1.271 | 0.858-1.206 | 0.874-1.184 | 0.854-1.087 |
| Between 6 and 7 mo | 0.887-1.202 | 0.860-1.142 | 0.889-1.160 | 0.872-1.065 |
| Between 7 and 8 mo | 0.878-1.214 | 0.858-1.146 | 0.886-1.154 | 0.874-1.062 |
| Between 8 and 9 mo | 0.881-1.285 | 0.857-1.219 | 0.869-1.187 | 0.839-1.090 |
| Between 9 and 10 mo | 0.894-1.233 | 0.856-1.135 | 0.871-1.158 | 0.858-1.045 |
| Between 10 and 11 mo | 0.900-1.211 | 0.860-1.149 | 0.886-1.175 | 0.864-1.073 |
| Between 11 and 12 mo | 0.894-1.245 | 0.861-1.167 | 0.896-1.192 | 0.869-1.088 |
| **2-mo increments** |  |  |  |  |
| Between 0 and 2 mo | 0.819-1.156 | 0.805-1.107 | 0.853-1.162 | 0.837-1.085 |
| Between 1 and 3 mo | 0.782-1.169 | 0.761-1.105 | 0.869-1.138 | 0.837-1.047 |
| Between 2 and 4 mo | 0.826-1.083 | 0.799-1.056 | 0.874-1.145 | 0.839-1.067 |
| Between 3 and 5 mo | 0.835-1.133 | 0.817-1.129 | 0.850-1.134 | 0.823-1.087 |
| Between 4 and 6 mo | 0.834-1.243 | 0.807-1.192 | 0.853-1.172 | 0.843-1.077 |
| Between 5 and 7 mo | 0.877-1.188 | 0.845-1.230 | 0.857-1.170 | 0.852-1.071 |
| Between 6 and 8 mo | 0.879-1.128 | 0.857-1.068 | 0.876-1.129 | 0.861-1.059 |
| Between 7 and 9 mo | 0.869-1.223 | 0.852-1.171 | 0.859-1.161 | 0.838-1.062 |
| Between 8 and 10 mo | 0.893-1.242 | 0.857-1.165 | 0.865-1.171 | 0.832-1.185 |
| Between 9 and 11 mo | 0.889-1.161 | 0.860-1.070 | 0.875-1.150 | 0.865-1.065 |
| Between 10 and 12 mo | 0.894-1.157 | 0.856-1.087 | 0.880-1.171 | 0.858-1.065 |
| **3-mo increments** |  |  |  |  |
| Between 0 and 3 mo | 0.756-1.078 | 0.749-1.054 | 0.838-1.135 | 0.818-1.060 |
| Between 1 and 4 mo | 0.790-1.038 | 0.712-0.989 | 0.855-1.118 | 0.822-1.026 |
| Between 2 and 5 mo | 0.736-1.029 | 0.736-1.024 | 0.836-1.116 | 0.811-1.014 |
| Between 3 and 6 mo | 0.815-1.129 | 0.786-1.089 | 0.841-1.152 | 0.828-1.066 |
| Between 4 and 7 mo | 0.809-1.151 | 0.800-1.118 | 0.845-1.258 | 0.841-1.113 |
| Between 5 and 8 mo | 0.849-1.112 | 0.817-1.082 | 0.846-1.228 | 0.844-1.163 |
| Between 6 and 9 mo | 0.868-1.164 | 0.846-1.089 | 0.850-1.132 | 0.828-1.045 |
| Between 7 and 10 mo | 0.865-1.155 | 0.854-1.074 | 0.856-1.129 | 0.829-1.028 |
| Between 8 and 11 mo | 0.872-1.176 | 0.685-1.102 | 0.866-1.162 | 0.766-1.059 |
| Between 9 and 12 mo | 0.887-1.113 | 0.857-1.116 | 0.870-1.151 | 0.851-1.045 |

# **Supplementary Table 6:** Predictive accuracy of GMP criteria for predicting subsequent inadequate growth in Brazil, Peru, South Africa, Mali, Nepal, Tanzania, Bangladesh-Mirpur, India, Bangladesh - Matlab, and Burkina Faso.

The values in the table show the minimum and maximum values across the 10 countries. Youden’s index is defined as sensitivity+specificity-1.

Abbreviations: mo = month(s); WAZ = weight-for-age z-score; LAZ = length-for-age z-score; LAD = length-for-age difference

|  | **Sensitivity, %** | **Specificity, %** | **Youden’s index** | **Positive predictive value, %** | **Negative predictive value, %** |
| --- | --- | --- | --- | --- | --- |
| **Underweight at 18 mo** |  |  |  |  |  |
| WAZ < -2 at any point before 12 mo of age | 66.7-95.2 | 69.7-94.7 | 0.52-0.83 | 18.2-67.5 | 88.6-99.4 |
| Loss in WAZ over a 1-mo period | 97.5-100.0 | 0.0-3.7 | 0.00-0.01 | 1.7-34.5 | 0.0-100.0 |
| Loss in WAZ over a 2-mo period | 68.5-100.0 | 5.3-31.9 | 0.00-0.14 | 2.0-35.7 | 69.2-100.0 |
| Loss in WAZ over a 3-mo period | 23.0-100.0 | 32.3-71.1 | 0.00-0.53 | 3.6-42.3 | 67.8-100.0 |
| Lack of weight over a 1-mo period | 68.4-100.0 | 5.4-36.3 | 0.00-0.36 | 2.7-36.5 | 70.6-100.0 |
| Lack of weight over a 2-mo period | 9.1-45.9 | 69.3-94.2 | 0.00-0.28 | 9.1-38.1 | 66.4-98.8 |
| Lack of weight over a 3-mo period | 0.0-33.3 | 94.5-100.0 | 0.00-0.33 | 0.0-100.0 | 65.9-98.8 |
| Loss in LAZ over a 1-mo period | 97.5-100.0 | 0.0-2.5 | 0.00-0.00 | 1.7-34.2 | 0.0-100.0 |
| Loss in LAZ over a 2-mo period | 74.3-100.0 | 3.1-27.5 | 0.00-0.13 | 1.8-34.9 | 67.3-100.0 |
| Loss in LAZ over a 3-mo period | 33.3-71.4 | 28.1-68.6 | 0.00-0.14 | 1.0-37.7 | 66.7-97.3 |
| Lack of length over a 1-mo period | 14.3-91.9 | 3.8-87.3 | 0.00-0.31 | 3.2-42.4 | 64.6-99.1 |
| Lack of length over a 2-mo period | 0.0-27.0 | 80.0-99.5 | 0.00-0.07 | 0.0-50.0 | 66.0-98.2 |
| Lack of length over a 3-mo period | 0.0-0.2 | 98.4-100.0 | 0.00-0.00 | 0.0-100.0 | 65.8-98.3 |
| **Underweight at 24 mo** |  |  |  |  |  |
| WAZ < -2 at any point before 12 mo of age | 61.2-90.0 | 68.5-93.8 | 0.44-0.71 | 16.7-69.4 | 86.4-99.3 |
| Loss in WAZ over a 1-mo period | 97.3-100.0 | 0.0-3.4 | 0.00-0.01 | 1.8-37.9 | 0.0-100.0 |
| Loss in WAZ over a 2-mo period | 65.9-100.0 | 7.3-32.1 | 0.00-0.14 | 2.1-38.7 | 64.6-100.0 |
| Loss in WAZ over a 3-mo period | 23.3-100.0 | 39.3-72.2 | 0.00-0.51 | 3.7-45.2 | 64.5-100.0 |
| Lack of weight over a 1-mo period | 73.3-100.0 | 6.1-37.0 | 0.04-0.37 | 2.9-39.7 | 69.6-100.0 |
| Lack of weight over a 2-mo period | 10.3-44.9 | 71.3-95.1 | 0.03-0.28 | 11.1-47.1 | 63.7-98.7 |
| Lack of weight over a 3-mo period | 0.0-33.3 | 97.6-100.0 | 0.00-0.33 | 0.0-100.0 | 62.5-98.8 |
| Loss in LAZ over a 1-mo period | 96.6-100.0 | 0.0-2.1 | 0.00-0.00 | 1.8-37.4 | 0.0-50.0 |
| Loss in LAZ over a 2-mo period | 72.8-100.0 | 2.8-27.2 | 0.00-0.11 | 1.9-37.7 | 62.3-100.0 |
| Loss in LAZ over a 3-mo period | 33.3-69.0 | 26.4-67.9 | 0.00-0.13 | 1.0-39.9 | 63.4-97.1 |
| Lack of length over a 1-mo period | 0.0-91.8 | 3.7-87.1 | 0.00-0.31 | 0.0-41.9 | 60.0-99.0 |
| Lack of length over a 2-mo period | 0.0-26.5 | 81.1-99.3 | 0.00-0.09 | 0.0-34.0 | 62.2-98.1 |
| Lack of length over a 3-mo period | 0.0-0.2 | 98.2-100.0 | 0.00-0.00 | 0.0-100.0 | 62.3-98.2 |
| **Stunting at 18 mo** |  |  |  |  |  |
| WAZ < -2 at any point before 12 mo of age | 16.7-68.6 | 64.8-94.1 | 0.11-0.48 | 9.1-91.4 | 36.9-97.0 |
| Loss in WAZ over a 1-mo period | 97.2-100.0 | 0.0-3.8 | 0.00-0.01 | 3.4-70.6 | 0.0-100.0 |
| Loss in WAZ over a 2-mo period | 69.8-96.2 | 4.9-31.8 | 0.00-0.04 | 3.3-70.7 | 30.8-96.0 |
| Loss in WAZ over a 3-mo period | 26.7-75.2 | 31.7-71.7 | 0.00-0.24 | 4.8-72.4 | 31.9-97.8 |
| Lack of weight over a 1-mo period | 50.0-97.2 | 5.4-36.1 | 0.00-0.18 | 2.7-71.5 | 45.5-95.2 |
| Lack of weight over a 2-mo period | 0.0-37.0 | 69.6-94.3 | 0.00-0.13 | 0.0-78.1 | 33.1-96.3 |
| Lack of weight over a 3-mo period | 0.0-8.8 | 95.0-100.0 | 0.00-0.04 | 0.0-100.0 | 29.9-96.6 |
| Loss in LAZ over a 1-mo period | 97.3-100.0 | 0.0-2.3 | 0.00-0.00 | 3.4-70.6 | 0.0-50.0 |
| Loss in LAZ over a 2-mo period | 74.2-100.0 | 3.1-27.9 | 0.00-0.13 | 3.7-70.3 | 22.2-100.0 |
| Loss in LAZ over a 3-mo period | 33.3-78.8 | 27.9-68.3 | 0.00-0.22 | 2.0-70.4 | 29.0-94.5 |
| Lack of length over a 1-mo period | 18.2-94.8 | 3.1-87.8 | 0.00-0.15 | 3.2-70.2 | 20.0-96.4 |
| Lack of length over a 2-mo period | 0.0-21.4 | 78.1-100.0 | 0.00-0.07 | 0.0-100.0 | 29.2-96.5 |
| Lack of length over a 3-mo period | 0.0-0.6 | 96.9-100.0 | 0.00-0.00 | 0.0-100.0 | 28.8-96.6 |
| **Stunting at 24 mo** |  |  |  |  |  |
| WAZ < -2 at any point before 12 mo of age | 16.7-64.4 | 61.1-93.1 | 0.10-0.44 | 8.3-91.5 | 37.5-96.7 |
| Loss in WAZ over a 1-mo period | 97.6-100.0 | 0.0-3.9 | 0.00-0.01 | 3.6-70.6 | 0.0-100.0 |
| Loss in WAZ over a 2-mo period | 68.8-100.0 | 4.8-33.6 | 0.00-0.15 | 4.2-70.4 | 25.0-100.0 |
| Loss in WAZ over a 3-mo period | 30.1-83.3 | 42.9-74.1 | 0.04-0.35 | 6.1-72.7 | 32.2-98.8 |
| Lack of weight over a 1-mo period | 71.3-94.6 | 4.8-37.1 | 0.00-0.20 | 4.8-70.5 | 27.3-98.3 |
| Lack of weight over a 2-mo period | 0.0-35.6 | 75.8-94.3 | 0.00-0.11 | 0.0-77.9 | 32.9-96.2 |
| Lack of weight over a 3-mo period | 0.0-4.7 | 98.4-100.0 | 0.00-0.03 | 0.0-100.0 | 30.0-96.3 |
| Loss in LAZ over a 1-mo period | 97.2-100.0 | 0.0-2.3 | 0.00-0.00 | 3.6-70.6 | 0.0-44.9 |
| Loss in LAZ over a 2-mo period | 74.2-100.0 | 1.6-28.7 | 0.00-0.09 | 3.9-70.0 | 12.5-100.0 |
| Loss in LAZ over a 3-mo period | 35.6-78.8 | 31.0-69.3 | 0.03-0.19 | 4.2-75.2 | 36.0-97.1 |
| Lack of length over a 1-mo period | 15.6-94.6 | 3.2-89.9 | 0.00-0.32 | 6.7-70.1 | 20.0-98.1 |
| Lack of length over a 2-mo period | 0.0-22.8 | 83.9-100.0 | 0.00-0.07 | 0.0-100.0 | 31.1-96.3 |
| Lack of length over a 3-mo period | 0.0-1.3 | 98.4-100.0 | 0.00-0.00 | 0.0-100.0 | 29.3-96.3 |
| **Loss in WAZ between 12 and 18 mo** |  |  |  |  |  |
| WAZ < -2 at any point before 12 mo of age | 5.4-45.1 | 42.7-92.1 | 0.00-0.03 | 31.0-77.8 | 21.1-44.9 |
| Loss in WAZ over a 1-mo period | 97.3-100.0 | 0.0-2.6 | 0.00-0.00 | 49.7-75.5 | 0.0-50.0 |
| Loss in WAZ over a 2-mo period | 69.3-95.7 | 2.9-30.8 | 0.00-0.05 | 49.9-75.1 | 20.0-53.8 |
| Loss in WAZ over a 3-mo period | 26.2-64.3 | 29.7-70.1 | 0.00-0.05 | 47.5-75.8 | 23.5-47.5 |
| Lack of weight over a 1-mo period | 64.0-94.0 | 1.3-34.9 | 0.00-0.00 | 49.2-74.2 | 10.0-43.2 |
| Lack of weight over a 2-mo period | 5.4-35.5 | 65.5-92.1 | 0.00-0.07 | 34.1-72.2 | 24.1-48.3 |
| Lack of weight over a 3-mo period | 0.0-4.9 | 93.1-100.0 | 0.00-0.01 | 0.0-100.0 | 24.6-49.7 |
| Loss in LAZ over a 1-mo period | 98.1-100.0 | 0.0-1.8 | 0.00-0.00 | 49.8-75.5 | 0.0-100.0 |
| Loss in LAZ over a 2-mo period | 75.4-97.5 | 1.9-27.4 | 0.00-0.10 | 49.3-75.2 | 16.7-75.0 |
| Loss in LAZ over a 3-mo period | 34.6-68.8 | 23.1-66.7 | 0.00-0.09 | 47.8-77.1 | 19.4-49.4 |
| Lack of length over a 1-mo period | 13.4-95.0 | 3.9-85.2 | 0.00-0.09 | 48.8-74.2 | 23.5-53.2 |
| Lack of length over a 2-mo period | 0.6-19.1 | 76.3-100.0 | 0.00-0.02 | 33.3-100.0 | 24.3-50.1 |
| Lack of length over a 3-mo period | 0.0-1.4 | 98.7-100.0 | 0.00-0.01 | 0.0-100.0 | 24.5-50.3 |
| **Loss in WAZ between 18 and 24 mo** |  |  |  |  |  |
| WAZ < -2 at any point before 12 mo of age | 5.8-48.7 | 47.7-90.2 | 0.00-0.00 | 29.4-63.6 | 24.5-59.5 |
| Loss in WAZ over a 1-mo period | 97.0-100.0 | 0.0-2.5 | 0.00-0.00 | 37.4-74.7 | 0.0-83.3 |
| Loss in WAZ over a 2-mo period | 69.6-95.8 | 4.6-32.5 | 0.00-0.03 | 38.5-75.0 | 27.3-65.3 |
| Loss in WAZ over a 3-mo period | 31.6-62.2 | 31.6-74.4 | 0.00-0.06 | 42.3-75.6 | 26.2-64.7 |
| Lack of weight over a 1-mo period | 62.0-96.9 | 7.6-29.3 | 0.00-0.04 | 37.0-72.1 | 20.7-60.9 |
| Lack of weight over a 2-mo period | 5.8-32.8 | 68.4-96.3 | 0.00-0.11 | 34.9-81.0 | 25.5-62.2 |
| Lack of weight over a 3-mo period | 0.0-4.7 | 98.7-100.0 | 0.00-0.03 | 0.0-100.0 | 25.5-62.9 |
| Loss in LAZ over a 1-mo period | 97.5-100.0 | 0.0-1.8 | 0.00-0.00 | 37.3-74.7 | 0.0-32.1 |
| Loss in LAZ over a 2-mo period | 72.4-98.3 | 0.0-22.9 | 0.00-0.04 | 37.8-74.8 | 0.0-71.4 |
| Loss in LAZ over a 3-mo period | 32.2-70.9 | 27.8-63.0 | 0.00-0.09 | 39.8-77.7 | 29.4-65.2 |
| Lack of length over a 1-mo period | 15.4-95.3 | 5.1-85.7 | 0.00-0.06 | 35.3-73.3 | 24.5-61.8 |
| Lack of length over a 2-mo period | 0.0-23.4 | 82.3-100.0 | 0.00-0.06 | 0.0-100.0 | 25.9-62.5 |
| Lack of length over a 3-mo period | 0.0-1.6 | 98.2-100.0 | 0.00-0.01 | 0.0-100.0 | 25.5-62.7 |
| **Loss in WAZ between 12 and 24 mo** |  |  |  |  |  |
| WAZ < -2 at any point before 12 mo of age | 6.5-42.9 | 37.9-90.0 | 0.00-0.00 | 29.0-77.5 | 15.2-46.2 |
| Loss in WAZ over a 1-mo period | 97.3-100.0 | 0.0-1.8 | 0.00-0.00 | 46.6-82.0 | 0.0-66.7 |
| Loss in WAZ over a 2-mo period | 67.8-94.4 | 3.6-32.1 | 0.00-0.05 | 46.5-81.3 | 13.0-53.4 |
| Loss in WAZ over a 3-mo period | 26.3-58.5 | 30.6-72.0 | 0.00-0.00 | 45.0-79.3 | 14.3-52.9 |
| Lack of weight over a 1-mo period | 61.7-94.0 | 1.7-32.5 | 0.00-0.00 | 45.0-79.9 | 10.0-44.4 |
| Lack of weight over a 2-mo period | 4.8-32.2 | 66.1-92.5 | 0.00-0.01 | 29.3-70.6 | 16.9-52.5 |
| Lack of weight over a 3-mo period | 0.0-3.4 | 94.9-100.0 | 0.00-0.01 | 0.0-100.0 | 18.0-53.2 |
| Loss in LAZ over a 1-mo period | 97.8-100.0 | 0.0-1.4 | 0.00-0.00 | 46.5-82.0 | 0.0-18.0 |
| Loss in LAZ over a 2-mo period | 73.4-98.4 | 0.0-24.5 | 0.00-0.09 | 47.3-82.4 | 0.0-83.3 |
| Loss in LAZ over a 3-mo period | 33.4-71.1 | 26.5-63.9 | 0.00-0.07 | 48.2-83.1 | 20.0-55.2 |
| Lack of length over a 1-mo period | 13.8-94.6 | 3.4-89.2 | 0.00-0.05 | 44.1-86.2 | 18.8-52.4 |
| Lack of length over a 2-mo period | 0.6-18.1 | 74.6-100.0 | 0.00-0.03 | 40.0-100.0 | 18.1-53.4 |
| Lack of length over a 3-mo period | 0.0-1.3 | 98.3-100.0 | 0.00-0.01 | 0.0-100.0 | 18.0-53.5 |
| **Loss in LAZ between 12 and 18 mo** |  |  |  |  |  |
| WAZ < -2 at any point before 12 mo of age | 4.5-46.2 | 35.2-90.5 | 0.00-0.03 | 45.5-79.3 | 17.1-35.9 |
| Loss in WAZ over a 1-mo period | 96.9-100.0 | 0.0-1.7 | 0.00-0.00 | 64.0-79.1 | 0.0-33.3 |
| Loss in WAZ over a 2-mo period | 69.4-96.5 | 5.5-31.4 | 0.00-0.10 | 64.8-79.9 | 26.3-53.8 |
| Loss in WAZ over a 3-mo period | 28.4-67.2 | 32.7-73.4 | 0.00-0.15 | 65.1-81.6 | 24.4-39.6 |
| Lack of weight over a 1-mo period | 62.5-95.1 | 3.8-36.2 | 0.00-0.14 | 62.5-80.4 | 18.6-52.3 |
| Lack of weight over a 2-mo period | 7.1-34.6 | 65.6-98.2 | 0.00-0.09 | 61.6-94.1 | 21.2-36.6 |
| Lack of weight over a 3-mo period | 0.0-6.2 | 94.8-100.0 | 0.00-0.02 | 0.0-100.0 | 20.8-36.2 |
| Loss in LAZ over a 1-mo period | 97.6-100.0 | 0.0-0.7 | 0.00-0.00 | 64.0-79.2 | 0.0-12.0 |
| Loss in LAZ over a 2-mo period | 72.6-96.1 | 0.0-21.3 | 0.00-0.05 | 64.5-79.0 | 0.0-54.5 |
| Loss in LAZ over a 3-mo period | 32.3-65.6 | 15.8-61.6 | 0.00-0.09 | 61.0-77.8 | 14.5-41.1 |
| Lack of length over a 1-mo period | 14.6-94.4 | 1.9-86.0 | 0.00-0.08 | 53.5-81.0 | 10.0-33.0 |
| Lack of length over a 2-mo period | 0.0-17.9 | 69.2-100.0 | 0.00-0.01 | 0.0-100.0 | 20.9-36.3 |
| Lack of length over a 3-mo period | 0.0-0.6 | 96.2-100.0 | 0.00-0.00 | 0.0-33.3 | 20.8-35.6 |
| **Loss in LAZ between 18 and 24 mo** |  |  |  |  |  |
| WAZ < -2 at any point before 12 mo of age | 6.6-54.2 | 52.3-93.0 | 0.00-0.06 | 45.6-63.6 | 27.9-60.7 |
| Loss in WAZ over a 1-mo period | 97.8-100.0 | 0.0-3.6 | 0.00-0.01 | 42.5-69.6 | 0.0-100.0 |
| Loss in WAZ over a 2-mo period | 71.3-93.9 | 3.7-34.1 | 0.00-0.08 | 41.3-69.9 | 27.3-50.6 |
| Loss in WAZ over a 3-mo period | 28.4-64.3 | 34.8-72.9 | 0.00-0.07 | 42.3-66.4 | 26.4-57.1 |
| Lack of weight over a 1-mo period | 60.4-95.0 | 4.9-38.4 | 0.00-0.09 | 40.5-66.4 | 24.7-52.9 |
| Lack of weight over a 2-mo period | 4.7-35.0 | 69.5-95.9 | 0.00-0.05 | 42.9-83.3 | 31.9-57.6 |
| Lack of weight over a 3-mo period | 0.0-4.2 | 97.6-100.0 | 0.00-0.02 | 0.0-100.0 | 30.7-57.8 |
| Loss in LAZ over a 1-mo period | 98.1-100.0 | 0.0-2.7 | 0.00-0.01 | 42.5-69.3 | 0.0-49.1 |
| Loss in LAZ over a 2-mo period | 75.6-96.9 | 1.4-27.6 | 0.00-0.04 | 43.5-69.9 | 20.0-75.0 |
| Loss in LAZ over a 3-mo period | 33.8-71.3 | 28.8-65.8 | 0.00-0.09 | 42.6-70.8 | 32.0-57.8 |
| Lack of length over a 1-mo period | 14.7-95.0 | 4.9-84.6 | 0.00-0.06 | 41.0-61.5 | 28.9-56.6 |
| Lack of length over a 2-mo period | 0.0-23.3 | 80.5-100.0 | 0.00-0.04 | 0.0-100.0 | 30.8-57.8 |
| Lack of length over a 3-mo period | 0.0-1.7 | 98.0-100.0 | 0.00-0.01 | 0.0-100.0 | 30.7-57.5 |
| **Loss in LAZ between 12 and 24 mo** |  |  |  |  |  |
| WAZ < -2 at any point before 12 mo of age | 7.0-46.8 | 41.8-92.0 | 0.00-0.02 | 58.8-74.1 | 12.8-35.7 |
| Loss in WAZ over a 1-mo period | 97.6-100.0 | 0.0-2.8 | 0.00-0.03 | 62.7-84.4 | 0.0-100.0 |
| Loss in WAZ over a 2-mo period | 69.5-95.6 | 7.5-34.8 | 0.00-0.10 | 64.2-85.5 | 21.7-50.0 |
| Loss in WAZ over a 3-mo period | 28.5-66.2 | 37.8-75.0 | 0.00-0.17 | 65.7-83.8 | 15.6-38.4 |
| Lack of weight over a 1-mo period | 63.5-95.1 | 4.4-36.0 | 0.00-0.10 | 63.2-83.1 | 13.9-40.6 |
| Lack of weight over a 2-mo period | 5.2-33.3 | 68.9-96.4 | 0.00-0.04 | 60.3-88.9 | 16.3-36.9 |
| Lack of weight over a 3-mo period | 0.0-4.9 | 98.4-100.0 | 0.00-0.05 | 0.0-100.0 | 15.9-37.3 |
| Loss in LAZ over a 1-mo period | 97.9-100.0 | 0.0-1.3 | 0.00-0.00 | 62.7-84.1 | 0.0-16.3 |
| Loss in LAZ over a 2-mo period | 73.1-97.5 | 0.0-23.9 | 0.00-0.11 | 62.4-85.7 | 0.0-37.5 |
| Loss in LAZ over a 3-mo period | 33.2-67.6 | 18.2-63.3 | 0.00-0.10 | 61.5-86.2 | 15.3-36.1 |
| Lack of length over a 1-mo period | 13.0-93.8 | 0.0-81.4 | 0.00-0.09 | 55.2-82.1 | 0.0-34.0 |
| Lack of length over a 2-mo period | 0.0-17.9 | 71.1-100.0 | 0.00-0.01 | 0.0-100.0 | 16.1-37.2 |
| Lack of length over a 3-mo period | 0.0-0.6 | 95.6-100.0 | 0.00-0.00 | 0.0-50.0 | 15.9-37.3 |
| **Loss in LAD between 12 and 18 mo** |  |  |  |  |  |
| WAZ < -2 at any point before 12 mo of age | 5.5-50.7 | 50.0-92.4 | 0.00-0.10 | 54.5-91.5 | 9.0-37.2 |
| Loss in WAZ over a 1-mo period | 97.2-100.0 | 0.0-1.8 | 0.00-0.00 | 62.3-91.1 | 0.0-16.7 |
| Loss in WAZ over a 2-mo period | 70.0-95.2 | 4.7-34.2 | 0.00-0.10 | 64.0-91.0 | 7.7-48.0 |
| Loss in WAZ over a 3-mo period | 29.0-66.8 | 30.9-76.9 | 0.00-0.21 | 70.2-92.2 | 10.7-45.1 |
| Lack of weight over a 1-mo period | 61.5-95.7 | 4.2-44.1 | 0.00-0.14 | 59.8-90.5 | 7.5-36.4 |
| Lack of weight over a 2-mo period | 7.3-34.4 | 66.0-100.0 | 0.00-0.10 | 72.7-100.0 | 9.8-38.4 |
| Lack of weight over a 3-mo period | 0.0-5.9 | 94.2-100.0 | 0.00-0.02 | 0.0-100.0 | 8.9-37.9 |
| Loss in LAZ over a 1-mo period | 98.0-100.0 | 0.0-0.9 | 0.00-0.00 | 62.3-91.1 | 0.0-8.0 |
| Loss in LAZ over a 2-mo period | 73.5-96.6 | 0.0-21.4 | 0.00-0.04 | 63.4-90.6 | 0.0-54.5 |
| Loss in LAZ over a 3-mo period | 33.6-67.8 | 14.7-63.1 | 0.00-0.11 | 66.7-91.2 | 8.1-43.8 |
| Lack of length over a 1-mo period | 15.0-95.2 | 3.6-88.2 | 0.00-0.13 | 58.7-93.9 | 10.0-35.7 |
| Lack of length over a 2-mo period | 0.0-17.7 | 57.1-100.0 | 0.00-0.01 | 0.0-100.0 | 8.6-38.0 |
| Lack of length over a 3-mo period | 0.0-0.5 | 92.9-100.0 | 0.00-0.00 | 0.0-50.0 | 8.9-37.4 |
| **Loss in LAD between 18 and 24 mo** |  |  |  |  |  |
| WAZ < -2 at any point before 12 mo of age | 7.4-54.2 | 56.0-93.9 | 0.00-0.11 | 54.5-78.4 | 24.0-50.7 |
| Loss in WAZ over a 1-mo period | 97.5-100.0 | 0.0-3.6 | 0.00-0.02 | 49.7-76.3 | 0.0-100.0 |
| Loss in WAZ over a 2-mo period | 69.6-94.4 | 1.8-33.6 | 0.00-0.08 | 51.4-76.3 | 9.1-60.9 |
| Loss in WAZ over a 3-mo period | 27.9-67.9 | 39.3-72.6 | 0.00-0.15 | 54.9-77.6 | 25.5-55.6 |
| Lack of weight over a 1-mo period | 65.4-95.9 | 7.1-39.5 | 0.00-0.13 | 51.0-75.7 | 20.5-52.5 |
| Lack of weight over a 2-mo period | 3.7-36.3 | 75.0-96.7 | 0.00-0.11 | 33.3-88.9 | 25.0-49.4 |
| Lack of weight over a 3-mo period | 0.0-4.8 | 97.9-100.0 | 0.00-0.05 | 0.0-100.0 | 24.0-50.0 |
| Loss in LAZ over a 1-mo period | 98.0-100.0 | 0.0-2.8 | 0.00-0.01 | 49.7-76.0 | 0.0-30.9 |
| Loss in LAZ over a 2-mo period | 75.0-97.9 | 2.6-28.0 | 0.00-0.08 | 49.3-77.5 | 25.0-57.1 |
| Loss in LAZ over a 3-mo period | 34.3-73.2 | 29.7-66.9 | 0.00-0.09 | 52.6-78.5 | 25.1-54.4 |
| Lack of length over a 1-mo period | 16.4-95.2 | 5.4-88.3 | 0.00-0.05 | 40.0-77.4 | 23.5-44.7 |
| Lack of length over a 2-mo period | 0.0-23.3 | 82.1-100.0 | 0.00-0.05 | 0.0-100.0 | 24.1-50.9 |
| Lack of length over a 3-mo period | 0.0-1.4 | 97.8-100.0 | 0.00-0.01 | 0.0-100.0 | 24.0-50.6 |
| **Loss in LAD between 12 and 24 mo** |  |  |  |  |  |
| WAZ < -2 at any point before 12 mo of age | 8.3-50.8 | 53.8-94.2 | 0.00-0.25 | 66.7-96.6 | 7.4-42.5 |
| Loss in WAZ over a 1-mo period | 97.7-100.0 | 0.0-4.3 | 0.00-0.04 | 58.2-93.7 | 0.0-100.0 |
| Loss in WAZ over a 2-mo period | 68.2-95.5 | 7.7-34.6 | 0.02-0.18 | 60.6-93.8 | 8.3-56.5 |
| Loss in WAZ over a 3-mo period | 26.9-64.2 | 30.8-71.4 | 0.00-0.23 | 69.5-94.2 | 4.4-53.0 |
| Lack of weight over a 1-mo period | 63.5-95.9 | 15.4-42.5 | 0.00-0.15 | 58.1-94.4 | 8.8-41.7 |
| Lack of weight over a 2-mo period | 6.2-33.5 | 76.9-100.0 | 0.00-0.10 | 66.7-100.0 | 7.2-42.3 |
| Lack of weight over a 3-mo period | 0.0-4.1 | 97.7-100.0 | 0.00-0.04 | 0.0-100.0 | 6.5-42.1 |
| Loss in LAZ over a 1-mo period | 98.0-100.0 | 0.0-1.1 | 0.00-0.00 | 58.2-93.7 | 0.0-4.0 |
| Loss in LAZ over a 2-mo period | 73.5-97.3 | 0.0-22.7 | 0.00-0.14 | 59.1-93.5 | 0.0-54.5 |
| Loss in LAZ over a 3-mo period | 33.9-69.5 | 20.0-63.8 | 0.00-0.16 | 63.5-92.6 | 4.7-49.3 |
| Lack of length over a 1-mo period | 13.9-94.8 | 0.0-87.5 | 0.00-0.11 | 53.3-93.4 | 0.0-39.0 |
| Lack of length over a 2-mo period | 0.0-19.1 | 61.5-100.0 | 0.00-0.01 | 0.0-100.0 | 4.8-41.6 |
| Lack of length over a 3-mo period | 0.0-1.0 | 92.3-100.0 | 0.00-0.00 | 0.0-100.0 | 5.9-41.5 |

# **Supplementary Table 7:** Predictive accuracy of GMP criteria for predicting subsequent inadequate growth after excluding children with low weight-for-length z-score in Brazil, Peru, South Africa, Mali, Nepal, Tanzania, Bangladesh-Mirpur, India, Bangladesh - Matlab, and Burkina Faso.

The values in the table show the minimum and maximum values across the 10 countries. Youden’s index is defined as sensitivity+specificity-1.

Abbreviations: mo = month(s); WAZ = weight-for-age z-score; LAZ = length-for-age z-score; LAD = length-for-age difference

|  | **Sensitivity, %** | **Specificity, %** | **Youden’s index** | **Positive predictive value, %** | **Negative predictive value, %** |
| --- | --- | --- | --- | --- | --- |
| **Underweight at 18 mo** |  |  |  |  |  |
| WAZ < -2 at any point before 12 mo of age | 60.5-100.0 | 79.6-95.6 | 0.48-0.96 | 12.5-60.5 | 88.1-100.0 |
| Loss in WAZ over a 1-mo period | 96.0-100.0 | 0.0-4.3 | 0.00-0.01 | 0.6-23.6 | 0.0-100.0 |
| Loss in WAZ over a 2-mo period | 69.2-100.0 | 4.4-32.3 | 0.00-0.14 | 0.7-24.8 | 79.1-100.0 |
| Loss in WAZ over a 3-mo period | 20.8-100.0 | 30.8-70.9 | 0.00-0.53 | 1.3-29.9 | 77.5-100.0 |
| Lack of weight over a 1-mo period | 62.1-100.0 | 5.8-35.3 | 0.00-0.35 | 1.0-25.2 | 71.8-100.0 |
| Lack of weight over a 2-mo period | 0.0-51.7 | 68.8-93.7 | 0.00-0.21 | 0.0-23.1 | 75.9-99.3 |
| Lack of weight over a 3-mo period | 0.0-13.8 | 94.7-100.0 | 0.00-0.12 | 0.0-57.1 | 76.4-99.4 |
| Loss in LAZ over a 1-mo period | 96.6-100.0 | 0.0-2.8 | 0.00-0.00 | 0.6-23.4 | 0.0-100.0 |
| Loss in LAZ over a 2-mo period | 74.1-100.0 | 3.2-28.4 | 0.00-0.12 | 0.7-24.1 | 78.1-100.0 |
| Loss in LAZ over a 3-mo period | 0.0-70.0 | 29.0-69.0 | 0.00-0.15 | 0.0-26.2 | 73.5-98.5 |
| Lack of length over a 1-mo period | 10.0-100.0 | 4.0-86.8 | 0.00-0.64 | 1.7-25.2 | 74.7-100.0 |
| Lack of length over a 2-mo period | 0.0-27.6 | 79.8-99.5 | 0.00-0.07 | 0.0-50.0 | 76.4-99.4 |
| Lack of length over a 3-mo period | 0.0-0.0 | 98.3-100.0 | 0.00-0.00 | 0.0-0.0 | 76.5-99.4 |
| **Underweight at 24 mo** |  |  |  |  |  |
| WAZ < -2 at any point before 12 mo of age | 53.8-100.0 | 78.6-94.7 | 0.42-0.95 | 11.1-64.2 | 86.5-100.0 |
| Loss in WAZ over a 1-mo period | 95.9-100.0 | 0.0-3.9 | 0.00-0.01 | 0.7-27.6 | 0.0-100.0 |
| Loss in WAZ over a 2-mo period | 65.8-100.0 | 5.1-32.5 | 0.00-0.14 | 0.8-28.4 | 74.0-100.0 |
| Loss in WAZ over a 3-mo period | 17.7-100.0 | 38.8-71.7 | 0.00-0.51 | 1.4-34.5 | 73.6-100.0 |
| Lack of weight over a 1-mo period | 63.6-100.0 | 6.5-35.3 | 0.00-0.35 | 1.0-29.2 | 71.8-100.0 |
| Lack of weight over a 2-mo period | 0.0-50.0 | 70.6-94.7 | 0.00-0.21 | 0.0-38.5 | 73.1-99.3 |
| Lack of weight over a 3-mo period | 0.0-10.0 | 97.4-100.0 | 0.00-0.07 | 0.0-100.0 | 72.4-99.3 |
| Loss in LAZ over a 1-mo period | 95.3-100.0 | 0.0-2.3 | 0.00-0.00 | 0.7-27.2 | 0.0-56.7 |
| Loss in LAZ over a 2-mo period | 72.1-100.0 | 2.9-28.2 | 0.00-0.09 | 0.7-27.7 | 72.5-100.0 |
| Loss in LAZ over a 3-mo period | 0.0-69.2 | 27.1-68.2 | 0.00-0.19 | 0.0-28.0 | 70.9-98.4 |
| Lack of length over a 1-mo period | 0.0-100.0 | 3.9-86.0 | 0.00-0.63 | 0.0-30.8 | 66.7-100.0 |
| Lack of length over a 2-mo period | 0.0-27.5 | 81.0-99.1 | 0.00-0.09 | 0.0-50.0 | 72.2-99.3 |
| Lack of length over a 3-mo period | 0.0-0.0 | 98.0-100.0 | 0.00-0.00 | 0.0-0.0 | 72.3-99.3 |
| **Stunting at 18 mo** |  |  |  |  |  |
| WAZ < -2 at any point before 12 mo of age | 20.0-52.2 | 79.2-96.6 | 0.16-0.43 | 12.5-95.2 | 35.7-97.4 |
| Loss in WAZ over a 1-mo period | 96.3-100.0 | 0.0-4.6 | 0.00-0.01 | 3.1-70.9 | 0.0-100.0 |
| Loss in WAZ over a 2-mo period | 71.5-100.0 | 4.3-33.2 | 0.00-0.15 | 3.6-71.1 | 33.3-100.0 |
| Loss in WAZ over a 3-mo period | 26.6-80.0 | 30.0-71.5 | 0.00-0.34 | 5.3-73.5 | 32.6-98.8 |
| Lack of weight over a 1-mo period | 60.0-95.7 | 6.1-37.0 | 0.00-0.14 | 2.9-71.8 | 45.5-96.4 |
| Lack of weight over a 2-mo period | 0.0-39.0 | 70.7-94.4 | 0.00-0.15 | 0.0-79.7 | 33.8-96.7 |
| Lack of weight over a 3-mo period | 0.0-8.8 | 95.2-100.0 | 0.00-0.04 | 0.0-100.0 | 29.7-96.9 |
| Loss in LAZ over a 1-mo period | 96.8-100.0 | 0.0-2.7 | 0.00-0.00 | 3.1-70.9 | 0.0-54.0 |
| Loss in LAZ over a 2-mo period | 73.9-100.0 | 3.4-29.0 | 0.00-0.12 | 3.3-70.8 | 28.6-100.0 |
| Loss in LAZ over a 3-mo period | 33.2-77.4 | 29.5-68.7 | 0.00-0.18 | 2.2-70.5 | 28.7-95.6 |
| Lack of length over a 1-mo period | 13.7-95.0 | 3.4-87.1 | 0.00-0.11 | 3.4-70.5 | 22.2-97.1 |
| Lack of length over a 2-mo period | 0.0-22.0 | 79.3-100.0 | 0.00-0.03 | 0.0-100.0 | 29.5-96.8 |
| Lack of length over a 3-mo period | 0.0-0.7 | 96.6-100.0 | 0.00-0.00 | 0.0-33.3 | 28.6-96.9 |
| **Stunting at 24 mo** |  |  |  |  |  |
| WAZ < -2 at any point before 12 mo of age | 25.0-47.5 | 78.9-96.5 | 0.20-0.39 | 11.1-95.2 | 36.9-97.9 |
| Loss in WAZ over a 1-mo period | 96.8-100.0 | 0.0-4.5 | 0.00-0.01 | 2.6-70.2 | 0.0-100.0 |
| Loss in WAZ over a 2-mo period | 70.9-100.0 | 4.2-34.4 | 0.00-0.14 | 3.1-70.0 | 27.3-100.0 |
| Loss in WAZ over a 3-mo period | 29.9-75.0 | 41.4-73.7 | 0.04-0.27 | 4.1-72.6 | 32.9-98.7 |
| Lack of weight over a 1-mo period | 63.9-94.0 | 5.3-36.0 | 0.00-0.10 | 3.1-70.0 | 27.3-98.1 |
| Lack of weight over a 2-mo period | 0.0-38.1 | 77.2-94.6 | 0.00-0.15 | 0.0-79.7 | 34.6-97.2 |
| Lack of weight over a 3-mo period | 0.0-5.2 | 98.2-100.0 | 0.00-0.03 | 0.0-100.0 | 30.6-97.4 |
| Loss in LAZ over a 1-mo period | 96.8-100.0 | 0.0-2.7 | 0.00-0.00 | 2.6-70.2 | 0.0-49.2 |
| Loss in LAZ over a 2-mo period | 73.6-100.0 | 0.0-29.6 | 0.00-0.08 | 2.9-69.2 | 0.0-100.0 |
| Loss in LAZ over a 3-mo period | 33.9-85.7 | 31.5-69.8 | 0.03-0.24 | 3.4-74.8 | 36.2-98.4 |
| Lack of length over a 1-mo period | 13.7-94.8 | 3.5-88.6 | 0.00-0.13 | 3.6-69.8 | 22.2-97.9 |
| Lack of length over a 2-mo period | 0.0-23.9 | 86.0-100.0 | 0.00-0.10 | 0.0-100.0 | 32.5-97.3 |
| Lack of length over a 3-mo period | 0.0-1.5 | 98.2-100.0 | 0.00-0.00 | 0.0-66.7 | 29.8-97.3 |
| **Loss in WAZ between 12 and 18 mo** |  |  |  |  |  |
| WAZ < -2 at any point before 12 mo of age | 4.0-32.1 | 63.2-93.2 | 0.00-0.03 | 40.0-78.9 | 21.1-43.3 |
| Loss in WAZ over a 1-mo period | 96.8-100.0 | 0.0-3.7 | 0.00-0.00 | 54.4-75.2 | 0.0-50.0 |
| Loss in WAZ over a 2-mo period | 70.4-96.3 | 2.0-31.5 | 0.00-0.11 | 54.3-77.4 | 14.3-58.3 |
| Loss in WAZ over a 3-mo period | 26.9-65.9 | 26.8-70.5 | 0.00-0.10 | 51.8-77.6 | 24.7-46.5 |
| Lack of weight over a 1-mo period | 63.4-93.6 | 1.6-35.6 | 0.00-0.05 | 54.3-76.3 | 10.0-42.5 |
| Lack of weight over a 2-mo period | 5.0-35.8 | 68.0-95.6 | 0.00-0.04 | 41.7-84.6 | 24.1-44.1 |
| Lack of weight over a 3-mo period | 0.0-5.0 | 93.8-100.0 | 0.00-0.01 | 0.0-100.0 | 24.8-45.3 |
| Loss in LAZ over a 1-mo period | 97.8-100.0 | 0.0-1.7 | 0.00-0.00 | 54.5-75.2 | 0.0-100.0 |
| Loss in LAZ over a 2-mo period | 75.0-98.3 | 2.0-29.4 | 0.00-0.10 | 54.3-76.5 | 16.7-50.0 |
| Loss in LAZ over a 3-mo period | 33.7-69.6 | 24.5-68.7 | 0.00-0.06 | 53.2-75.7 | 21.1-44.1 |
| Lack of length over a 1-mo period | 13.0-94.8 | 3.2-86.8 | 0.00-0.12 | 47.7-75.0 | 21.9-50.0 |
| Lack of length over a 2-mo period | 0.0-19.4 | 76.2-100.0 | 0.00-0.03 | 0.0-100.0 | 24.6-45.5 |
| Lack of length over a 3-mo period | 0.0-1.5 | 98.4-100.0 | 0.00-0.01 | 0.0-100.0 | 24.8-45.6 |
| **Loss in WAZ between 18 and 24 mo** |  |  |  |  |  |
| WAZ < -2 at any point before 12 mo of age | 4.5-31.9 | 66.1-92.1 | 0.00-0.00 | 27.0-62.5 | 25.0-59.3 |
| Loss in WAZ over a 1-mo period | 96.3-100.0 | 0.0-3.3 | 0.00-0.01 | 38.4-74.3 | 0.0-83.3 |
| Loss in WAZ over a 2-mo period | 69.7-97.1 | 4.8-31.9 | 0.00-0.04 | 39.2-75.0 | 23.5-66.7 |
| Loss in WAZ over a 3-mo period | 29.9-58.0 | 31.5-73.4 | 0.00-0.03 | 41.1-75.7 | 27.0-62.9 |
| Lack of weight over a 1-mo period | 61.4-96.6 | 8.6-29.6 | 0.00-0.06 | 38.0-71.1 | 19.6-60.8 |
| Lack of weight over a 2-mo period | 5.5-35.0 | 68.6-94.7 | 0.00-0.06 | 34.6-76.9 | 25.7-61.2 |
| Lack of weight over a 3-mo period | 0.0-5.1 | 98.6-100.0 | 0.00-0.04 | 0.0-100.0 | 25.7-61.8 |
| Loss in LAZ over a 1-mo period | 97.2-100.0 | 0.0-2.2 | 0.00-0.00 | 38.2-74.3 | 0.0-31.9 |
| Loss in LAZ over a 2-mo period | 71.2-98.9 | 0.0-22.8 | 0.00-0.04 | 38.7-74.5 | 0.0-75.0 |
| Loss in LAZ over a 3-mo period | 31.8-72.1 | 27.4-65.1 | 0.00-0.11 | 41.1-77.6 | 30.2-64.5 |
| Lack of length over a 1-mo period | 13.7-95.7 | 5.7-88.9 | 0.00-0.07 | 36.7-73.2 | 25.0-61.1 |
| Lack of length over a 2-mo period | 0.0-23.9 | 82.9-100.0 | 0.00-0.07 | 0.0-100.0 | 26.2-61.6 |
| Lack of length over a 3-mo period | 0.0-1.7 | 97.6-100.0 | 0.00-0.01 | 0.0-100.0 | 25.9-61.8 |
| **Loss in WAZ between 12 and 24 mo** |  |  |  |  |  |
| WAZ < -2 at any point before 12 mo of age | 5.3-28.0 | 58.5-91.7 | 0.00-0.00 | 34.3-78.9 | 13.6-45.6 |
| Loss in WAZ over a 1-mo period | 96.9-100.0 | 0.0-2.5 | 0.00-0.00 | 50.9-84.5 | 0.0-66.7 |
| Loss in WAZ over a 2-mo period | 68.1-95.1 | 2.1-31.4 | 0.00-0.06 | 50.6-83.7 | 7.7-48.8 |
| Loss in WAZ over a 3-mo period | 25.7-59.3 | 29.5-71.0 | 0.00-0.00 | 47.8-82.4 | 12.7-48.1 |
| Lack of weight over a 1-mo period | 59.2-93.7 | 2.2-30.6 | 0.00-0.00 | 49.9-83.0 | 10.0-44.6 |
| Lack of weight over a 2-mo period | 3.7-33.1 | 63.0-93.5 | 0.00-0.01 | 35.5-83.3 | 15.4-48.7 |
| Lack of weight over a 3-mo period | 0.0-3.5 | 93.5-100.0 | 0.00-0.01 | 0.0-100.0 | 15.5-49.2 |
| Loss in LAZ over a 1-mo period | 97.7-100.0 | 0.0-1.5 | 0.00-0.00 | 50.8-84.5 | 0.0-15.0 |
| Loss in LAZ over a 2-mo period | 73.1-98.8 | 0.0-26.3 | 0.00-0.10 | 51.5-85.4 | 0.0-75.0 |
| Loss in LAZ over a 3-mo period | 32.8-73.3 | 29.5-67.8 | 0.00-0.10 | 53.4-85.1 | 16.7-52.3 |
| Lack of length over a 1-mo period | 13.6-94.4 | 2.2-95.7 | 0.00-0.09 | 48.4-94.4 | 11.1-48.1 |
| Lack of length over a 2-mo period | 0.7-18.3 | 73.9-100.0 | 0.00-0.04 | 44.4-100.0 | 15.6-49.1 |
| Lack of length over a 3-mo period | 0.0-1.4 | 97.8-100.0 | 0.00-0.01 | 0.0-100.0 | 15.5-49.2 |
| **Loss in LAZ between 12 and 18 mo** |  |  |  |  |  |
| WAZ < -2 at any point before 12 mo of age | 3.0-30.1 | 55.6-91.7 | 0.00-0.00 | 37.5-74.4 | 17.2-35.9 |
| Loss in WAZ over a 1-mo period | 96.2-100.0 | 0.0-2.2 | 0.00-0.00 | 62.7-79.2 | 0.0-33.3 |
| Loss in WAZ over a 2-mo period | 70.1-96.6 | 4.5-31.5 | 0.00-0.11 | 64.5-79.8 | 26.7-58.3 |
| Loss in WAZ over a 3-mo period | 28.5-68.1 | 28.7-73.5 | 0.00-0.14 | 63.0-81.0 | 23.5-41.2 |
| Lack of weight over a 1-mo period | 64.4-94.6 | 4.2-35.9 | 0.00-0.16 | 61.9-78.4 | 18.4-53.8 |
| Lack of weight over a 2-mo period | 6.9-35.4 | 64.6-98.1 | 0.00-0.08 | 60.6-93.3 | 21.2-37.7 |
| Lack of weight over a 3-mo period | 0.0-5.5 | 94.3-100.0 | 0.00-0.02 | 0.0-100.0 | 20.9-37.3 |
| Loss in LAZ over a 1-mo period | 97.4-100.0 | 0.0-0.8 | 0.00-0.00 | 62.7-79.2 | 0.0-12.2 |
| Loss in LAZ over a 2-mo period | 72.2-96.6 | 0.0-22.6 | 0.00-0.05 | 64.0-78.6 | 0.0-54.5 |
| Loss in LAZ over a 3-mo period | 31.9-66.4 | 17.0-64.5 | 0.00-0.10 | 60.5-80.5 | 15.8-42.6 |
| Lack of length over a 1-mo period | 13.9-94.6 | 2.1-89.5 | 0.00-0.07 | 53.0-80.0 | 11.1-34.4 |
| Lack of length over a 2-mo period | 0.0-17.7 | 68.8-100.0 | 0.00-0.01 | 0.0-100.0 | 20.3-37.3 |
| Lack of length over a 3-mo period | 0.0-0.7 | 95.8-100.0 | 0.00-0.00 | 0.0-33.3 | 20.8-36.9 |
| **Loss in LAZ between 18 and 24 mo** |  |  |  |  |  |
| WAZ < -2 at any point before 12 mo of age | 5.3-40.7 | 69.7-94.5 | 0.00-0.13 | 45.9-65.8 | 28.2-63.6 |
| Loss in WAZ over a 1-mo period | 97.2-100.0 | 0.0-4.4 | 0.00-0.02 | 41.2-71.3 | 0.0-100.0 |
| Loss in WAZ over a 2-mo period | 71.0-93.5 | 2.6-33.5 | 0.00-0.06 | 40.0-71.9 | 30.0-49.8 |
| Loss in WAZ over a 3-mo period | 28.2-65.4 | 35.3-72.7 | 0.00-0.07 | 42.5-68.0 | 25.0-60.8 |
| Lack of weight over a 1-mo period | 61.7-94.4 | 5.3-39.1 | 0.00-0.08 | 37.0-70.4 | 28.3-48.7 |
| Lack of weight over a 2-mo period | 5.3-35.5 | 67.1-98.0 | 0.00-0.08 | 30.8-92.3 | 30.9-57.6 |
| Lack of weight over a 3-mo period | 0.0-4.7 | 97.4-100.0 | 0.00-0.02 | 0.0-100.0 | 29.1-59.2 |
| Loss in LAZ over a 1-mo period | 97.9-100.0 | 0.0-3.1 | 0.00-0.01 | 41.2-70.9 | 0.0-50.0 |
| Loss in LAZ over a 2-mo period | 74.9-98.1 | 1.4-28.7 | 0.00-0.04 | 41.7-70.8 | 20.0-75.0 |
| Loss in LAZ over a 3-mo period | 33.3-72.9 | 29.0-67.7 | 0.00-0.07 | 40.2-72.2 | 30.2-57.1 |
| Lack of length over a 1-mo period | 10.4-95.3 | 5.3-84.3 | 0.00-0.07 | 31.1-73.3 | 29.7-53.5 |
| Lack of length over a 2-mo period | 0.0-23.4 | 80.3-100.0 | 0.00-0.04 | 0.0-100.0 | 29.3-58.9 |
| Lack of length over a 3-mo period | 0.0-1.9 | 97.5-100.0 | 0.00-0.01 | 0.0-100.0 | 29.1-58.8 |
| **Loss in LAZ between 12 and 24 mo** |  |  |  |  |  |
| WAZ < -2 at any point before 12 mo of age | 5.0-28.7 | 60.5-92.0 | 0.00-0.02 | 53.6-75.9 | 13.5-35.4 |
| Loss in WAZ over a 1-mo period | 97.1-100.0 | 0.0-3.6 | 0.00-0.04 | 62.7-84.6 | 0.0-100.0 |
| Loss in WAZ over a 2-mo period | 70.6-96.6 | 4.7-35.3 | 0.00-0.11 | 64.6-85.7 | 23.1-46.2 |
| Loss in WAZ over a 3-mo period | 27.9-66.4 | 38.7-73.8 | 0.00-0.15 | 64.0-84.8 | 16.9-37.9 |
| Lack of weight over a 1-mo period | 65.3-94.5 | 4.8-36.0 | 0.00-0.12 | 63.3-82.9 | 13.6-40.8 |
| Lack of weight over a 2-mo period | 5.0-34.5 | 66.7-96.4 | 0.00-0.07 | 58.5-92.3 | 16.6-36.7 |
| Lack of weight over a 3-mo period | 0.0-5.5 | 98.0-100.0 | 0.00-0.05 | 0.0-100.0 | 15.9-37.3 |
| Loss in LAZ over a 1-mo period | 97.7-100.0 | 0.0-1.4 | 0.00-0.00 | 62.6-84.1 | 0.0-15.4 |
| Loss in LAZ over a 2-mo period | 72.3-98.3 | 0.0-24.4 | 0.00-0.08 | 62.4-85.2 | 0.0-50.0 |
| Loss in LAZ over a 3-mo period | 32.2-69.5 | 19.6-66.2 | 0.00-0.06 | 61.5-83.6 | 15.0-36.7 |
| Lack of length over a 1-mo period | 12.0-93.8 | 0.0-87.1 | 0.00-0.01 | 56.0-83.9 | 0.0-34.5 |
| Lack of length over a 2-mo period | 0.0-17.9 | 71.4-100.0 | 0.00-0.01 | 0.0-100.0 | 16.1-37.2 |
| Lack of length over a 3-mo period | 0.0-0.7 | 95.2-100.0 | 0.00-0.00 | 0.0-33.3 | 15.9-37.4 |
| **Loss in LAD between 12 and 18 mo** |  |  |  |  |  |
| WAZ < -2 at any point before 12 mo of age | 4.1-33.3 | 70.0-93.7 | 0.00-0.06 | 50.0-93.0 | 8.0-38.6 |
| Loss in WAZ over a 1-mo period | 96.4-100.0 | 0.0-2.0 | 0.00-0.00 | 60.9-92.3 | 0.0-16.7 |
| Loss in WAZ over a 2-mo period | 70.8-95.8 | 0.0-35.2 | 0.00-0.11 | 63.0-91.9 | 0.0-52.2 |
| Loss in WAZ over a 3-mo period | 28.7-68.6 | 28.7-75.4 | 0.00-0.20 | 69.7-91.1 | 5.9-47.1 |
| Lack of weight over a 1-mo period | 63.3-95.3 | 4.9-43.3 | 0.00-0.13 | 59.0-90.2 | 2.6-35.9 |
| Lack of weight over a 2-mo period | 7.1-35.5 | 65.4-100.0 | 0.00-0.10 | 70.0-100.0 | 8.5-39.7 |
| Lack of weight over a 3-mo period | 0.0-5.5 | 94.3-100.0 | 0.00-0.01 | 0.0-100.0 | 7.8-39.1 |
| Loss in LAZ over a 1-mo period | 97.7-100.0 | 0.0-0.8 | 0.00-0.00 | 60.9-92.3 | 0.0-7.3 |
| Loss in LAZ over a 2-mo period | 72.6-97.0 | 0.0-21.8 | 0.00-0.05 | 62.0-92.1 | 0.0-54.5 |
| Loss in LAZ over a 3-mo period | 32.6-68.3 | 15.6-64.9 | 0.00-0.11 | 65.6-92.7 | 8.3-45.6 |
| Lack of length over a 1-mo period | 13.5-95.3 | 3.8-90.0 | 0.00-0.09 | 57.6-93.2 | 8.1-37.3 |
| Lack of length over a 2-mo period | 0.0-17.8 | 57.7-100.0 | 0.00-0.02 | 0.0-100.0 | 7.8-39.2 |
| Lack of length over a 3-mo period | 0.0-0.6 | 92.3-100.0 | 0.00-0.00 | 0.0-33.3 | 7.7-38.8 |
| **Loss in LAD between 18 and 24 mo** |  |  |  |  |  |
| WAZ < -2 at any point before 12 mo of age | 7.0-39.8 | 76.5-96.2 | 0.00-0.19 | 62.5-79.6 | 23.7-53.2 |
| Loss in WAZ over a 1-mo period | 96.8-100.0 | 0.0-4.3 | 0.00-0.02 | 47.7-76.4 | 0.0-100.0 |
| Loss in WAZ over a 2-mo period | 70.5-95.2 | 2.0-34.1 | 0.00-0.05 | 49.2-76.2 | 10.0-61.9 |
| Loss in WAZ over a 3-mo period | 27.8-68.3 | 41.2-72.3 | 0.00-0.16 | 51.4-77.7 | 26.2-56.0 |
| Lack of weight over a 1-mo period | 65.4-95.5 | 7.8-40.3 | 0.00-0.11 | 49.5-75.7 | 23.3-55.8 |
| Lack of weight over a 2-mo period | 4.2-37.1 | 72.5-95.2 | 0.00-0.10 | 37.5-84.6 | 24.7-51.8 |
| Lack of weight over a 3-mo period | 0.0-5.3 | 98.3-100.0 | 0.00-0.05 | 0.0-100.0 | 24.0-52.3 |
| Loss in LAZ over a 1-mo period | 97.7-100.0 | 0.0-3.0 | 0.00-0.01 | 47.7-76.0 | 0.0-30.4 |
| Loss in LAZ over a 2-mo period | 74.6-98.1 | 2.0-29.8 | 0.00-0.05 | 47.1-77.0 | 16.7-50.0 |
| Loss in LAZ over a 3-mo period | 33.3-75.0 | 31.5-68.5 | 0.00-0.08 | 51.2-77.4 | 26.0-57.1 |
| Lack of length over a 1-mo period | 12.0-95.5 | 5.9-85.7 | 0.00-0.04 | 35.7-80.0 | 24.8-45.2 |
| Lack of length over a 2-mo period | 0.0-23.5 | 82.4-100.0 | 0.00-0.06 | 0.0-100.0 | 24.1-52.7 |
| Lack of length over a 3-mo period | 0.0-1.5 | 97.3-100.0 | 0.00-0.01 | 0.0-100.0 | 24.0-52.7 |
| **Loss in LAD between 12 and 24 mo** |  |  |  |  |  |
| WAZ < -2 at any point before 12 mo of age | 6.0-34.2 | 75.0-94.1 | 0.00-0.21 | 55.6-97.6 | 7.5-45.1 |
| Loss in WAZ over a 1-mo period | 97.2-100.0 | 0.0-5.5 | 0.00-0.04 | 55.0-93.6 | 0.0-100.0 |
| Loss in WAZ over a 2-mo period | 69.1-96.5 | 6.2-37.9 | 0.01-0.16 | 57.7-93.8 | 9.1-61.9 |
| Loss in WAZ over a 3-mo period | 26.8-64.3 | 33.3-72.4 | 0.00-0.22 | 66.2-93.7 | 4.7-55.8 |
| Lack of weight over a 1-mo period | 66.2-95.4 | 16.7-44.4 | 0.00-0.19 | 56.1-94.4 | 10.3-47.2 |
| Lack of weight over a 2-mo period | 6.0-34.9 | 75.0-100.0 | 0.00-0.11 | 62.5-100.0 | 7.3-45.5 |
| Lack of weight over a 3-mo period | 0.0-4.6 | 97.1-100.0 | 0.00-0.05 | 0.0-100.0 | 6.7-45.0 |
| Loss in LAZ over a 1-mo period | 97.8-100.0 | 0.0-0.7 | 0.00-0.00 | 55.0-93.6 | 0.0-2.5 |
| Loss in LAZ over a 2-mo period | 72.7-97.8 | 0.0-22.8 | 0.00-0.12 | 55.7-93.4 | 0.0-54.5 |
| Loss in LAZ over a 3-mo period | 32.5-71.3 | 22.2-65.5 | 0.00-0.14 | 60.9-92.6 | 5.1-53.1 |
| Lack of length over a 1-mo period | 11.2-94.9 | 0.0-88.9 | 0.00-0.12 | 50.0-93.3 | 0.0-42.1 |
| Lack of length over a 2-mo period | 0.0-19.4 | 66.7-100.0 | 0.00-0.02 | 0.0-100.0 | 5.4-44.6 |
| Lack of length over a 3-mo period | 0.0-1.1 | 91.7-100.0 | 0.00-0.00 | 0.0-66.7 | 6.0-44.7 |

# **Supplementary Table 8**: Predictive accuracy of weight-related GMP, MUAC, and WLZ-based criteria for predicting acute malnutrition in Mali and Burkina Faso among children without acute malnutrition at the time of measurement.

The values in the table show the minimum and maximum values across the 2 countries for ages 7 to 18 mo and for Mali for ages 19 to 24 mo. Youden’s index is defined as sensitivity+specificity-1. MUAC was not evaluated for children < 6 mo of age. Abbreviations: mo = month(s), WAZ = weight-for-age z-score; WLZ = weight-for-length z-score; MUAC = middle upper arm circumference, mm = millimeter

|  | **Sensitivity, %** | **Specificity, %** | **Youden’s index** | **Positive predictive value, %** | **Negative predictive value, %** |
| --- | --- | --- | --- | --- | --- |
| **Outcome: Wasting at 7 months** |  |  |  |  |  |
| WAZ < -2 in the preceding month | 23.3-27.5 | 91.1-96.7 | 0.14-0.24 | 26.9-37.9 | 89.4-94.8 |
| Decrease in WAZ over the preceding month |  |  |  |  |  |
| Decrease in WAZ over the 2 preceding months |  |  |  |  |  |
| Decrease in WAZ over the 3 preceding months |  |  |  |  |  |
| Lack of weight gain over the preceding month |  |  |  |  |  |
| Lack of weight gain over the 2 preceding months |  |  |  |  |  |
| Lack of weight gain over the 3 preceding months |  |  |  |  |  |
| Weight-for-length (WLZ)<-1.0 in the preceding month | 47.5-65.0 | 75.1-86.9 | 0.34-0.40 | 21.1-26.9 | 93.8-95.7 |
| Weight-for-length (WLZ)<-1.5 in the preceding month | 22.5-26.7 | 93.4-96.9 | 0.19-0.20 | 34.6-36.4 | 90.0-94.4 |
| Decrease in WLZ over the preceding month |  |  |  |  |  |
| Decrease in WLZ over the 2 preceding months |  |  |  |  |  |
| Decrease in WLZ over the 3 preceding months |  |  |  |  |  |
| MUAC < 130 mm in the preceding month | 34.5-66.7 | 83.3-88.2 | 0.23-0.50 | 14.9-36.0 | 94.7-95.7 |
| Decrease in MUAC over the preceding month |  |  |  |  |  |
| Decrease in MUAC over the 2 preceding months |  |  |  |  |  |
| Decrease in MUAC over the 3 preceding months |  |  |  |  |  |
| **Outcome: Wasting at 8 months** |  |  |  |  |  |
| WAZ < -2 in the preceding month | 22.2-25.6 | 93.5-95.3 | 0.18-0.19 | 24.2-31.4 | 91.6-94.8 |
| Decrease in WAZ over the preceding month | 66.7-100.0 | 0.2-40.1 | 0.00-0.07 | 6.3-11.0 | 91.6-100.0 |
| Decrease in WAZ over the 2 preceding months |  |  |  |  |  |
| Decrease in WAZ over the 3 preceding months |  |  |  |  |  |
| Lack of weight gain over the preceding month | 30.8-69.0 | 41.3-75.3 | 0.06-0.10 | 7.3-12.1 | 90.8-95.2 |
| Lack of weight gain over the 2 preceding months |  |  |  |  |  |
| Lack of weight gain over the 3 preceding months |  |  |  |  |  |
| Weight-for-length (WLZ)<-1.0 in the preceding month | 47.2-62.8 | 78.7-87.4 | 0.35-0.42 | 20.2-25.5 | 94.8-96.1 |
| Weight-for-length (WLZ)<-1.5 in the preceding month | 13.9-44.2 | 94.1-96.1 | 0.10-0.38 | 19.2-46.3 | 93.6-94.3 |
| Decrease in WLZ over the preceding month | 64.1-100.0 | 0.2-40.1 | 0.00-0.04 | 6.3-10.6 | 91.0-100.0 |
| Decrease in WLZ over the 2 preceding months |  |  |  |  |  |
| Decrease in WLZ over the 3 preceding months |  |  |  |  |  |
| MUAC < 130 mm in the preceding month | 30.6-46.5 | 87.1-92.1 | 0.23-0.34 | 20.8-29.4 | 93.4-95.2 |
| Decrease in MUAC over the preceding month | 37.9-59.0 | 52.3-68.7 | 0.07-0.11 | 7.5-12.0 | 92.0-94.3 |
| Decrease in MUAC over the 2 preceding months |  |  |  |  |  |
| Decrease in MUAC over the 3 preceding months |  |  |  |  |  |
| **Outcome: Wasting at 9 months** |  |  |  |  |  |
| WAZ < -2 in the preceding month | 22.2-36.7 | 92.7-95.0 | 0.17-0.29 | 18.2-29.7 | 94.5-96.1 |
| Decrease in WAZ over the preceding month | 90.5-100.0 | 0.0-17.2 | 0.00-0.08 | 4.5-6.6 | 0.0-96.6 |
| Decrease in WAZ over the 2 preceding months | 40.0-87.5 | 15.1-66.9 | 0.03-0.07 | 4.6-7.2 | 94.5-96.3 |
| Decrease in WAZ over the 3 preceding months |  |  |  |  |  |
| Lack of weight gain over the preceding month | 47.6-85.0 | 27.8-56.4 | 0.04-0.13 | 5.2-6.6 | 94.4-97.5 |
| Lack of weight gain over the 2 preceding months | 5.0-12.5 | 89.0-95.8 | 0.01-0.01 | 5.0-7.1 | 94.0-95.6 |
| Lack of weight gain over the 3 preceding months |  |  |  |  |  |
| Weight-for-length (WLZ)<-1.0 in the preceding month | 59.3-66.7 | 79.7-84.4 | 0.44-0.46 | 16.0-21.7 | 96.6-97.6 |
| Weight-for-length (WLZ)<-1.5 in the preceding month | 29.6-36.7 | 93.9-94.1 | 0.24-0.31 | 19.5-34.4 | 94.6-96.4 |
| Decrease in WLZ over the preceding month | 90.5-100.0 | 0.2-16.9 | 0.00-0.07 | 4.5-6.6 | 96.5-100.0 |
| Decrease in WLZ over the 2 preceding months | 45.0-87.5 | 23.3-70.1 | 0.11-0.15 | 5.0-8.8 | 95.2-97.6 |
| Decrease in WLZ over the 3 preceding months |  |  |  |  |  |
| MUAC < 130 mm in the preceding month | 48.1-56.7 | 91.0-91.5 | 0.40-0.48 | 22.0-34.7 | 96.1-97.2 |
| Decrease in MUAC over the preceding month | 75.0-81.0 | 27.0-40.7 | 0.08-0.16 | 5.6-6.7 | 95.7-97.2 |
| Decrease in MUAC over the 2 preceding months | 18.8-30.0 | 81.7-88.7 | 0.08-0.12 | 7.1-9.5 | 94.8-95.9 |
| Decrease in MUAC over the 3 preceding months |  |  |  |  |  |
| **Outcome: Wasting at 10 months** |  |  |  |  |  |
| WAZ < -2 in the preceding month | 29.2-33.3 | 94.1-95.3 | 0.25-0.27 | 21.9-22.2 | 96.5-96.8 |
| Decrease in WAZ over the preceding month | 93.3-100.0 | 0.0-9.6 | 0.00-0.03 | 4.0-4.7 | 0.0-96.8 |
| Decrease in WAZ over the 2 preceding months | 69.2-100.0 | 11.3-57.8 | 0.11-0.27 | 4.4-6.9 | 97.6-100.0 |
| Decrease in WAZ over the 3 preceding months | 8.3-63.6 | 49.6-84.0 | 0.00-0.13 | 2.2-4.8 | 95.5-97.2 |
| Lack of weight gain over the preceding month | 73.3-94.4 | 19.3-44.1 | 0.14-0.17 | 4.7-5.9 | 97.2-98.8 |
| Lack of weight gain over the 2 preceding months | 15.4-42.9 | 84.9-94.1 | 0.10-0.28 | 10.3-10.5 | 96.1-97.3 |
| Lack of weight gain over the 3 preceding months | 0.0-0.0 | 98.6-100.0 | 0.00-0.00 | 0.0-0.0 | 95.8-96.1 |
| Weight-for-length (WLZ)<-1.0 in the preceding month | 44.4-62.5 | 77.7-81.7 | 0.22-0.44 | 9.2-13.3 | 96.5-98.0 |
| Weight-for-length (WLZ)<-1.5 in the preceding month | 11.1-37.5 | 93.6-95.5 | 0.07-0.31 | 11.1-20.9 | 95.5-97.1 |
| Decrease in WLZ over the preceding month | 86.7-100.0 | 0.0-8.7 | 0.00-0.00 | 4.0-4.4 | 0.0-93.1 |
| Decrease in WLZ over the 2 preceding months | 53.8-92.9 | 17.7-60.6 | 0.11-0.14 | 4.4-5.8 | 96.7-98.4 |
| Decrease in WLZ over the 3 preceding months | 16.7-72.7 | 63.8-90.5 | 0.07-0.37 | 7.1-7.4 | 96.1-98.3 |
| MUAC < 130 mm in the preceding month | 29.2-50.0 | 89.9-91.4 | 0.21-0.40 | 13.2-20.0 | 96.6-97.3 |
| Decrease in MUAC over the preceding month | 83.3-93.3 | 15.1-18.9 | 0.02-0.08 | 4.1-5.0 | 96.4-97.9 |
| Decrease in MUAC over the 2 preceding months | 38.5-42.9 | 71.1-78.0 | 0.10-0.21 | 5.7-7.3 | 96.2-97.1 |
| Decrease in MUAC over the 3 preceding months | 0.0-16.7 | 93.1-97.1 | 0.00-0.10 | 0.0-9.5 | 96.1-96.2 |
| **Outcome: Wasting at 11 months** |  |  |  |  |  |
| WAZ < -2 in the preceding month | 23.3-50.0 | 93.2-94.6 | 0.18-0.43 | 13.8-25.6 | 93.9-98.8 |
| Decrease in WAZ over the preceding month | 100.0-100.0 | 0.0-4.8 | 0.00-0.05 | 1.3-6.5 | 0.0-100.0 |
| Decrease in WAZ over the 2 preceding months | 66.7-100.0 | 8.1-48.5 | 0.08-0.15 | 1.3-7.1 | 99.3-100.0 |
| Decrease in WAZ over the 3 preceding months | 0.0-57.1 | 48.8-80.7 | 0.00-0.06 | 0.0-7.5 | 94.0-99.1 |
| Lack of weight gain over the preceding month | 75.0-96.8 | 11.1-33.0 | 0.08-0.08 | 1.3-7.1 | 98.0-99.1 |
| Lack of weight gain over the 2 preceding months | 0.0-36.0 | 78.7-92.8 | 0.00-0.15 | 0.0-10.6 | 94.6-98.9 |
| Lack of weight gain over the 3 preceding months | 0.0-4.8 | 96.9-99.6 | 0.00-0.02 | 0.0-10.0 | 93.4-99.3 |
| Weight-for-length (WLZ)<-1.0 in the preceding month | 62.8-75.0 | 76.8-79.6 | 0.42-0.52 | 6.6-19.7 | 96.4-99.3 |
| Weight-for-length (WLZ)<-1.5 in the preceding month | 41.9-62.5 | 94.8-95.6 | 0.37-0.58 | 23.8-39.1 | 95.3-99.2 |
| Decrease in WLZ over the preceding month | 100.0-100.0 | 0.0-2.7 | 0.00-0.03 | 1.2-6.5 | 0.0-100.0 |
| Decrease in WLZ over the 2 preceding months | 33.3-96.0 | 13.2-54.3 | 0.00-0.09 | 0.7-7.2 | 97.9-98.8 |
| Decrease in WLZ over the 3 preceding months | 0.0-52.4 | 60.1-88.3 | 0.00-0.13 | 0.0-8.7 | 94.6-99.2 |
| MUAC < 130 mm in the preceding month | 30.2-50.0 | 89.9-94.4 | 0.25-0.40 | 9.8-30.2 | 94.4-98.8 |
| Decrease in MUAC over the preceding month | 90.3-100.0 | 7.0-8.4 | 0.00-0.07 | 1.3-6.5 | 92.5-100.0 |
| Decrease in MUAC over the 2 preceding months | 0.0-40.0 | 65.9-68.1 | 0.00-0.08 | 0.0-8.1 | 94.2-98.5 |
| Decrease in MUAC over the 3 preceding months | 0.0-14.3 | 89.8-93.5 | 0.00-0.08 | 0.0-13.6 | 93.8-99.2 |
| **Outcome: Wasting at 12 months** |  |  |  |  |  |
| WAZ < -2 in the preceding month | 16.7-42.9 | 92.2-94.9 | 0.09-0.38 | 6.2-29.3 | 97.1-97.2 |
| Decrease in WAZ over the preceding month | 88.9-100.0 | 0.0-2.4 | 0.00-0.00 | 2.4-4.1 | 0.0-88.9 |
| Decrease in WAZ over the 2 preceding months | 62.5-100.0 | 4.3-36.9 | 0.00-0.04 | 2.6-3.8 | 97.4-100.0 |
| Decrease in WAZ over the 3 preceding months | 16.7-61.5 | 42.6-76.4 | 0.00-0.04 | 1.6-4.4 | 96.3-97.6 |
| Lack of weight gain over the preceding month | 77.8-100.0 | 6.9-22.9 | 0.01-0.07 | 2.7-4.4 | 97.4-100.0 |
| Lack of weight gain over the 2 preceding months | 12.5-28.6 | 70.9-89.7 | 0.00-0.02 | 3.1-3.5 | 96.4-97.5 |
| Lack of weight gain over the 3 preceding months | 0.0-0.0 | 94.1-99.6 | 0.00-0.00 | 0.0-0.0 | 95.6-97.8 |
| Weight-for-length (WLZ)<-1.0 in the preceding month | 71.4-83.3 | 76.0-79.9 | 0.47-0.63 | 11.5-12.8 | 98.2-99.4 |
| Weight-for-length (WLZ)<-1.5 in the preceding month | 25.0-57.1 | 92.4-95.6 | 0.21-0.50 | 15.0-27.1 | 97.6-97.8 |
| Decrease in WLZ over the preceding month | 100.0-100.0 | 0.0-1.8 | 0.00-0.02 | 2.7-4.1 | 0.0-100.0 |
| Decrease in WLZ over the 2 preceding months | 75.0-92.9 | 9.6-46.5 | 0.02-0.22 | 3.6-3.7 | 97.3-98.6 |
| Decrease in WLZ over the 3 preceding months | 33.3-61.5 | 54.8-86.5 | 0.16-0.20 | 5.3-5.5 | 97.1-98.3 |
| MUAC < 130 mm in the preceding month | 25.0-28.6 | 90.9-93.6 | 0.16-0.22 | 7.9-18.2 | 96.4-97.5 |
| Decrease in MUAC over the preceding month | 100.0-100.0 | 2.1-3.3 | 0.02-0.03 | 2.7-4.2 | 100.0-100.0 |
| Decrease in MUAC over the 2 preceding months | 25.0-50.0 | 58.5-58.9 | 0.00-0.09 | 1.6-4.3 | 96.7-96.9 |
| Decrease in MUAC over the 3 preceding months | 16.7-23.1 | 88.4-91.1 | 0.05-0.14 | 3.1-10.0 | 96.5-97.9 |
| **Outcome: Wasting at 13 months** |  |  |  |  |  |
| WAZ < -2 in the preceding month | 39.4-50.0 | 91.3-92.7 | 0.32-0.41 | 19.0-23.2 | 96.5-97.8 |
| Decrease in WAZ over the preceding month | 100.0-100.0 | 0.0-0.6 | 0.00-0.01 | 3.3-4.0 | 0.0-100.0 |
| Decrease in WAZ over the 2 preceding months | 90.0-100.0 | 4.3-31.6 | 0.04-0.22 | 3.8-4.1 | 99.0-100.0 |
| Decrease in WAZ over the 3 preceding months | 33.3-60.0 | 37.9-71.4 | 0.00-0.05 | 2.8-3.6 | 96.9-97.1 |
| Lack of weight gain over the preceding month | 91.7-95.0 | 3.7-12.6 | 0.00-0.04 | 3.4-3.9 | 94.7-97.8 |
| Lack of weight gain over the 2 preceding months | 10.0-60.0 | 66.2-86.5 | 0.00-0.26 | 2.3-6.3 | 96.8-97.7 |
| Lack of weight gain over the 3 preceding months | 0.0-10.0 | 93.1-99.3 | 0.00-0.03 | 0.0-4.2 | 96.9-97.2 |
| Weight-for-length (WLZ)<-1.0 in the preceding month | 75.8-87.5 | 73.1-79.6 | 0.49-0.67 | 13.6-14.9 | 98.2-99.4 |
| Weight-for-length (WLZ)<-1.5 in the preceding month | 43.8-51.5 | 89.7-94.6 | 0.38-0.41 | 21.8-25.0 | 97.1-97.6 |
| Decrease in WLZ over the preceding month | 91.7-100.0 | 0.0-0.6 | 0.00-0.00 | 3.0-4.0 | 0.0-66.7 |
| Decrease in WLZ over the 2 preceding months | 90.0-93.3 | 8.7-37.7 | 0.02-0.28 | 3.8-4.5 | 97.1-99.2 |
| Decrease in WLZ over the 3 preceding months | 33.3-40.0 | 49.9-83.9 | 0.00-0.17 | 2.3-6.2 | 96.5-97.5 |
| MUAC < 130 mm in the preceding month | 30.3-50.0 | 92.1-93.9 | 0.24-0.42 | 20.5-21.7 | 96.0-97.8 |
| Decrease in MUAC over the preceding month | 100.0-100.0 | 0.8-0.8 | 0.01-0.01 | 3.3-4.0 | 100.0-100.0 |
| Decrease in MUAC over the 2 preceding months | 40.0-80.0 | 48.9-53.9 | 0.00-0.34 | 2.9-5.3 | 95.5-98.8 |
| Decrease in MUAC over the 3 preceding months | 0.0-22.2 | 86.8-89.3 | 0.00-0.09 | 0.0-5.1 | 96.8-97.2 |
| **Outcome: Wasting at 14 months** |  |  |  |  |  |
| WAZ < -2 in the preceding month | 25.5-66.7 | 91.6-92.2 | 0.17-0.59 | 19.7-24.4 | 93.8-98.7 |
| Decrease in WAZ over the preceding month | 100.0-100.0 | 0.0-0.3 | 0.00-0.00 | 2.7-6.2 | 0.0-100.0 |
| Decrease in WAZ over the 2 preceding months | 37.5-100.0 | 4.4-19.6 | 0.00-0.04 | 1.1-5.8 | 92.8-100.0 |
| Decrease in WAZ over the 3 preceding months | 40.0-76.5 | 35.0-63.9 | 0.04-0.12 | 1.9-5.6 | 96.7-98.4 |
| Lack of weight gain over the preceding month | 97.0-100.0 | 1.6-7.9 | 0.00-0.08 | 3.0-6.1 | 88.9-100.0 |
| Lack of weight gain over the 2 preceding months | 12.5-45.8 | 62.7-78.2 | 0.00-0.09 | 1.4-6.7 | 95.2-97.3 |
| Lack of weight gain over the 3 preceding months | 0.0-5.9 | 92.6-99.0 | 0.00-0.00 | 0.0-3.8 | 95.2-98.3 |
| Weight-for-length (WLZ)<-1.0 in the preceding month | 63.8-93.3 | 70.7-74.8 | 0.35-0.68 | 12.3-14.9 | 96.0-99.7 |
| Weight-for-length (WLZ)<-1.5 in the preceding month | 46.8-66.7 | 89.7-92.7 | 0.37-0.59 | 25.6-26.8 | 95.4-98.7 |
| Decrease in WLZ over the preceding month | 100.0-100.0 | 0.0-0.3 | 0.00-0.00 | 2.7-6.2 | 0.0-100.0 |
| Decrease in WLZ over the 2 preceding months | 62.5-100.0 | 6.4-24.5 | 0.00-0.06 | 2.0-5.9 | 96.4-100.0 |
| Decrease in WLZ over the 3 preceding months | 20.0-64.7 | 46.5-76.7 | 0.00-0.11 | 1.5-5.7 | 96.3-98.2 |
| MUAC < 130 mm in the preceding month | 21.3-26.7 | 92.9-93.7 | 0.15-0.20 | 12.5-21.3 | 93.7-97.1 |
| Decrease in MUAC over the preceding month | 97.0-100.0 | 0.6-0.6 | 0.00-0.01 | 2.7-6.1 | 75.0-100.0 |
| Decrease in MUAC over the 2 preceding months | 50.0-75.0 | 42.2-47.9 | 0.00-0.17 | 2.3-7.1 | 96.6-97.5 |
| Decrease in MUAC over the 3 preceding months | 23.5-40.0 | 83.7-85.9 | 0.09-0.24 | 4.1-7.7 | 95.7-98.8 |
| **Outcome: Wasting at 15 months** |  |  |  |  |  |
| WAZ < -2 in the preceding month | 40.0-40.6 | 88.0-88.5 | 0.28-0.29 | 7.8-15.3 | 96.7-98.3 |
| Decrease in WAZ over the preceding month | 100.0-100.0 | 0.0-0.0 | 0.00-0.00 | 2.2-3.8 | 0.0-0.0 |
| Decrease in WAZ over the 2 preceding months | 80.0-92.9 | 3.1-13.7 | 0.00-0.00 | 1.4-2.8 | 93.3-97.8 |
| Decrease in WAZ over the 3 preceding months | 40.0-50.0 | 30.7-53.1 | 0.00-0.00 | 1.4-1.9 | 95.9-98.2 |
| Lack of weight gain over the preceding month | 95.2-100.0 | 0.4-5.0 | 0.00-0.05 | 2.3-3.6 | 66.7-100.0 |
| Lack of weight gain over the 2 preceding months | 35.7-40.0 | 60.0-72.0 | 0.00-0.12 | 2.1-2.7 | 96.8-98.7 |
| Lack of weight gain over the 3 preceding months | 0.0-0.0 | 92.1-98.3 | 0.00-0.00 | 0.0-0.0 | 97.2-98.3 |
| Weight-for-length (WLZ)<-1.0 in the preceding month | 65.6-90.0 | 71.0-74.9 | 0.37-0.65 | 8.4-10.4 | 97.6-99.7 |
| Weight-for-length (WLZ)<-1.5 in the preceding month | 40.6-60.0 | 90.2-90.5 | 0.31-0.51 | 14.0-17.6 | 96.7-98.9 |
| Decrease in WLZ over the preceding month | 100.0-100.0 | 0.0-0.0 | 0.00-0.00 | 2.2-3.8 | 0.0-0.0 |
| Decrease in WLZ over the 2 preceding months | 60.0-78.6 | 5.0-19.5 | 0.00-0.00 | 1.1-2.5 | 88.5-97.0 |
| Decrease in WLZ over the 3 preceding months | 40.0-40.0 | 42.3-65.3 | 0.00-0.05 | 1.8-1.9 | 96.4-98.5 |
| MUAC < 130 mm in the preceding month | 25.0-40.0 | 92.6-94.9 | 0.18-0.35 | 14.8-16.7 | 96.0-98.4 |
| Decrease in MUAC over the preceding month | 100.0-100.0 | 0.0-0.2 | 0.00-0.00 | 2.2-3.8 | 0.0-100.0 |
| Decrease in MUAC over the 2 preceding months | 78.6-80.0 | 37.6-40.5 | 0.16-0.21 | 2.0-3.7 | 98.3-99.3 |
| Decrease in MUAC over the 3 preceding months | 20.0-40.0 | 78.2-82.9 | 0.00-0.23 | 1.5-5.8 | 98.1-98.3 |
| **Outcome: Wasting at 16 months** |  |  |  |  |  |
| WAZ < -2 in the preceding month | 42.2-44.4 | 88.1-90.0 | 0.32-0.33 | 7.8-22.6 | 95.7-98.6 |
| Decrease in WAZ over the preceding month | 100.0-100.0 | 0.0-0.0 | 0.00-0.00 | 1.9-5.3 | 0.0-0.0 |
| Decrease in WAZ over the 2 preceding months | 100.0-100.0 | 2.3-10.4 | 0.02-0.10 | 1.3-3.9 | 100.0-100.0 |
| Decrease in WAZ over the 3 preceding months | 50.0-78.6 | 26.7-50.5 | 0.00-0.05 | 1.3-3.4 | 97.4-98.7 |
| Lack of weight gain over the preceding month | 100.0-100.0 | 0.0-3.9 | 0.00-0.04 | 2.0-5.3 | 0.0-100.0 |
| Lack of weight gain over the 2 preceding months | 36.8-50.0 | 55.6-68.7 | 0.00-0.19 | 1.9-3.2 | 95.7-99.1 |
| Lack of weight gain over the 3 preceding months | 0.0-0.0 | 91.2-97.0 | 0.00-0.00 | 0.0-0.0 | 96.5-98.7 |
| Weight-for-length (WLZ)<-1.0 in the preceding month | 68.9-88.9 | 70.5-73.9 | 0.39-0.63 | 7.2-14.0 | 97.0-99.7 |
| Weight-for-length (WLZ)<-1.5 in the preceding month | 48.9-66.7 | 91.0-91.9 | 0.40-0.59 | 15.8-27.5 | 96.2-99.2 |
| Decrease in WLZ over the preceding month | 100.0-100.0 | 0.0-0.0 | 0.00-0.00 | 1.9-5.3 | 0.0-0.0 |
| Decrease in WLZ over the 2 preceding months | 75.0-100.0 | 4.6-14.0 | 0.00-0.05 | 1.0-4.0 | 97.9-100.0 |
| Decrease in WLZ over the 3 preceding months | 25.0-85.7 | 42.1-60.7 | 0.00-0.28 | 0.8-4.7 | 98.4-98.9 |
| MUAC < 130 mm in the preceding month | 44.4-44.4 | 92.1-93.9 | 0.37-0.38 | 14.3-28.2 | 96.0-98.7 |
| Decrease in MUAC over the preceding month | 100.0-100.0 | 0.0-0.2 | 0.00-0.00 | 1.9-5.3 | 0.0-100.0 |
| Decrease in MUAC over the 2 preceding months | 78.9-100.0 | 29.3-32.8 | 0.08-0.33 | 1.7-4.2 | 97.2-100.0 |
| Decrease in MUAC over the 3 preceding months | 14.3-50.0 | 72.9-79.3 | 0.00-0.23 | 2.2-2.4 | 96.5-99.1 |
| **Outcome: Wasting at 17 months** |  |  |  |  |  |
| WAZ < -2 in the preceding month | 29.4-33.3 | 88.0-91.1 | 0.21-0.21 | 9.8-14.7 | 96.1-97.1 |
| Decrease in WAZ over the preceding month | 100.0-100.0 | 0.0-0.0 | 0.00-0.00 | 3.0-3.3 | 0.0-0.0 |
| Decrease in WAZ over the 2 preceding months | 100.0-100.0 | 1.6-7.9 | 0.02-0.08 | 2.7-3.3 | 100.0-100.0 |
| Decrease in WAZ over the 3 preceding months | 62.5-62.5 | 25.8-47.7 | 0.00-0.10 | 1.5-3.1 | 97.4-97.9 |
| Lack of weight gain over the preceding month | 100.0-100.0 | 0.0-2.9 | 0.00-0.03 | 3.0-3.4 | 0.0-100.0 |
| Lack of weight gain over the 2 preceding months | 35.7-50.0 | 52.7-65.6 | 0.00-0.16 | 2.0-4.4 | 96.7-97.7 |
| Lack of weight gain over the 3 preceding months | 0.0-12.5 | 90.5-96.7 | 0.00-0.03 | 0.0-2.3 | 97.3-98.3 |
| Weight-for-length (WLZ)<-1.0 in the preceding month | 76.5-80.0 | 69.3-74.7 | 0.49-0.51 | 9.2-13.6 | 98.4-98.9 |
| Weight-for-length (WLZ)<-1.5 in the preceding month | 33.3-47.1 | 91.4-92.0 | 0.25-0.39 | 13.2-23.5 | 97.1-97.2 |
| Decrease in WLZ over the preceding month | 100.0-100.0 | 0.0-0.0 | 0.00-0.00 | 3.0-3.3 | 0.0-0.0 |
| Decrease in WLZ over the 2 preceding months | 90.0-100.0 | 3.4-10.7 | 0.01-0.03 | 2.8-3.1 | 97.1-100.0 |
| Decrease in WLZ over the 3 preceding months | 50.0-75.0 | 40.3-59.0 | 0.09-0.15 | 2.2-3.1 | 97.8-98.9 |
| MUAC < 130 mm in the preceding month | 40.0-41.2 | 93.4-94.8 | 0.35-0.35 | 23.1-24.6 | 96.8-97.6 |
| Decrease in MUAC over the preceding month | 100.0-100.0 | 0.0-0.0 | 0.00-0.00 | 3.0-3.3 | 0.0-0.0 |
| Decrease in MUAC over the 2 preceding months | 70.0-71.4 | 26.1-28.1 | 0.00-0.00 | 2.6-3.0 | 96.7-97.1 |
| Decrease in MUAC over the 3 preceding months | 12.5-25.0 | 70.0-73.3 | 0.00-0.00 | 1.1-1.7 | 96.8-98.2 |
| **Outcome: Wasting at 18 months** |  |  |  |  |  |
| WAZ < -2 in the preceding month | 43.8-50.0 | 87.0-92.2 | 0.36-0.37 | 9.1-21.5 | 97.1-98.5 |
| Decrease in WAZ over the preceding month | 100.0-100.0 | 0.0-0.0 | 0.00-0.00 | 2.6-3.7 | 0.0-0.0 |
| Decrease in WAZ over the 2 preceding months | 85.7-94.1 | 1.5-7.6 | 0.00-0.00 | 2.0-3.0 | 88.9-96.0 |
| Decrease in WAZ over the 3 preceding months | 50.0-60.0 | 24.1-42.2 | 0.00-0.00 | 1.2-2.5 | 94.8-98.4 |
| Lack of weight gain over the preceding month | 100.0-100.0 | 0.2-2.3 | 0.00-0.02 | 2.6-3.7 | 100.0-100.0 |
| Lack of weight gain over the 2 preceding months | 57.1-64.7 | 49.0-64.6 | 0.14-0.22 | 3.5-3.9 | 97.7-98.5 |
| Lack of weight gain over the 3 preceding months | 0.0-33.3 | 90.4-96.2 | 0.00-0.24 | 0.0-10.2 | 97.6-98.6 |
| Weight-for-length (WLZ)<-1.0 in the preceding month | 71.9-80.0 | 69.4-75.5 | 0.47-0.49 | 6.3-12.6 | 98.2-99.3 |
| Weight-for-length (WLZ)<-1.5 in the preceding month | 40.0-53.1 | 92.5-92.7 | 0.33-0.46 | 12.1-26.2 | 97.6-98.3 |
| Decrease in WLZ over the preceding month | 100.0-100.0 | 0.0-0.0 | 0.00-0.00 | 2.6-3.7 | 0.0-0.0 |
| Decrease in WLZ over the 2 preceding months | 85.7-100.0 | 3.4-10.5 | 0.00-0.03 | 2.1-3.2 | 97.1-100.0 |
| Decrease in WLZ over the 3 preceding months | 25.0-60.0 | 38.7-54.7 | 0.00-0.00 | 0.8-3.1 | 96.7-98.1 |
| MUAC < 130 mm in the preceding month | 25.0-40.0 | 91.4-93.3 | 0.18-0.31 | 10.8-15.4 | 96.2-98.3 |
| Decrease in MUAC over the preceding month | 100.0-100.0 | 0.0-0.0 | 0.00-0.00 | 2.6-3.7 | 0.0-0.0 |
| Decrease in MUAC over the 2 preceding months | 70.6-85.7 | 20.7-24.5 | 0.00-0.10 | 2.5-2.8 | 95.6-98.7 |
| Decrease in MUAC over the 3 preceding months | 0.0-26.7 | 67.1-71.3 | 0.00-0.00 | 0.0-3.0 | 96.7-98.0 |
| **Outcome: Wasting at 19 months** |  |  |  |  |  |
| WAZ < -2 in the preceding month | 41.2 | 86.9 | 0.28 | 12.1 | 97.1 |
| Decrease in WAZ over the preceding month | 100.0 | 0.0 | 0.00 | 3.1 | 0.0 |
| Decrease in WAZ over the 2 preceding months | 87.5 | 5.5 | 0.00 | 2.3 | 94.4 |
| Decrease in WAZ over the 3 preceding months | 57.1 | 43.2 | 0.00 | 2.4 | 97.6 |
| Lack of weight gain over the preceding month | 100.0 | 1.7 | 0.02 | 3.1 | 100.0 |
| Lack of weight gain over the 2 preceding months | 37.5 | 62.1 | 0.00 | 2.5 | 97.5 |
| Lack of weight gain over the 3 preceding months | 14.3 | 96.5 | 0.11 | 9.1 | 97.9 |
| Weight-for-length (WLZ)<-1.0 in the preceding month | 70.6 | 74.1 | 0.45 | 10.6 | 98.3 |
| Weight-for-length (WLZ)<-1.5 in the preceding month | 29.4 | 92.8 | 0.22 | 15.2 | 96.8 |
| Decrease in WLZ over the preceding month | 100.0 | 0.0 | 0.00 | 3.1 | 0.0 |
| Decrease in WLZ over the 2 preceding months | 100.0 | 10.0 | 0.10 | 2.8 | 100.0 |
| Decrease in WLZ over the 3 preceding months | 57.1 | 53.3 | 0.10 | 2.9 | 98.1 |
| MUAC < 130 mm in the preceding month | 58.8 | 94.1 | 0.53 | 30.3 | 98.1 |
| Decrease in MUAC over the preceding month | 100.0 | 0.0 | 0.00 | 3.1 | 0.0 |
| Decrease in MUAC over the 2 preceding months | 75.0 | 20.3 | 0.00 | 2.4 | 96.9 |
| Decrease in MUAC over the 3 preceding months | 57.1 | 66.2 | 0.23 | 4.0 | 98.4 |
| **Outcome: Wasting at 20 months** |  |  |  |  |  |
| WAZ < -2 in the preceding month | 50.0 | 86.1 | 0.36 | 14.9 | 97.2 |
| Decrease in WAZ over the preceding month | 100.0 | 0.0 | 0.00 | 4.2 | 0.0 |
| Decrease in WAZ over the 2 preceding months | 100.0 | 6.2 | 0.06 | 4.4 | 100.0 |
| Decrease in WAZ over the 3 preceding months | 50.0 | 40.8 | 0.00 | 3.4 | 95.2 |
| Lack of weight gain over the preceding month | 100.0 | 1.4 | 0.01 | 4.3 | 100.0 |
| Lack of weight gain over the 2 preceding months | 35.7 | 58.1 | 0.00 | 3.6 | 95.4 |
| Lack of weight gain over the 3 preceding months | 8.3 | 95.8 | 0.04 | 7.7 | 96.2 |
| Weight-for-length (WLZ)<-1.0 in the preceding month | 60.0 | 76.1 | 0.36 | 10.9 | 97.5 |
| Weight-for-length (WLZ)<-1.5 in the preceding month | 35.0 | 93.9 | 0.29 | 21.9 | 96.7 |
| Decrease in WLZ over the preceding month | 100.0 | 0.0 | 0.00 | 4.2 | 0.0 |
| Decrease in WLZ over the 2 preceding months | 85.7 | 6.8 | 0.00 | 3.8 | 91.7 |
| Decrease in WLZ over the 3 preceding months | 25.0 | 51.6 | 0.00 | 2.1 | 94.3 |
| MUAC < 130 mm in the preceding month | 30.0 | 93.2 | 0.23 | 17.6 | 96.5 |
| Decrease in MUAC over the preceding month | 100.0 | 0.0 | 0.00 | 4.2 | 0.0 |
| Decrease in MUAC over the 2 preceding months | 85.7 | 17.7 | 0.03 | 4.3 | 96.6 |
| Decrease in MUAC over the 3 preceding months | 50.0 | 66.1 | 0.16 | 5.8 | 97.0 |
|  |  |  |  |  |  |
| **Outcome: Wasting at 21 months** |  |  |  |  |  |
| WAZ < -2 in the preceding month | 37.5 | 87.7 | 0.25 | 5.3 | 98.7 |
| Decrease in WAZ over the preceding month | 100.0 | 0.0 | 0.00 | 1.5 | 0.0 |
| Decrease in WAZ over the 2 preceding months | 100.0 | 5.8 | 0.06 | 1.8 | 100.0 |
| Decrease in WAZ over the 3 preceding months | 83.3 | 37.3 | 0.21 | 2.5 | 99.1 |
| Lack of weight gain over the preceding month | 100.0 | 1.3 | 0.01 | 1.5 | 100.0 |
| Lack of weight gain over the 2 preceding months | 33.3 | 55.1 | 0.00 | 1.3 | 97.9 |
| Lack of weight gain over the 3 preceding months | 0.0 | 94.8 | 0.00 | 0.0 | 98.0 |
| Weight-for-length (WLZ)<-1.0 in the preceding month | 50.0 | 79.5 | 0.30 | 4.3 | 98.9 |
| Weight-for-length (WLZ)<-1.5 in the preceding month | 12.5 | 95.5 | 0.08 | 4.8 | 98.4 |
| Decrease in WLZ over the preceding month | 100.0 | 0.0 | 0.00 | 1.5 | 0.0 |
| Decrease in WLZ over the 2 preceding months | 83.3 | 5.5 | 0.00 | 1.5 | 95.0 |
| Decrease in WLZ over the 3 preceding months | 50.0 | 48.4 | 0.00 | 1.9 | 98.0 |
| MUAC < 130 mm in the preceding month | 37.5 | 93.2 | 0.31 | 9.1 | 98.8 |
| Decrease in MUAC over the preceding month | 100.0 | 0.0 | 0.00 | 1.5 | 0.0 |
| Decrease in MUAC over the 2 preceding months | 100.0 | 14.8 | 0.15 | 2.0 | 100.0 |
| Decrease in MUAC over the 3 preceding months | 33.3 | 64.4 | 0.00 | 1.8 | 98.0 |
| **Outcome: Wasting at 22 months** |  |  |  |  |  |
| WAZ < -2 in the preceding month | 50.0 | 90.1 | 0.40 | 17.0 | 97.8 |
| Decrease in WAZ over the preceding month | 100.0 | 0.0 | 0.00 | 2.8 | 0.0 |
| Decrease in WAZ over the 2 preceding months | 88.9 | 5.7 | 0.00 | 2.2 | 95.5 |
| Decrease in WAZ over the 3 preceding months | 57.1 | 37.8 | 0.00 | 1.9 | 97.7 |
| Lack of weight gain over the preceding month | 100.0 | 1.2 | 0.01 | 2.9 | 100.0 |
| Lack of weight gain over the 2 preceding months | 44.4 | 53.9 | 0.00 | 2.3 | 97.6 |
| Lack of weight gain over the 3 preceding months | 0.0 | 93.4 | 0.00 | 0.0 | 97.8 |
| Weight-for-length (WLZ)<-1.0 in the preceding month | 61.1 | 83.0 | 0.44 | 12.6 | 98.1 |
| Weight-for-length (WLZ)<-1.5 in the preceding month | 33.3 | 96.0 | 0.29 | 25.0 | 97.3 |
| Decrease in WLZ over the preceding month | 100.0 | 0.0 | 0.00 | 2.8 | 0.0 |
| Decrease in WLZ over the 2 preceding months | 77.8 | 4.9 | 0.00 | 1.9 | 90.0 |
| Decrease in WLZ over the 3 preceding months | 28.6 | 47.4 | 0.00 | 1.1 | 96.9 |
| MUAC < 130 mm in the preceding month | 16.7 | 94.6 | 0.11 | 11.1 | 96.6 |
| Decrease in MUAC over the preceding month | 100.0 | 0.0 | 0.00 | 2.8 | 0.0 |
| Decrease in MUAC over the 2 preceding months | 100.0 | 12.1 | 0.12 | 2.7 | 100.0 |
| Decrease in MUAC over the 3 preceding months | 57.1 | 61.0 | 0.18 | 3.0 | 98.5 |
|  |  |  |  |  |  |
| **Outcome: Wasting at 23 months** |  |  |  |  |  |
| WAZ < -2 in the preceding month | 40.0 | 89.7 | 0.30 | 4.1 | 99.3 |
| Decrease in WAZ over the preceding month | 100.0 | 0.0 | 0.00 | 1.2 | 0.0 |
| Decrease in WAZ over the 2 preceding months | 100.0 | 5.1 | 0.05 | 1.1 | 100.0 |
| Decrease in WAZ over the 3 preceding months | 100.0 | 37.9 | 0.38 | 1.3 | 100.0 |
| Lack of weight gain over the preceding month | 100.0 | 0.9 | 0.01 | 1.2 | 100.0 |
| Lack of weight gain over the 2 preceding months | 75.0 | 52.4 | 0.27 | 1.6 | 99.5 |
| Lack of weight gain over the 3 preceding months | 33.3 | 91.9 | 0.25 | 3.3 | 99.4 |
| Weight-for-length (WLZ)<-1.0 in the preceding month | 60.0 | 84.9 | 0.45 | 4.2 | 99.5 |
| Weight-for-length (WLZ)<-1.5 in the preceding month | 40.0 | 94.7 | 0.35 | 7.7 | 99.3 |
| Decrease in WLZ over the preceding month | 100.0 | 0.0 | 0.00 | 1.2 | 0.0 |
| Decrease in WLZ over the 2 preceding months | 100.0 | 4.6 | 0.05 | 1.1 | 100.0 |
| Decrease in WLZ over the 3 preceding months | 33.3 | 46.6 | 0.00 | 0.5 | 98.8 |
| MUAC < 130 mm in the preceding month | 40.0 | 93.9 | 0.34 | 6.7 | 99.3 |
| Decrease in MUAC over the preceding month | 100.0 | 0.0 | 0.00 | 1.2 | 0.0 |
| Decrease in MUAC over the 2 preceding months | 100.0 | 12.2 | 0.12 | 1.1 | 100.0 |
| Decrease in MUAC over the 3 preceding months | 66.7 | 59.3 | 0.26 | 1.4 | 99.5 |
| **Outcome: Wasting at 24 months** |  |  |  |  |  |
| WAZ < -2 in the preceding month | 71.4 | 90.8 | 0.62 | 10.4 | 99.5 |
| Decrease in WAZ over the preceding month | 100.0 | 0.0 | 0.00 | 1.1 | 0.0 |
| Decrease in WAZ over the 2 preceding months | 100.0 | 4.8 | 0.05 | 0.5 | 100.0 |
| Decrease in WAZ over the 3 preceding months | 50.0 | 38.4 | 0.00 | 0.4 | 99.3 |
| Lack of weight gain over the preceding month | 100.0 | 0.2 | 0.00 | 1.1 | 100.0 |
| Lack of weight gain over the 2 preceding months | 50.0 | 50.4 | 0.00 | 0.5 | 99.5 |
| Lack of weight gain over the 3 preceding months | 0.0 | 92.2 | 0.00 | 0.0 | 99.4 |
| Weight-for-length (WLZ)<-1.0 in the preceding month | 71.4 | 86.1 | 0.58 | 7.1 | 99.5 |
| Weight-for-length (WLZ)<-1.5 in the preceding month | 57.1 | 96.6 | 0.54 | 20.0 | 99.3 |
| Decrease in WLZ over the preceding month | 100.0 | 0.0 | 0.00 | 1.1 | 0.0 |
| Decrease in WLZ over the 2 preceding months | 100.0 | 4.6 | 0.05 | 0.5 | 100.0 |
| Decrease in WLZ over the 3 preceding months | 50.0 | 46.7 | 0.00 | 0.5 | 99.4 |
| MUAC < 130 mm in the preceding month | 28.6 | 94.9 | 0.24 | 7.7 | 98.9 |
| Decrease in MUAC over the preceding month | 100.0 | 0.0 | 0.00 | 1.1 | 0.0 |
| Decrease in MUAC over the 2 preceding months | 100.0 | 12.0 | 0.12 | 0.5 | 100.0 |
| Decrease in MUAC over the 3 preceding months | 50.0 | 58.2 | 0.08 | 0.6 | 99.6 |

# **Supplementary Figure 1**: Distribution of one-month weight gain in boys (panels A to D) and girls (E to H) relative to the WHO growth standard between 2 and 3 months (A and E), 5 to 6 months (B and F), 8 to 9 months (C to G), and 11 to 12 months (D to H).

Figures exclude outliers, defined as values larger than 1.5 times the interquartile range (IQR) above or below the median. Countries are sorted in ascending order of prevalence of WAZ < -2 at 24 months of age (18 months in Burkina Faso). Mali is excluded from figures below 6 months of age because study enrollment began at 6 months of age. The thick green horizontal line represents the median of WHO growth standard, the thin blue horizontal lines the 25th and 75th percentiles of the WHO standard, and the dashed red horizontal line 1.5 times the IQR.

| A   | E   |
| --- | --- |
| B   | F   |
| C   | G   |
| D   | H   |

# **Supplementary Figure 2**: Overlap between child stunting, wasting, and underweight in children 12 months of age in Belgium (A), Brazil (B), Peru (C), South Africa (D), Mali (E), Nepal (F), Tanzania (G), Bangladesh - Mirpur (H), India (I), Bangladesh - Matlab (J), and Burkina Faso (K).

Stunting was defined as height-for-age z-score < -2, wasting as weight-for-height z-score < -2, and underweight as weight-for-age z-score < -2. “All other” refers to children who are not stunted, wasted, or underweight.

| A. Belgium  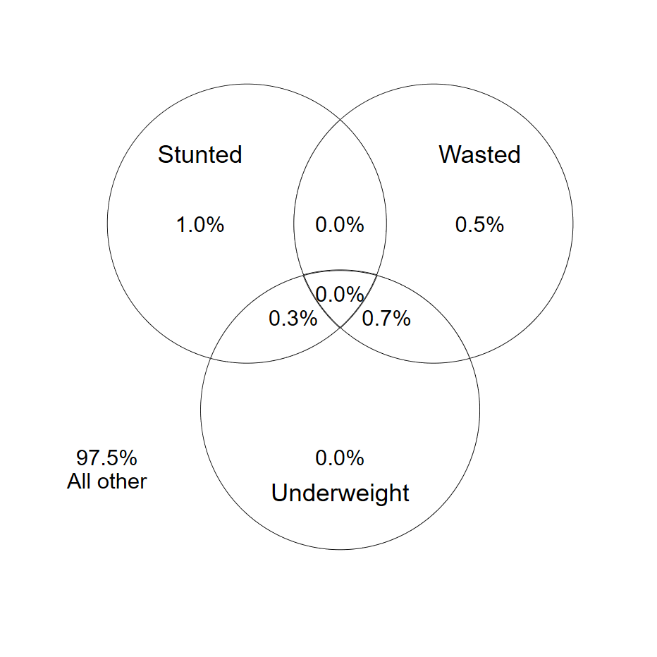 | B. Brazil  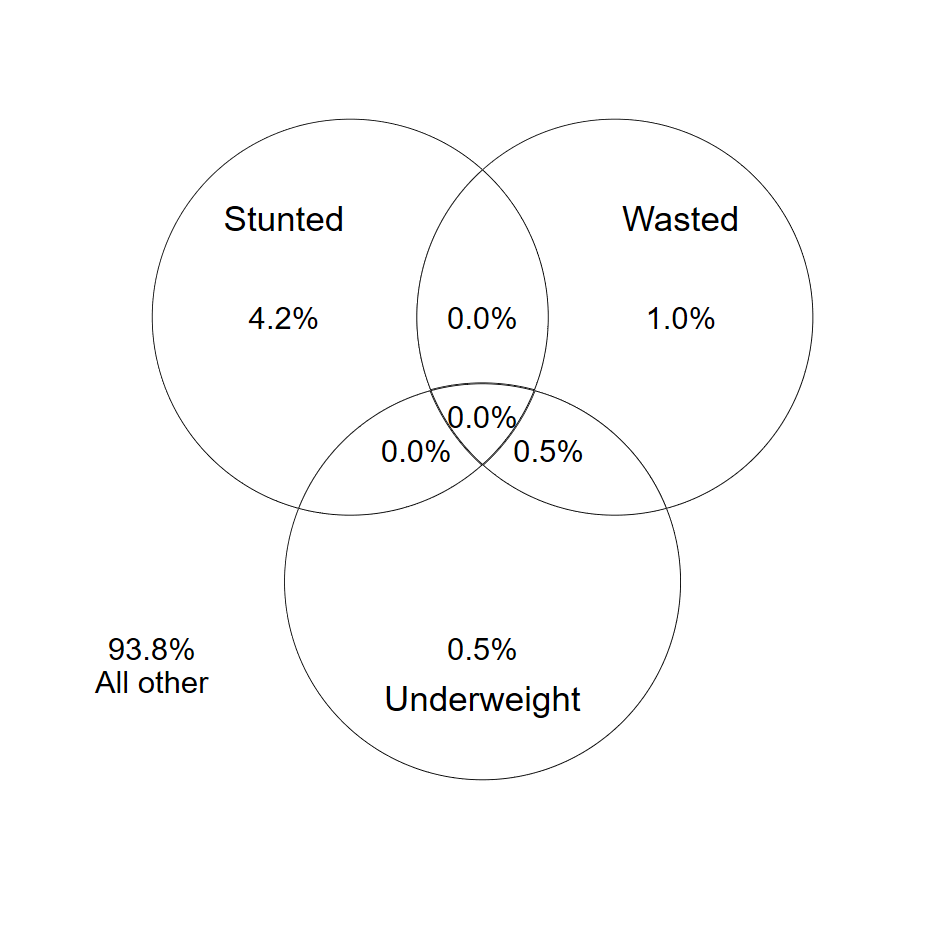 | C. Peru  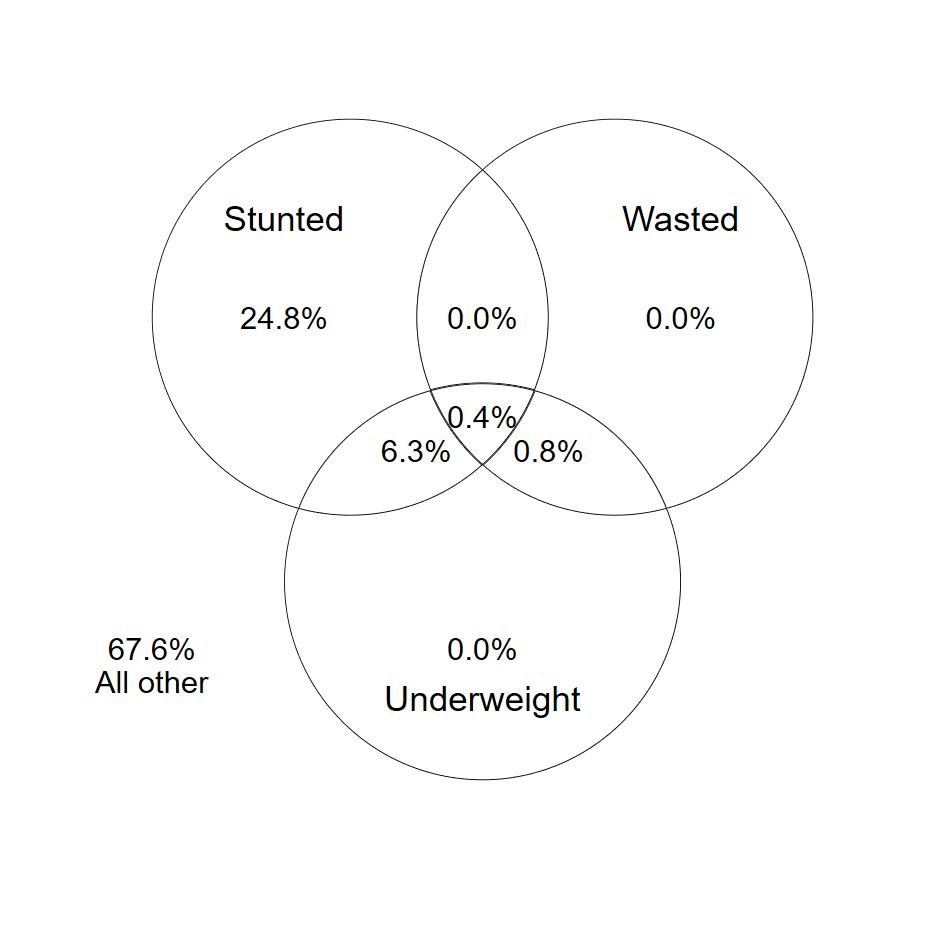 |
| --- | --- | --- |
| D. South Africa  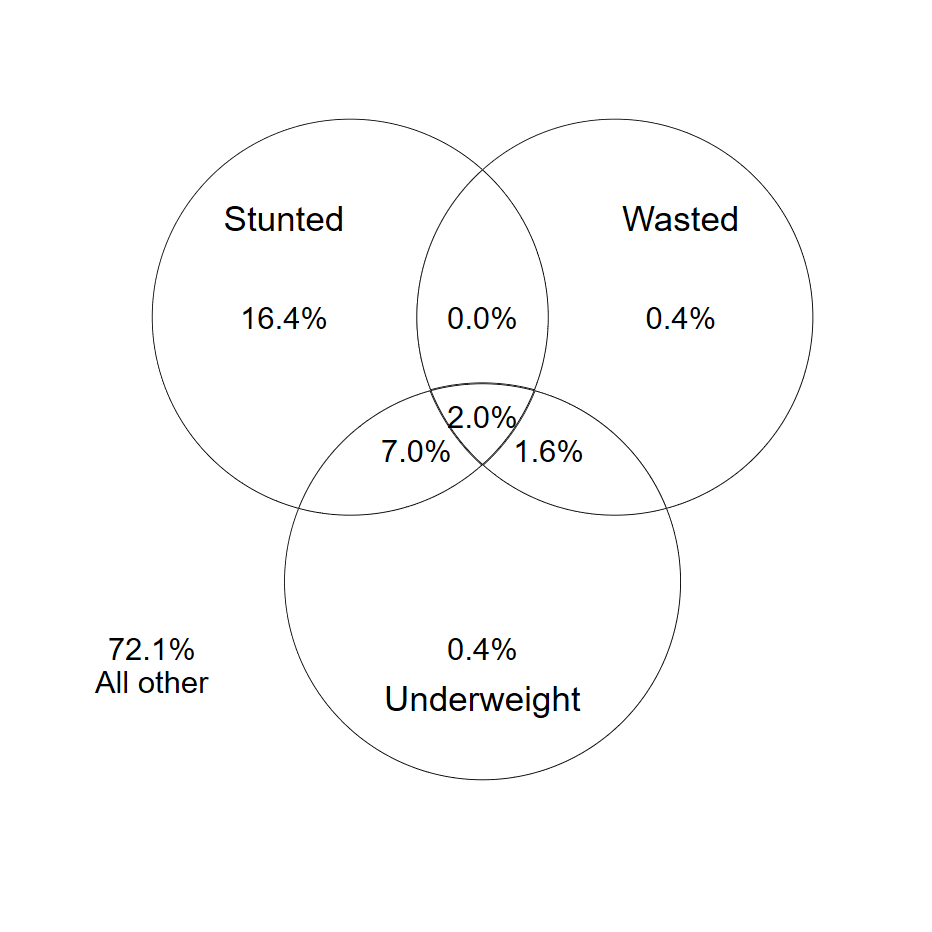 | E. Mali  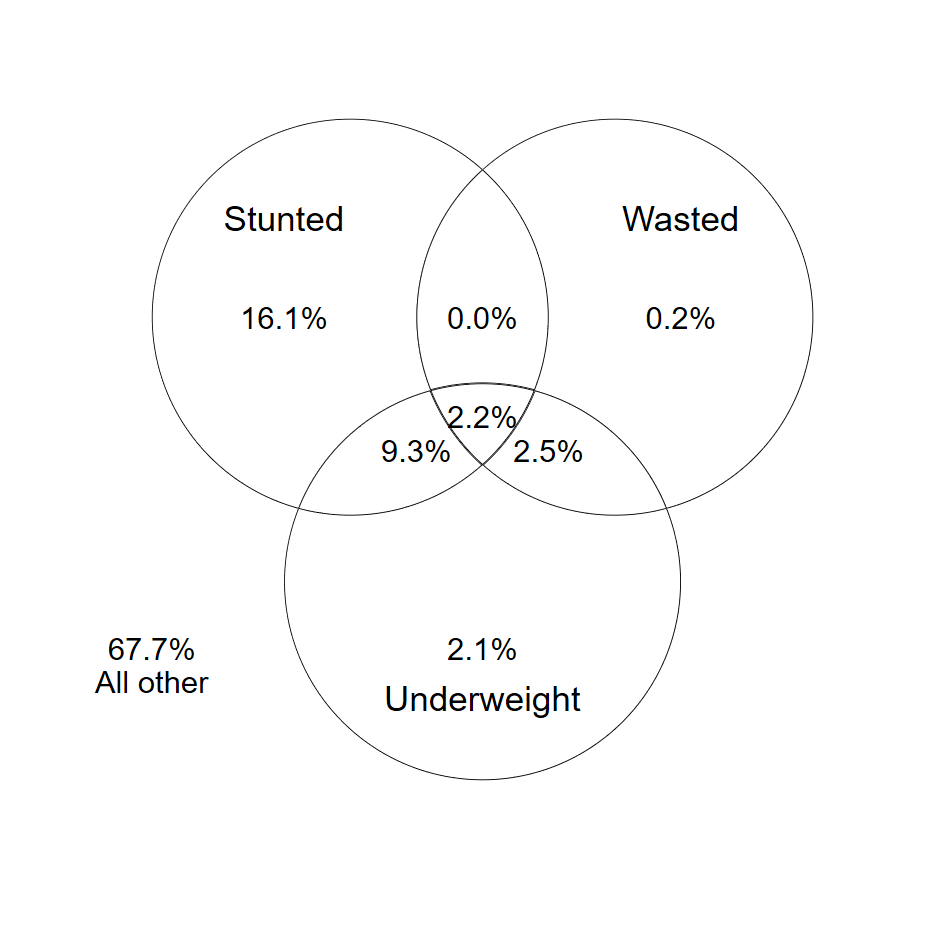 | F. Nepal  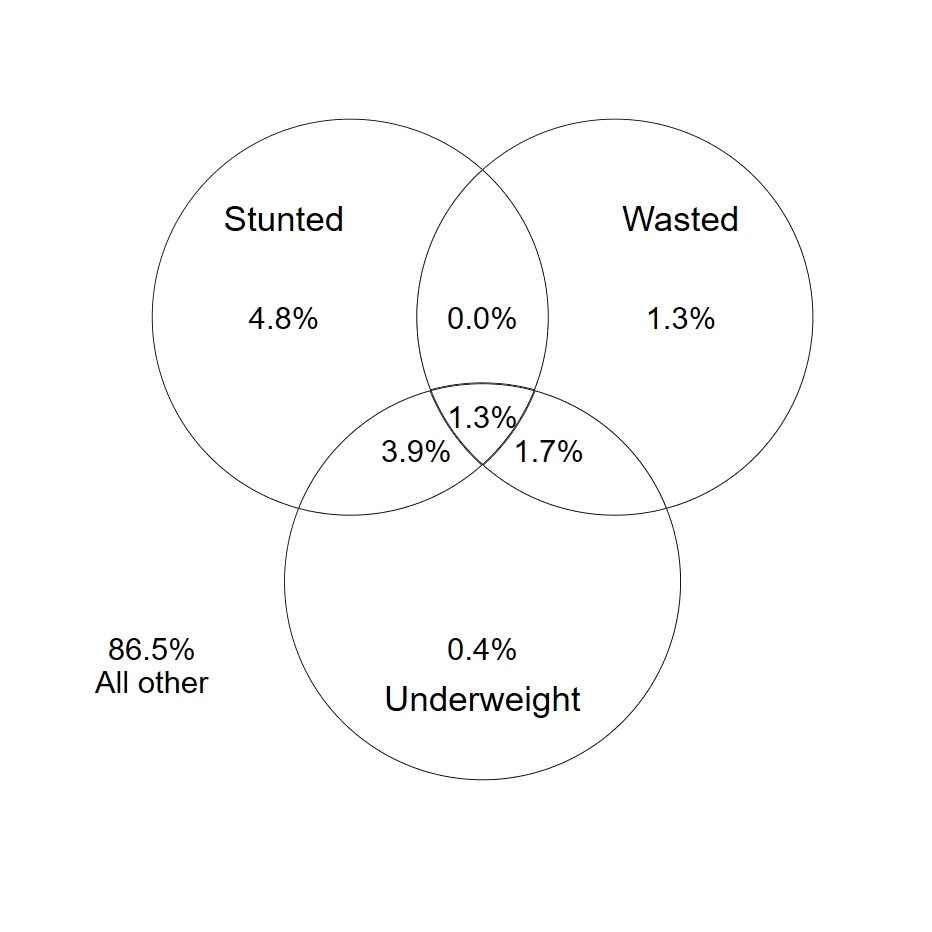 |
| G. Tanzania  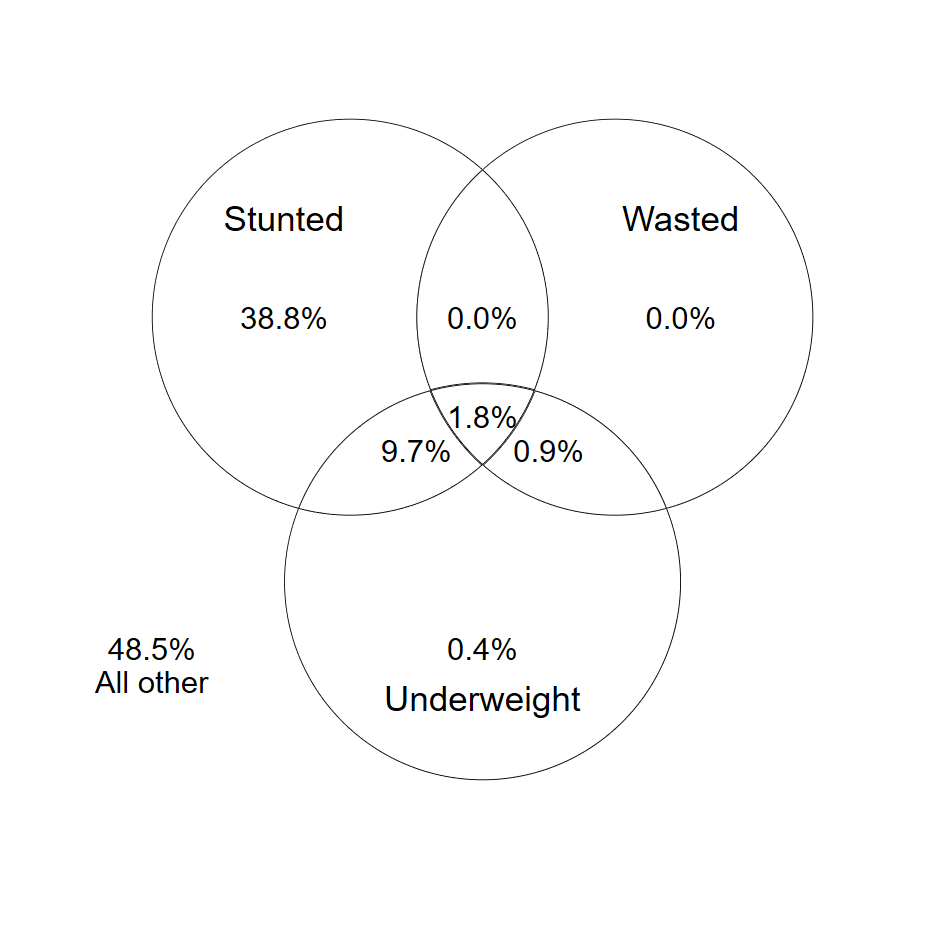 | H. Bangladesh - Mirpur  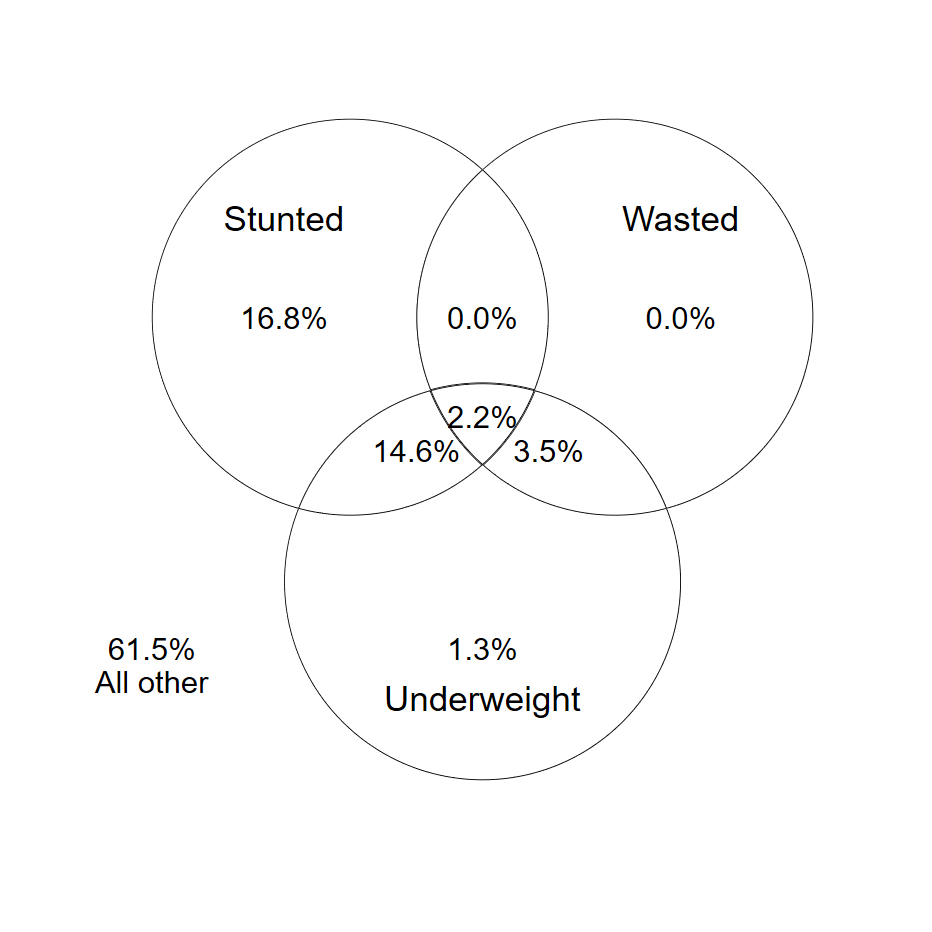 | I. India  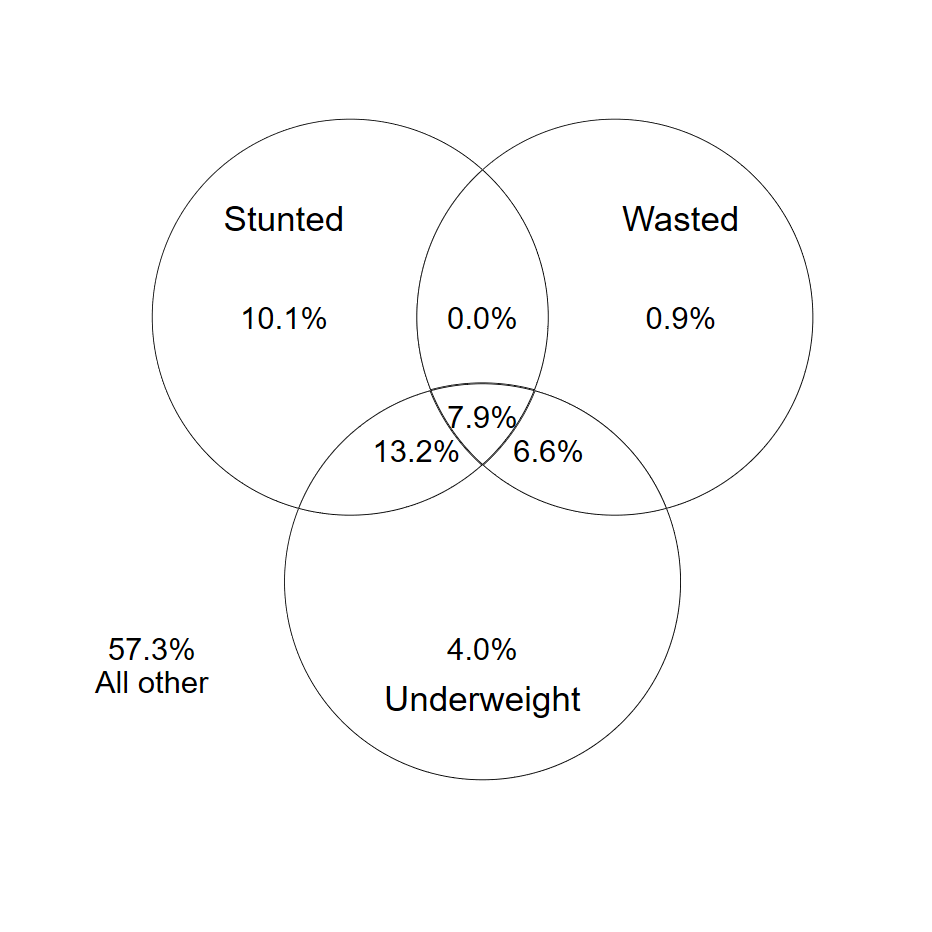 |
| J. Bangladesh - Matlab  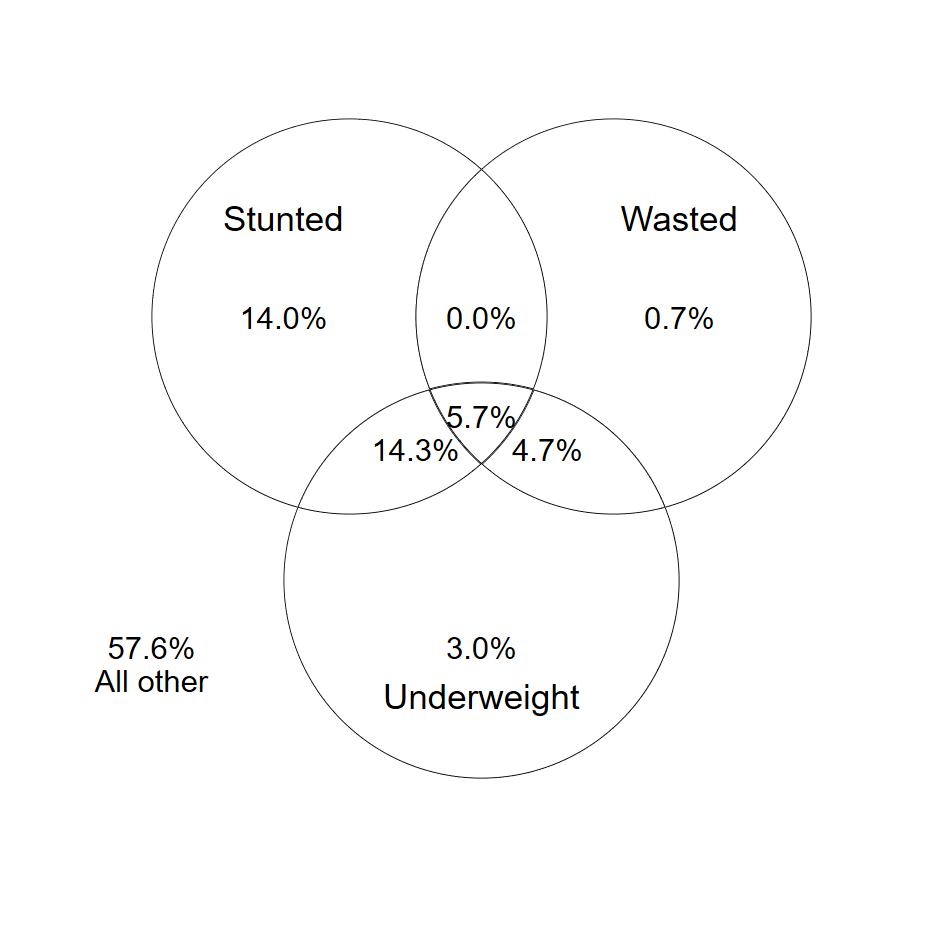 | 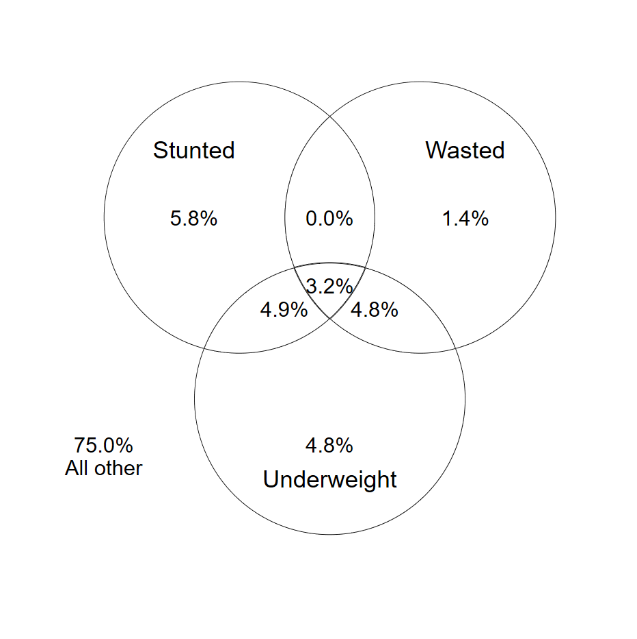K. Burkina Faso |  |

**References**

1. Roelants M, Hauspie R, Hoppenbrouwers K. References for growth and pubertal development from birth to 21 years in Flanders, Belgium. Ann Hum Biol. 2009;36:680–94.

2. Miller M, Acosta AM, Chavez CB, Flores JT, Olotegui MP, Pinedo SR, Trigoso DR, Vasquez AO, Ahmed I, Alam D, et al. The MAL-ED study: A multinational and multidisciplinary approach to understand the relationship between enteric pathogens, malnutrition, gut physiology, physical growth, cognitive development, and immune responses in infants and children up to 2 years of age in resource-poor environments. Clinical Infectious Diseases. Oxford University Press; 2014;59:S193–206.

3. Acosta AM, De Burga RR, Chavez CB, Flores JT, Olotegui MP, Pinedo SR, Salas MS, Trigoso DR, Vasquez AO, Ahmed I, et al. Relationship between growth and illness, enteropathogens and dietary intakes in the first 2 years of life: Findings from the MAL-ED birth cohort study. BMJ Glob Health. BMJ Publishing Group; 2017;2.

4. MAL-ED Network Investigators. Childhood stunting in relation to the pre- and postnatal environment during the first 2 years of life: The MAL-ED longitudinal birth cohort study. PLoS Med. Public Library of Science; 2017;14.

5. Arifeen S El, Ekström E-C, Frongillo EA, Hamadani J, Khan AI, Naved RT, Rahman A, Raqib R, Rasmussen KM, Selling KE, et al. Cohort Profile: The Maternal and Infant Nutrition Interventions in Matlab (MINIMat) cohort in Bangladesh. Int J Epidemiol. 2018;47:1737–1738e.

6. Persson LÅ, Arifeen S, Ekström E-C, Rasmussen KM, Frongillo EA, Yunus M, MINIMat Study Team for the. Effects of Prenatal Micronutrient and Early Food Supplementation on Maternal Hemoglobin, Birth Weight, and Infant Mortality Among Children in Bangladesh. JAMA. 2012;307.

7. Huybregts L, Becquey E, Zongrone A, le Port A, Khassanova R, Coulibaly L, Leroy JL, Rawat R, Ruel MT. The impact of integrated prevention and treatment on child malnutrition and health: the PROMIS project, a randomized control trial in Burkina Faso and Mali. BMC Public Health. BioMed Central Ltd.; 2017;17.

8. Becquey E, Huybregts L, Zongrone A, le Port A, Leroy JL, Rawat R, Touré M, Ruel MT. Impact on child acute malnutrition of integrating a preventive nutrition package into facility-based screening for acute malnutrition during well-baby consultation: A cluster-randomized controlled trial in Burkina Faso. PLoS Med. Public Library of Science; 2019;16.

9. Huybregts L, le Port A, Becquey E, Zongrone A, Barba FM, Rawat R, Leroy JL, Ruel MT. Impact on child acute malnutrition of integrating small-quantity lipid-based nutrient supplements into community-level screening for acute malnutrition: A cluster-randomized controlled trial in Mali. PLoS Med. Public Library of Science; 2019;16.

10. Leroy JL, Ruel M, Habicht J-P, Frongillo EA. Using height-for-age differences (HAD) instead of height-for-age z-scores (HAZ) for the meaningful measurement of population-level catch-up in linear growth in children less than 5 years of age. BMC Pediatr. 2015;

11. World Health Organization, United Nations Children’s Fund. Recommendations for data collection, analysis, and reporting on anthropometric indicators in children under 5 years old. Geneva; 2019.

12. World Health Organisation. Training Course on Child Growth Assessment. Training Course on Child Growth Assessment. Geneva; 2008.

13. World Health Orginazation. Module 9: Care of the well child. Integrated Management of Childhood Illness: distance learning course. Geneva, Switzerland: World Health Organization; 2014.

14. UNICEF. Facts for Life. 4th ed. New York: UNICEF; 2010.

15. Departamento de Capacitación del Ministerio de Salud Pública y Asistencia Social, Programa Nacional de Seguridad Alimentaria y Nutricional, Áreas de Salud de Quezaltenango Totonicapán y San Juan Ostuncalco, gencia  de  los  Estados  Unidos  para  el  Desarrollo  Internacional. Nutrición Materno-Infantil en los Primeros 1,000 Días de Vida: Monitoreo y promoción del crecimiento y desarrollo. 2016 May.

16. Cusminsky M, Lejarraga H, Mercer R, Martell M, Fescina R. Manual de crecimiento y desarrollo del niño, segunda edicion. Washington, DC, USA: OPS (Organizacion Panamericana de la Salud); 1994.

17. Schaetzel T, Griffiths M, del Rosso JM, Plowman B. Evaluation of the AIN-C program in Honduras [Internet]. Arlington, VA, USA; 2008. Available from: www.usaid.gov/our_work/global_health

18. Ghana Health Service, Ministry of Health Ghana. Maternal and Child Health Record Book.

19. Ghana Health Service. Imagine Ghana Free of Malnutrition: A concept paper for addressing malnutrition in Ghana as a developmental problem, using health as an entry point. 2005 May. Report No.: 2.

20. Burkina Faso Ministére de la Santé. Protocoles de Sante de la Reproduction: Santé du nourrisson, de l’enfant, de l’adolescent et du jeune. 2019 Feb.

21. Cellule de Lutte contre la Malnutrition. Programme de Renforcement de la Nutrition: Plan stratégique-Phase II. 2006 May.

22. Federal Ministry of Health E. Training of Health Extension Workers (HEW) On Family Folder and HMIS Procedures Facilitators’ Guide. 2011.

23. Ethiopia Community-Based Nutrition: Training Guide for HW/HEWs [Internet]. Available from: https://www.sdgfund.org/community-based-nutrition-training-guide-health-workers-and-health-extension-workers

24. Federal Democratic Republic of Ethiopia. Government of Ethiopia National Nutrition Program, 2016-2020. 2016.

25. National Institute of Public Cooperation and Child Development. Growth Monitoring Manual. New Delhi, India;

26. Ministry of Health and Family Welfare, Ministry of Women and Child Development. Guidebook for Mother-Child Protection Card for ANM | ASHA | AWW. 2018.
